# Genome deletions to overcome the directed loss of gene function in *Leishmania*

**DOI:** 10.3389/fcimb.2022.988688

**Published:** 2022-09-23

**Authors:** Edubiel A. Alpizar-Sosa, Yasmine Kumordzi, Wenbin Wei, Phillip D. Whitfield, Michael P. Barrett, Paul W. Denny

**Affiliations:** ^1^ Department of Biosciences, Durham University, Durham, United Kingdom; ^2^ Glasgow Polyomics, College of Medical, Veterinary and Life Sciences, University of Glasgow, Glasgow, United Kingdom; ^3^ Wellcome Centre for Integrative Parasitology, School of Infection and Immunity, College of Medical, Veterinary and Life Sciences, University of Glasgow, Glasgow, United Kingdom

**Keywords:** Leishmania, genome, knockout, plasticity, lipids

## Abstract

With the global reach of the Neglected Tropical Disease leishmaniasis increasing, coupled with a tiny armory of therapeutics which all have problems with resistance, cost, toxicity and/or administration, the validation of new drug targets in the causative insect vector borne protozoa *Leishmania* spp is more important than ever. Before the introduction of CRISPR Cas9 technology in 2015 genetic validation of new targets was carried out largely by targeted gene knockout through homologous recombination, with the majority of genes targeted (~70%) deemed non-essential. In this study we exploit the ready availability of whole genome sequencing technology to reanalyze one of these historic cell lines, a *L. major* knockout in the catalytic subunit of serine palmitoyltransferase (LCB2), which causes a complete loss of sphingolipid biosynthesis but remains viable and infective. This revealed a number of Single Nucleotide Polymorphisms, but also the complete loss of several coding regions including a gene encoding a putative ABC3A orthologue, a putative sterol transporter. Hypothesizing that the loss of such a transporter may have facilitated the directed knockout of the catalytic subunit of LCB2 and the complete loss of *de novo* sphingolipid biosynthesis, we re-examined LCB2 in a *L. mexicana* line engineered for straightforward CRISPR Cas9 directed manipulation. Strikingly, LCB2 could not be knocked out indicating essentiality. However, simultaneous deletion of LCB2 and the putative ABC3A was possible. This indicated that the loss of the putative ABC3A facilitated the loss of sphingolipid biosynthesis in *Leishmania*, and suggested that we should re-examine the many other *Leishmania* knockout lines where genes were deemed non-essential.

## Introduction

The Neglected Tropical Disease (NTD) leishmaniasis is endemic in over 90 countries, impacting at least 12 million people per year, with over one billion people living at risk of the disease (WHO, 2019). The causative *Leishmania* species are sand fly borne kinetoplastid protozoan parasites (Stuart et al., 2008) and infection *via* insect bites leads to a wide spectrum of disease, from self-healing but scarring cutaneous leishmaniasis (CL) to fatal visceral disease (VL). This diversity of disease is dependent on the infecting species, and host genetic background and immunity (Reithinger et al., 2007). An amphotericin B drug treatment-based elimination effort in south Asia has, in part, lead to the global burden of VL decreasing substantially in the past decade. However, largely due to forced migration in conflict zones, CL cases have increased dramatically (0.7-1.0 million per year) (Burza et al., 2018). Despite the success of the amphotericin B programme in south Asia, in the absence of vaccines, treatment still relies entirely on a limited number of ‘less than ideal’ drugs with toxicity problems and rising resistance. Notably amphotericin B (Thakur et al., 1999) is associated with severe side-effects and *Leishmania* resistance has been observed in the clinic (Di Giorgio et al., 1999). The pentavalent antimonials - sodium stibogluconate (Pentostam^®^) and meglumine antimoniate (Glucantime^®^) (Croft and Coombs, 2003; Kedzierski et al., 2009) - are the mainstay for CL treatment. Both have been in clinical use for over 70 years despite their severe side-effects (Chappuis et al., 2007), parenteral administration (Demicheli et al., 2004), and the emergence of drug resistance (Croft et al., 2006).

To address this urgent situation, recent public-private partnerships have seen industrial scale (>1,000,000) compound libraries screened for antileishmanials either phenotypically (Pena et al., 2015; Khare et al., 2016) or using target-based approaches (Norcliffe et al., 2018). A target-based approach demands a well validated, typically protein, target – that is essential for parasite fitness such that chemical inhibition will lead to cell death. Genetic-based approaches have typically been employed to perform this function in *Leishmania* spp (Jones et al., 2018) and related trypanosomatids (Chiurillo and Lander, 2021; Horn, 2021), with recent advances in genetic technology such as gene editing facilitating high throughput approaches (Jones et al., 2018; Ferreira et al., 2021; Horn, 2021). However, in the absence of RNA interference in most *Leishmania* spp (Lye et al., 2010), prior to the recent technological advance of gene editing genetic, target validation typically relied on homologous recombination, technology first applied to these protozoa over 30 years ago when a single allele of the dihydrofolate reductase-thymidylate synthase gene (*dhrf-ts*) (LmjF.06.0860 in *L. major*) was replaced with the neomycin phosphotransferase gene (*neo*) in *L. major* which lacked the second *dhrf-ts* allele, yielding DHRF-TS null parasite lines (Cruz and Beverley, 1990). These knockout parasites demonstrated thymidine auxotrophy and required the presence of thymidine for growth, thus demonstrating that DHRF-TS is essential for *L. major* survival in laboratory conditions. Further development of multiple selectable markers (Cruz et al., 1991) led to the expansion of this approach to largely diploid *Leishmania* spp and by 2018 this approach had underpinned the successful or attempted knockout of nearly 200 genes in *Leishmania* spp (Jones et al., 2018). In many instances the attempted deletion of suspected essential genes failed, either the transfected parasites would not survive (in the culture conditions provided) or the cells would duplicate specific chromosomes (or portions of them, i.e. partial or entire deletion/duplication) containing the gene of interest, or maintain it on an ectopic element (Jones et al., 2018). This reflected the plastic nature of the *Leishmania* spp genome which demonstrates a high level of aneuploidy (Rogers et al., 2011; Sterkers et al., 2011; Dumetz et al., 2017). However, most genes studied [approximately 70% (Jones et al., 2018)] were readily deleted using homologous recombination and selectable marker technology. Some of these were surprising, such as ablation of the first, rate limiting step in sphingolipid biosynthesis, serine palmitoyltransferase (SPT), by deletion of the catalytic LCB2 subunit (LmjF.35.0320) of the enzyme complex formed with LCB1 (LmjF.34.3740). The *L. major* LCB2 knockout (*Lmj*LCB2-/-) remained not only viable *in vitro*, but also infective in the complete absence of sphingolipid biosynthesis (Zhang et al., 2003; Denny and Smith, 2004; Denny et al., 2004). This was unlike mammals (Hanada et al., 1998), yeast (Nagiec et al., 1994), plants (Chen et al., 2006) and other trypanosomatids (Fridberg et al., 2008) where SPT activity was found to be essential for full viability. This finding led to a view that sphingolipid biosynthesis might be an unsuitable target for antileishmanial drug development, given the perception that the parasites could survive irrespective of their ability to synthesize sphingolipid.

Given the genome plasticity of *Leishmania* spp discussed above, and the long timeframe for selection employed during this technique (typically 4-6 weeks for the deletion of each allele), it was considered possible that compensatory mutations and other genomic changes could have been a necessary event in allowing deletion of LCB2. To examine this, we sequenced the whole genome of the clonal *Lmj*LCB2-/- population studied by Denny et al. (Denny et al., 2004) alongside its equivalently cultured parent (*L. major* FV1). The results demonstrated that additional non-targeted gene deletions occurred both during the selection of the *Lmj*LCB2-/- and, most likely, during passage in a murine model. We consider the function and test the roles of these deleted genes, revealing that compensatory deletions can be necessary to allow loss of otherwise essential genes. Many knockout *Leishmania* spp lines, generated using homologous recombination with protracted selection procedures, may require reanalyzes to determine the presence and effect of compensatory genomic changes.

## Materials and methods

### Leishmania culture

*L*. *major* wild type (FV1) (MHOM/IL/81/Friedlin; FV1 strain and serine palmitoyltransferase mutant (*Lmj*LCB2-/-) (Denny et al., 2004), the parental *L. mexicana* strain T7 Cas9 (Beneke and Gluenz, 2019) and all mutants generated were cultured as axenic promastigotes at 26°C in complete Schneider’s insect medium (SIM) (pH 7; Sigma Aldrich) supplemented with 10% heat-inactivated foetal bovine serum (HI-FBS) (Gibco) and 100 μg mL^-1^ streptomycin and 100 IU mL^-1^ penicillin (Sigma Aldrich). For *L. mexicana* relevant selective drugs were added to the medium at the following concentrations 32 µg ml^−1^ hygromycin B, 20 µg ml^−1^ puromycin dihydrochloride, 5 µg ml^−1^ blasticidin S hydrochloride, 40 µg ml^−1^ G-418 disulfate and 50 µg ml^−1^ nourseothricin sulfate (Melford Laboratories Ltd).

### Whole genome sequencing

Genomic DNA was extracted with the Nucleospin^®^ Tissue kit (Macherey-Nagel) from the parental wild type of *L*. *major* (FV1) and the serine palmitoyltransferase knockout (*Lmj*LCB2-/-) cell lines. Mid-log promastigotes (1 x 10^7^) cell pellets were washed twice in PBS at 1,250 g for 10 minutes. Library prep and sequencing were performed at Glasgow Polyomics using Illumina sequencer NextSeq 500 yielding 2 x 75 bp paired-end reads from both (for general statistics see **S1 Table**). Reference genomes of *L. major* strain Friedlin (MHOM/IL/81/Friedlin) release 54 were obtained from TriTrypDB (http://tritrypdb.org). Reads were mapped to the reference genomes using BWA-MEM (Li, 2013) and PCR duplicates were removed using GATK version 4.2.3.0 (McKenna et al., 2010). Variant calling was performed using MuTect2 (Benjamin et al., 2019) with the default settings and variants were annotated using snpEff (Cingolani et al., 2012). Filtered Variant Call Format (VCF) files corresponding to SNPs and indels with an impact on coding sequences (or intergenic regions in some instances) present in *Lmj*LCB2-/- but not in the wild-type parent were compared and manually verified using the IGV 2.8.9 visualization tool (Robinson et al., 2017).

Copy ratio alterations were detected using GATK (version 4.2.3.0). Reference genomes were divided into equally sized bins of 1000 base pairs using PreprocessIntervals tool. Read counts in each bin were collected from alignment data using CollectReadCounts tool. Copy number ratio was obtained by comparing the mutant line (*Lmj*LCB2-/-) with the corresponding copy number of the parent used as the base line. Genome contigs were segmented using ModelSegments tool from copy ratios of mutant and wild type samples. Amplified and deleted segments were identified using CallCopyRatioSegments tool with the default settings. Plots of denoised and segmented copy-ratios were generated using R software. Forward and reverse sequencing fastq raw data files from wild type (FV1), LmajorFVI-1_S1_R1_001.fastq and LmajorFVI-1_S1_R2_001.fastq, and *Lmj*LCB2-/-, Lmj_LCB2_S8_R1_001.fastq.gz and Lmj_LCB2_S8_R2_001.fastq.gz were deposited at the NCBI National Center for Biotechnology Information (https://www.ncbi.nlm.nih.gov) with project PRJNA771089. Gene ontology enrichment and identification of gene ID’s and names of variants filtered with WGS was performed using OrthoMCL (https://orthomcl.org/orthomcl/app) and TriTrypDB (https://tritrypdb.org/), respectively. Protein sequences were retrieved from TriTrypDB and Uniprot (https://www.uniprot.org/) or from the Protein Data Bank (PDB) (https://www.rcsb.org/) and alignments were performed using ClustalW Omega (http://www.clustal.org/omega/).

### CRISPR-Cas9 gene knockouts

Gene deletions were performed as previously described (Beneke et al., 2017). The online tool (www.LeishGEdit.net) was used to design primers for amplification of the 5′ and 3′ sgRNA templates and for amplification of donor pT plasmids for knockouts. This tool selects appropriate regions upstream and downstream of the target coding region and designs the sgRNA template and primers for amplification of the homology directed repair construct with 30 nucleotide homologous regions. Oligonucleotides were purchased from Integrated DNA Technologies (IDT; **Table 1**). *L. mexicana* Cas9 T7 cell line was maintained as promastigotes (1 x 10^7^/mL) in the presence of nourseothricin sulphate (50 μg/mL) and hygromycin B (32 μg/mL) as described elsewhere (Beneke et al., 2017), followed by transfection with 10 μg of each respective donor DNA and sgRNAs using 2 mm gap cuvettes (MBP) with program X-001 of the Amaxa Nucleofector IIb (Lonza Cologne AG, Germany). Following transfection, parasites were transferred into 5 mL prewarmed medium in 25 cm^2^ flasks and left to recover overnight at 26°C. Then limiting dilution, in the presence of appropriate selection drugs, was used to generate clonal knockout cell lines. The LmxM.11.1240 knockout (LmxM.11.1240-/-) was selected with 20 µg ml^−1^ puromycin dihydrochloride, and the LmxM.11.1240 and LmjF.35.0320 knockout (LmxM.11.1240-/- LmjF.35.0320-/-) was selected with 20 µg ml^−1^ puromycin dihydrochloride, 5 µg ml^−1^ blasticidin S hydrochloride and 40 µg ml^−1^ G-418 disulfate. Survival of selected transfectants became apparent 7–10 days after transfection. Deletion of both copies for each gene was confirmed by PCR (**Table 2**; **Figure 4**). Cells were imaged using an Olympus CKX53SF-1-2 inverted microscope, counted using a Neubauer Haemocytometer and images captured with a Ceti WFHD5C 5MP digital microscope camera (**Figure 5**).

**Table 1:** Primers used for CRISPR Cas9 disruption of repair of LmX.11.1240 and LmX.34.0320.

	LmxM.11.1240	LmxM.34.0320
5’ sgRNA primer	gaaattaatacgactcactataggGGAGCGTCAGCGTGGCAACAgttttagagctagaaatagc	gaaattaatacgactcactataggAGCACACGCACAAAAAAAGCgttttagagctagaaatagc
3’ sgRNA primer	gaaattaatacgactcactataggTGGCAACTGCGTGAGTGCAAgttttagagctagaaatagc	gaaattaatacgactcactataggAGTGTGTTGGGCGGCAGCCGgttttagagctagaaatagc
5’ repair primer	GGTGTGGTGCCTTCTTGTTTTTTTTTGCCGgtataatgcagacctgctgc	TCAAGTCAGTGAGCAGCAGCATATAGGAGAgtataatgcagacctgctgc
3’ repair primer	CAGCTCCTTCCACGAGTAGAGCCGCGCCCGccaatttgagagacctgtgc	GTGAGTGTGTGATGCTGAACAGTGTGTCCTccaatttgagagacctgtgc

**Table 2:** Primers used for PCR diagnostics of LmX.11.1240 and LmX.34.0320 knockouts.

P1/P2	ATGGAGCCGGTGACCACCGCCG	CTAGCGTAGATTCATCGCAAGTCCCCGAACCTTCAT
P3/P4	CATTGCATGGAAGTGAGTGCC	CGCATCAACCACTGAAGCAT
P5/P6	ATGAGCGAGGCGGCGCTGAA	TTACCGCAGCGGGTTCGTGCT
P7/P8	TTTCCTGTATGCGTGTGTGC	GGAACTACACTGGTCATGGCA

### Lipid analyses by liquid chromatography-mass spectrometry

As previously (Denny et al., 2004), cell pellets (~1 x 10^9^ parasites) were extracted twice for 2h with 250 µl of chloroform–methanol–water (4:8:3, v/v/v) in a sonicating water bath. After centrifugation, the pooled supernatants were adjusted to a biphasic mixture of chloroform–methanol–water (4:8:5.6), vortexed and centrifuged. The lower chloroform phase was collected, evaporated to dryness under nitrogen gas and reconstituted in methanol. Global lipidomic analysis was performed by high resolution liquid chromatography-mass spectrometry (LC-MS) using an Exactive Orbitrap mass spectrometer (Thermo Scientific, Hemel Hempsted, UK) interfaced to a Thermo UltiMate 3000 RSLC system. Samples (10 µl) were injected onto a Thermo Hypersil Gold C18 column (1.9 µm; 2.1 mm x 100 mm) maintained at 50°C. Mobile phase A consisted of water containing 10 mM ammonium formate and 0.1% (v/v) formic acid. Mobile phase B consisted of a 90:10 mixture of isopropanol-acetonitrile containing 10 mM ammonium formate and 0.1% (v/v) formic acid. The initial conditions for analysis were 65%A-35%B, increasing to 65%B over 4 min and then to 100%B over 15 min, held for 2 min prior to re-equilibration to the starting conditions over 6 min. The flow rate was 400 µl/min. Samples were analyzed in positive and negative-ion modes at a resolution of 100,000 over the mass-to-charge ratio (m/z) range of 250 to 2,000. Analyses were performed in negative ion mode at a resolution of 100,000 over the mass-to-charge ratio (m/z) range of 250 to 2,000. Progenesis QI v3.0 (Nonlinear Dynamics, Newcastle upon Tyne, UK) was used to process the data sets and determine the relative intensities of signals associated with ceramide and inositol phosphorylceramide (IPC) molecular species. Graphs were plotted with Prism v9.4 (GraphPad, San Diego, USA).

## Results

### Whole genome sequencing

**Figure 1:**
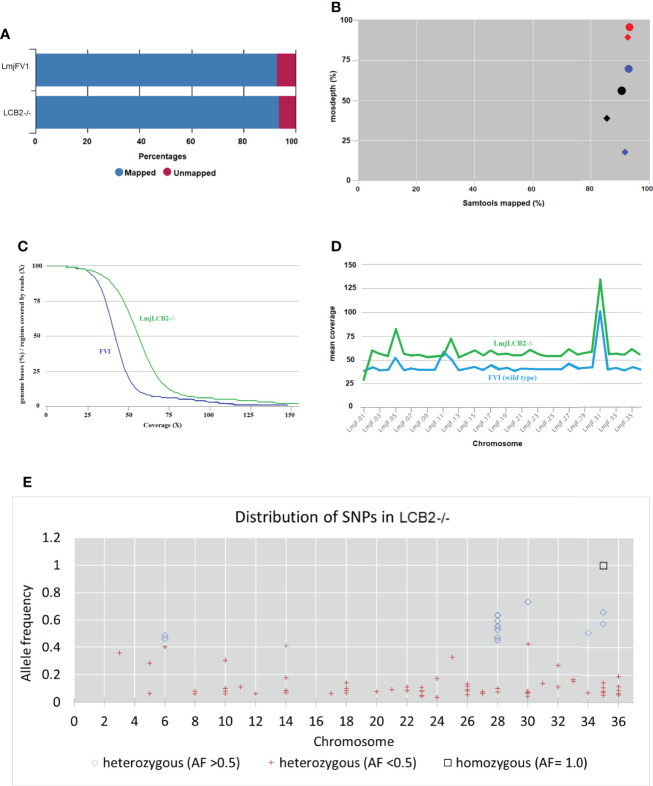
WGS Samtools parameters in FVI (wild type) and *Lmj*LCB2-/-. **(A)** Bar chart showing mapped and unmapped alignment scores (%) in both LmjLCB2-/- and FVI. **(B)** Scatterplot showing the correlation between mapped reads (%) (*x*-axis) and coverage (%) (*y*-axis). Data shows the median coverage (black), and the fraction of the genome with at least ≥30x (red) and ≥50x (blue) coverage as estimated with mosdepth (performs coverage depth for WGS, exome, or targeted sequencing) in *Lmj*LCB2-/- (circles) and wild type (FVI) (diamonds). **(C)** Distribution of coverage depth with the number of locations in the reference genome with a given depth of coverage. **(D)** Mosdepth average coverage per contig or chromosome in FVI and *Lmj*LCB2-/-. **(E)** Distribution of homozygous and heterozygous SNPs in LmjLCB2-/- (not present in the parental wild type) based on their allele frequency (Pedersen and Quinlan, 2018). See Materials and Methods and **S1** **and** **S2 Tables** for full details.

Our earlier work allowed the selection of a verified null mutant of LCB2 (*Lmj*LCB2-/-), a gene that initiates the sphingolipid biosynthetic pathway in *L. major* (Denny et al., 2004). The result was surprising as it had been assumed the gene product would be essential. The plasticity of the genome, however, allows *Leishmania* spp to adapt to different conditions through a multitude of compensatory mutations (Sterkers et al., 2012). With the advent of fast and inexpensive whole genome sequencing we therefore revisited to the genome of the *Lmj*LCB2-/- to seek non-targeted genomic changes that may account for the ability to tolerate the deletion of LCB2 (LmjF35.0320). Both *Lmj*LCB2-/- and its wild type parent (*L. major* FV1) were clonal lines that had been passaged in BALB/c mice before being recovered and stored (Denny et al., 2004). A FastQC report showed that the total number of sequences (35-76 base pairs) for the wild type LmjFV1 and *Lmj*LCB2-/- genomes were 11,944,511 and 16,747,081 reads respectively with mean quality score >30 across all bases and zero reads flagged with poor quality. Notably, over 90% of reads mapped to the reference genome (**Figure 1A**) with a median coverage of 41x and 56x for FV1 and *Lmj*LCB2-/-, respectively (**Figure 1B**). In keeping with this, the distribution of coverage (**Figure 1C**) and average coverage per contig or chromosome (**Figure 1D**) were higher in *Lmj*LCB2-/- than in the parental line FV1.

Analyses of these data revealed a number of polymorphisms (n= 358) in 26 chromosomes with no variants found in chromosomes 1, 2, 4, 7, 9, 13, 15, 16, 19 and 29 (**Figure 1E**). Interestingly, in chromosome 35, a singleton (a mutation with allele frequency of 1 present in all reads) was found in the intergenic region (non-coding) of four genes (LmjF.35.0300, LmjF.35.0310, LmjF.35.0320 and LmjF.35.0330) in the *Lmj*LCB2-/- genome. The latter two of these (LCB2 and 3-dehydrosphinganine reductase) are part of the sphingolipid biosynthetic pathway. It could be speculated that this SNP was a result of the homologous recombination driven deletion of LCB2 (LmjF.35.0320) in this line. 9.8% of the variants found in the *Lmj*LCB2-/- genome were protein altering SNPs (**Table 3**) distributed across sixteen chromosomes. A detailed list containing all mutations in both intergenic (IGR) and coding regions (CDS) is provided in **S2 Table** and **S3 Table** respectively.

**Table 3:** Variants in the *Lmj*LCB2-/- genome identified using WGS. See **S2 Table** and **S3 Table** for a detailed list with all mutations.

Mutation type	frequency	protein altering
5’ or 3’ Flank	270	0
Intergenic region (IGR)	45	0
Coding sequence (CDS)	14	14
(missense)	13	13
(non-sense or stop gained)	1	1
Frame shift Indel	16	16
In Frame indel	**5**	**5**
(disruptive)	3	3
(conservative)	2	2
RNA	**6**	0
Silent	**2**	0
Total	358	35

**Figure 2:**
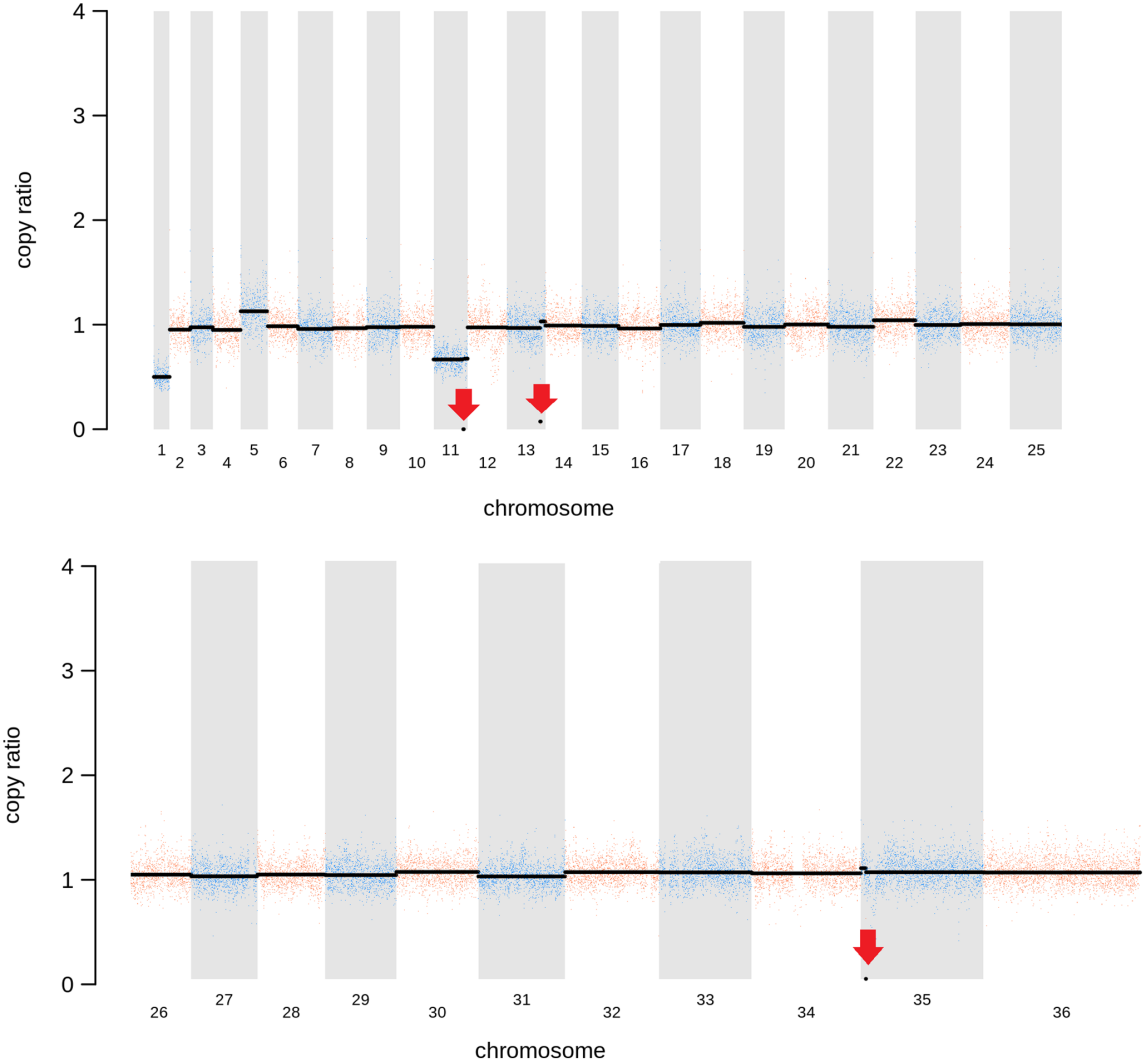
Copy Number Variations (CNVs) in *Lmj*LCB2-/-. Diagram showing the copy number ratio in chromosomes 01, 11, 13 and 35 (decrease) and 05 (increase) with no change for most chromosomes. The copy ratio median (thick black lines) is shown in log2 ratio (y-axis) for all the chromosomes (x-axis). As detailed in **Table 4**, the alterations affected 100% and 97.5% of chromosomes 01 and 05 respectively. The changes observed in the other chromosomes (11, 13 and 35) were more heterogenous, therefore the position of the gene(s) deleted within each chromosome is highlighted (red arrows). Copy ratio alterations were detected comparing the mutant line (*Lmj*LCB2-/-) with the corresponding copy number of the parent used as the base line. Plots of denoised and segmented copy-ratios were generated using R. See **S4** **and** **S5 Table** for a detailed list of these changes and section 2.2 for a full description of analyses.

As expected, in the clonal *Lmj*LCB2-/- line, the LCB2 gene (LmjF.35.0320) is unambiguously deleted and replaced by hygromycin and puromycin selectable markers (Denny et al., 2004; Armitage et al, 2018). However, aneuploidy was also observed with chromosome 1 being present as a single copy (mean log2 copy ratio: -0.999081), whilst chromosome 5 has three copies (mean log2 copy ratio: 0.173596) (**Table 4**). The wild-type parent is diploid for both of these (**S4 Table** **)**. Aneuploidy has previously been observed as a contributing factor to diversity in *Leishmania* (Rogers et al., 2011) and is associated with drug resistance (Mina et al., 2021). It is possible that it had an unknown role here in facilitating pathogen growth and pathogenicity in the absence of sphingolipid biosynthesis. However, furthermore and surprisingly, in addition to the targeted deletion of LCB2 in *Lmj*LCB2-/- and the variants above mentioned (**Table 3**), multiple non-targeted gene deletions were noted which were not present in the wild-type parent, *L. major* FV1 (**Figure 2** , **S5 Table** **).**

**Table 4:** Ploidy in *Lmj*LCB2-/- of *Leishmania major*.

Contig	Chr size (bp) *	Chr coordinates		log2 copy ratio to WT	Call	gain/loss size (bp)	Chr affected (%)		Genes affected
		start	end				loss	gain	
LmjF.01	268988	1	268988	-0.99	deleted	268,987	100.0		multiple **
LmjF.05	465823	1001	455000	0.17	amplified	453,999		97.5	multiple **
LmjF.11	579000	1	497000	-0.58	deleted	496,999	85.8		multiple **
		508001	518000	-29.89	deleted	9,999	1.7		LmjF.11.1240 (5727 bp)
		529001	582573	-0.56	deleted	53,572	9.3		multiple **
LmjF.13	654595	565001	574000	-3.76	deleted	8,999	1.4		LmjF.13.1530 (3294 bp) & LmjF.13.1540 (1464 bp)
LmjF.35	2090474	85001	88000	-10.79	deleted	2,999	0.1		LmjF.35.0320 (1617 bp)

*Chromosome (Chr) size and gene IDs were retrieved from TryTripDB (https://tritrypdb.org). **See **S4** and **S5 Table** for a complete list.

One of these, the gene encoding the amastin-like protein (LmjF.08.0740), a surface protein found specifically on mammalian amastigotes (the infective intracellular stage in mammals), is one of a large family of genes (N= 17) arrayed along chromosome 8 (LmjF.08.0670, 0680, 0690, 0700, 0710, 0720, 0730, 0740 [the one possibly deleted], 0750, 0760, 0770, 0800, 0810, 0820, 0830, 0840 and 0850). A related large gene array is also found on chromosome 34, in addition to smaller clusters elsewhere (Rochette et al., 2005) [https://tritrypdb.org]. Interestingly, the region upstream from this large family of amastins, comprising three genes, including an amastin surface glycoprotein (LmjF.08.0640), a DnaJ domain (LmjF.08.0650) and a protein kinase (LmjF.08.0660), showed good coverage and reads with mapping quality (MQ) of 60. Conversely, an extensive region (~107 kb) starting from position 283,000 comprising all the amastin-like genes above mentioned showed lower coverage interspaced with areas of zero coverage in (MQ = 0 for most reads) (**S1 Figure**). Notably, only three genes, two amastins (LmjF.0810 and 0820) and three tuzin putative genes (LmjF.08.0780, 0790 and 0795) located within this region showed good coverage (reads with MQ= 43 to 60). Sequence similarity analysis showed that these amastins conform to three groups. The first group includes 12 genes from LmjF.08.0670 to 0770, 0800 and 0840, which are highly similar to LmjF.08.0740 (the one possibly deleted). In a second group formed by two more divergent genes, LmjF.0810 and 0820, higher coverage and MQ is observed, while LmjF.08.0640 appeared alone in the third group (**S2 Figure**) (Thompson et al., 1994). The absence of coverage in the coding sequence of LmjF.08.0740 is consistent with a deletion while the poor coverage found across this amastin-like-rich region was equally present in both *Lmj*LCB2-/- and its WT FV1 progenitor (**S1 Figure**). Moreover, CNV in chromosome 08 was neutral (**S4 Table**). Therefore, it was considered that this was not a genuine single copy deletion but was missed during the genome assembly process and therefore was not pursued further.

**Figure 3:**
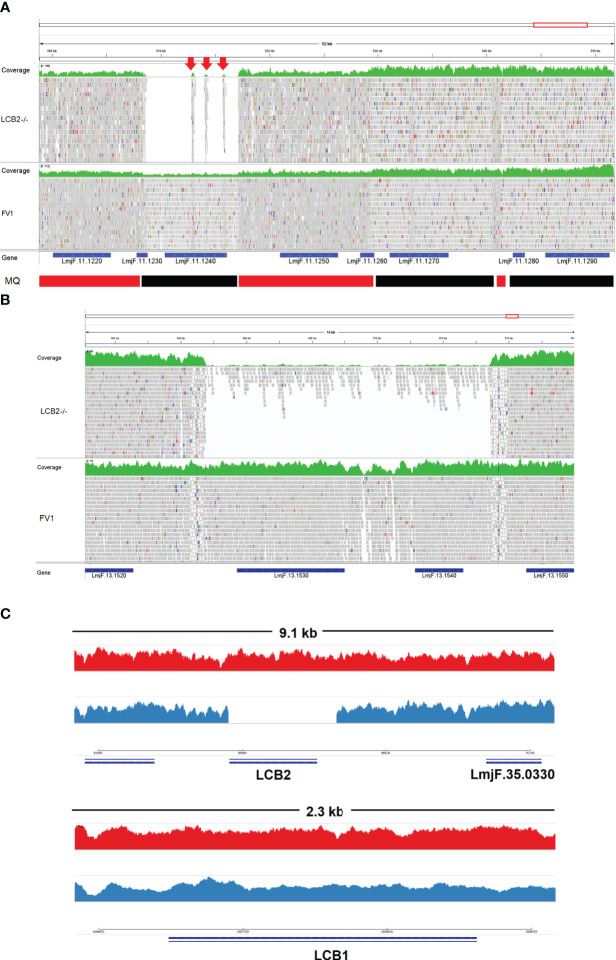
Visualization of the genomic region of non-targeted **(A, B)** and targeted gene deletions in *L. major* knockout (*Lmj*LCB2-/-; C). A and B top (green) represent coverage, whereas A and B bottom show individual reads with mapping quality (MQ) of zero (white-filled bars) or between 40 and 60 (grey-filled bars) in FV1 (wild type) and *Lmj*LCB2-/- cell lines. The CDS (genes) are shown underneath A and B (blue bars). In A, the bottom banner indicates local read positions with mapping quality (MQ) from zero to <30 (red bars) or ≥60 (black bars). In *Lmj*LCB2-/-, the absence of reads alongside three mis-mapped interspersed contigs (red arrows) is shown in *Lmj*F.11.1240 **(A)**, and in *Lmj*F.13.1530 (miltefosine transporter) and adjacent gene LmjF.13.1540 (unknown function) **(B)**. Cartoon showing coverage in FV1 (red) and *Lmj*LCB2-/- (blue) of the two sub-units of serine palmitoyltransferase (SPT). Complete ablation of LmjF.35.0320 (LCB2 subunit) is shown in *Lmj*LCB2-/- but not in FV1 (**C**, top) whilst *Lmj*F.34.3740 (LCB1 subunit) is unaltered in both cell lines (**C**, bottom). WGS data from *L. major* wild type (FV1) and *Lmj*LCB2-/- were aligned to the reference genome (https://tritrypdb.org) and the image was produced using IGV_2.8.9 (Robinson et al., 2017) (http://software.broadinstitute.org).

A gene encoding the putative ABC protein subfamily A, member 3 (LmjF.11.1240; ABCA3 from here on) was of more interest. Reads of ABCA3 in the wild type (FV1) showed good mapping quality (MQ= 60), whilst in *Lmj*LCB2-/-, ABCA3 was completely deleted. Three interspersed contigs of ~250 bp assigned to ABCA3 appeared within the coding sequence (**Figure 3A**, red arrows). However, these reads showed MQ of zero and were probably miss-mapped and a BLASTN search in the GenBank database with a random selection of these reads (n=20) as a query detected similarity scores of 134, E-value: 2e-32 to ABCA3. However, similar hits were found to neighboring genes: LmjF.11.1220, 1250, 1270 and 1290, which are annotated as putative ATP-binding cassette protein subfamily A members and comprise part of a 50 Kb array, encompassing LmjF.11.1240 (ABCA3), of highly similar genes (LmjF.11.1240; **S6 Table**). This indicated that these reads were misannotated to multiple locations. Protein alignment (Clustal Omega) showed that LmjF.11.1240 clusters with LmjF.11.1220, LmjF.11.1250, LmjF.27.0970 and LmjF.27.098, and to a lesser extent with LmjF.11.1270 and 1290. Other members of this family, present on chromosomes 2, 15 and 29 (**S3 Figure**), however, have diverged more with respect to their sequence. Notably, coverage of a 12.2 kb region (positions 496,430 to 508,700) comprising a gene and a pseudogene immediately upstream (LmjF.11.1220 and 1230) and downstream (LmjF.11.1250 and 1260) from LmjF.11.1240 (ABCA3), showed poor mapping quality (MQ= 0 for most reads). The function of ABCA3 has not been studied directly in *Leishmania* spp, although over-expression of an *L. major* Ser/Thr kinase (LmjF.22.0810) did lead to suppression of LmjF.11.1240 expression, aminoglycoside resistance and some hyper-susceptibility to other antileishmanials including amphotericin B and miltefosine (Vacas et al., 2019). Conversely, in clinical *Leishmania (Viannia)* isolates, over-expression of an ABCA3 orthologue has been reported to correlate inversely with miltefosine resistance and intracellular survival (Obonaga et al., 2014). However, in mammalian cells ABCA3 proteins are implicated in the transport of lipids and in sterol homeostasis (Pasello et al., 2020). Loss of this transporter could, therefore, have profound implications for the content of the outer leaflet of the plasma membrane, the primary location of complex sphingolipids – including the inositol phosphorylceramide (IPC) in *Leishmania* spp (Denny et al., 2001; Denny et al., 2006), and other lipid species perturbed in *Lmj*LCB2-/- (Denny et al., 2004; Armitage et al, 2018). Notably, in *Lmj*LCB2-/- cells the base sphingolipid ceramide and IPC (Denny et al., 2004) are missing and it is possible, therefore, that the loss of the ABCA3 transporter could compensate for sphingolipid loss by perturbing normal sterol trafficking across the plasma membrane, thus enabling tolerable membrane fluidity in the absence of a key regulator of this process, the sphingolipid IPC (Denny et al., 2001).

The deleted region covering LmjF.13.1530 and an adjacent gene LmjF.13.1540 in chromosome 13 presented a different profile. Coverage across the region (~9 kb) on chromosome 13 spanning both coding sequences showed reads with mapping quality of 60 in both FV1 and *Lmj*LCB2-/-. However, a low level of mapping in the region comprising the two genes, the miltefosine transporter (LmjF.13.1530; MT, hereon) and the adjacent downstream gene of unknown function (LmjF.13.1540), showed evidence for incomplete ablation. The CNV in this locus confirmed deletion in this region occurred in approximately 93% of the genomes. (**Table 4**, **S5 Table**) with a large majority of reads having a unique mapping locus but were present at a reduced abundance in *Lmj*LCB2-/- (**Figure 3B**). The most plausible interpretation of this is that the sample consists of two sub-populations, one in high abundance with a deletion covering the region, and a second low abundance sub-population retaining the region. This may be due to a differential growth effect, with the population losing the region having a higher growth rate and hence overgrowing the progenitor population. LmjF.13.1530 is annotated as a phospholipid transporting ATPase 1-like protein and is known to encode the miltefosine transporter (MT) where multiple studies have demonstrated that its mutation or loss leads to resistance to miltefosine through diminished drug uptake (Seifert et al., 2003; Zhang and Matlashewski, 2015; Espada et al., 2021). The ‘natural’ role of this complex is as a phospholipid transporter, bringing lipid species from the outer to the inner leaflet of the plasma membrane (Perez-Victoria et al., 2006) and having a role in membrane symmetry and, therefore, in the modulation of fluidity and function that is dependent upon lipid content (Yeboah et al., 2021). Previous work showed that inactivation of the adjacent gene, LmjF.13.1540 (encoding a hypothetical protein), in a miltefosine resistant mutant of *L. major*, was not associated with alteration of miltefosine resistance levels (Coelho et al., 2012), and so presumably is not part of that complex. Although, interestingly, a similar deletion spanning both genes (ortholgoues of LmjF.13.1530 and LmjF.13.1540) was described in clinical isolates of *L. donovani* resistant to antimonials which were selected for miltefosine resistance *in vitro* (Shaw et al., 2016). In addition, several amphotericin B resistant laboratory strains of *L. mexicana* showed an identical deletion spanning both genes (~9 kb) in three lines, while in one line this deletion was more extensive (19 kb) resulting in the deletion of four contiguous genes (**S4 Figure**) (Fernandez-Prada et al., 2016; Pountain et al., 2019; Alpizar-Sosa et al., 2021).

The loss of LCB2 in the *Lmj*LCB2-/- clonal parasite line examined (Denny et al., 2004; **Figure 3**) was thus accompanied by other, non-targeted deletions in three genes. The complete loss of the putative ABCA3 orthologue (LmjF.11.1240) from diploid chromosome 11 is intriguing and could play a major role in lipid transport and the maintenance of membrane fluidity. This suggested that the loss of the product of this gene may have played a facilitative role in the deletion of the gene encoding LCB2, the catalytic subunit of SPT an essential enzyme in all other eukaryotic systems studied to date. By contrast, the two genes (LmjF.13.1530 and LmjF.13.1540) arrayed in tandem in a region of chromosome 13 were only deleted in a sub-group of the population which developed after the initial cloning stage, suggesting that the loss of these genes, the former of which encodes MT, whilst favorable for growth was not essential for the survival of the sphingolipid-free *L. major Lmj*LCB2-/-. Whether any of these changes were critical in the initial *Lmj*LCB2-/- selection process or during subsequent passage in the murine model (Denny et al., 2004) is unclear. However, these data strongly suggested that the non-targeted deletion of several genes conferred a selective advantage for *Lmj*LCB2-/- *L. major* either in culture or during murine infection.

### Functional analysis of potential role of the putative ABC3A in facilitating the deletion LCB2

To facilitate rapid functional analyses of the identified genes, the Cas9 expressing *L. mexicana* line engineered for rapid gene knockout using CRISPR Cas9 technology (Beneke and Gluenz, 2019) was utilized. In this process, two drug selectable markers are integrated simultaneously, and the homozygous knockout population is then selected.

**Figure 4:**
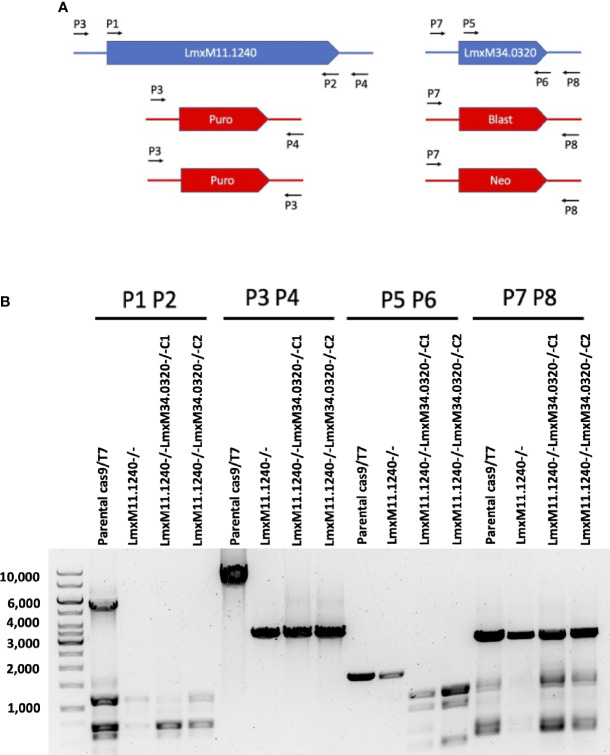
CRISPR Cas9 mediated knockout of LmxM.11.1240 and LmxM.34.0320. **(A)** Illustrates a schematic of the approach taken and the primers P1 to P8 (see section 2.3 for a detailed list) employed for analyses. **(B)** Shows the diagnostic PCR results from the knockouts of LmxM.11.1240 (LmxM.11.1240-/-) and LmxM.11.1240/LmxM.34.0320 (LmxM.11.1240-/-LmxM.34.0320-/-) following selection in Blasticidin/G418 and/or Puromycin and single cell cloning. Puro is the puromycin resistance cassette (1.8bk), Blast the Blasticidin (1.7kb) and Neo the G418 (1.75kb). As illustrated the sizes of the Blast and Neo cassettes are indistinguishable under the fractionation conditions employed, 1% agarose gel. Images are from one representative biological replicate.

**Figure 5:**
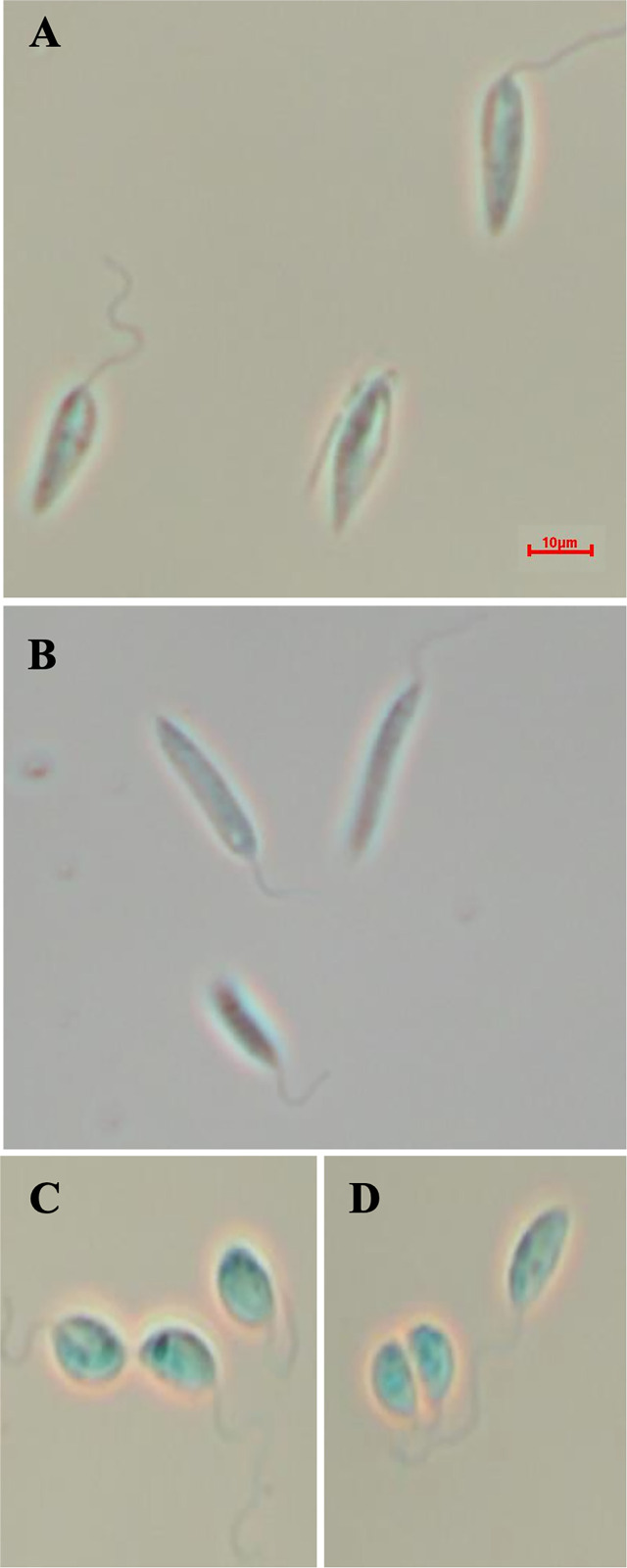
Morphology of the of LmxM.11.1240 and LmxM.11.1240/LmxM.34.0320 knockouts. **(A)** Parental line, *L. mexicana* strain T7 Cas9; **(B)** LmxM.11.1240 (ABCA3) knockout; **(C, D)** Clone 1 and 2 of the LmxM.11.1240 and LmxM.34.0320 double knockout. Scale bar = 10µm.

Given that the putative ABC3A (LmjF.11.1240; which corresponds to LmxM.11.1240 in *L. mexicana*) was totally absent from the entire *Lmj*LCB2-/- population, this member of a family of transporters implicated in the transport of lipids and in sterol homeostasis (Pasello et al., 2020) was considered of the greatest interest with regard to it possibly being part of a compensatory process necessary to enable knockout of the LCB2 gene. Using the *L. mexicana* CRISPR Cas9 system, the single copy gene encoding the putative ABC3A was readily deleted in *L. mexicana*, indicating that, as in *L. major*, it was not essential (**Figure 4** **)**. However, *Lmx*LCB2 (LmxM.34.0320) knockout failed using the same approach, contrasting with the ability to knock the gene out in *L. major* using homologous recombination (Zhang et al., 2003; Denny et al., 2004). A *Lmx*LCB2-/- population could not be selected despite multiple attempts, including using two selectable markers (for basticidin and G418) where no live cells were recovered. This finding led us to explore the possibility that the loss of ABC3A, as seen in the genomic analyses above, may have facilitated the loss of *Lmj*LCB2 by homologous recombination. Supporting this hypothesis, simultaneous knockout of *Lmx*LCB2 (LmxM.34.0320) and the putative ABC3A (LmxM.11.1240) in *L. mexicana* was readily achieved (**Figure 4**). Due to the absence of further available selective markers, the core five having already been utilized either in the parental line or the knockouts described here, add back lines could not be created in this study. However, preliminary phenotypic studies were undertaken. Both the putative ABC3A (LmxM.11.1240) knockout and the LmxM.11.1240/*Lmx*LCB2 (LmxM.34.0320) knockout lines grew at the same rate as the parental line and transformed into amastigote forms *in vitro* (data not shown). Interestingly, unlike the ABC3A knockout, the LmxM.11.1240/LmxM.34.0320 ablated line demonstrated the same distinct rounded morphology that was observed in the two independent *Lmj*LCB2 knockouts (Zhang et al., 2003; Denny et al., 2004) (**Figure 5**).

**Figure 6:**
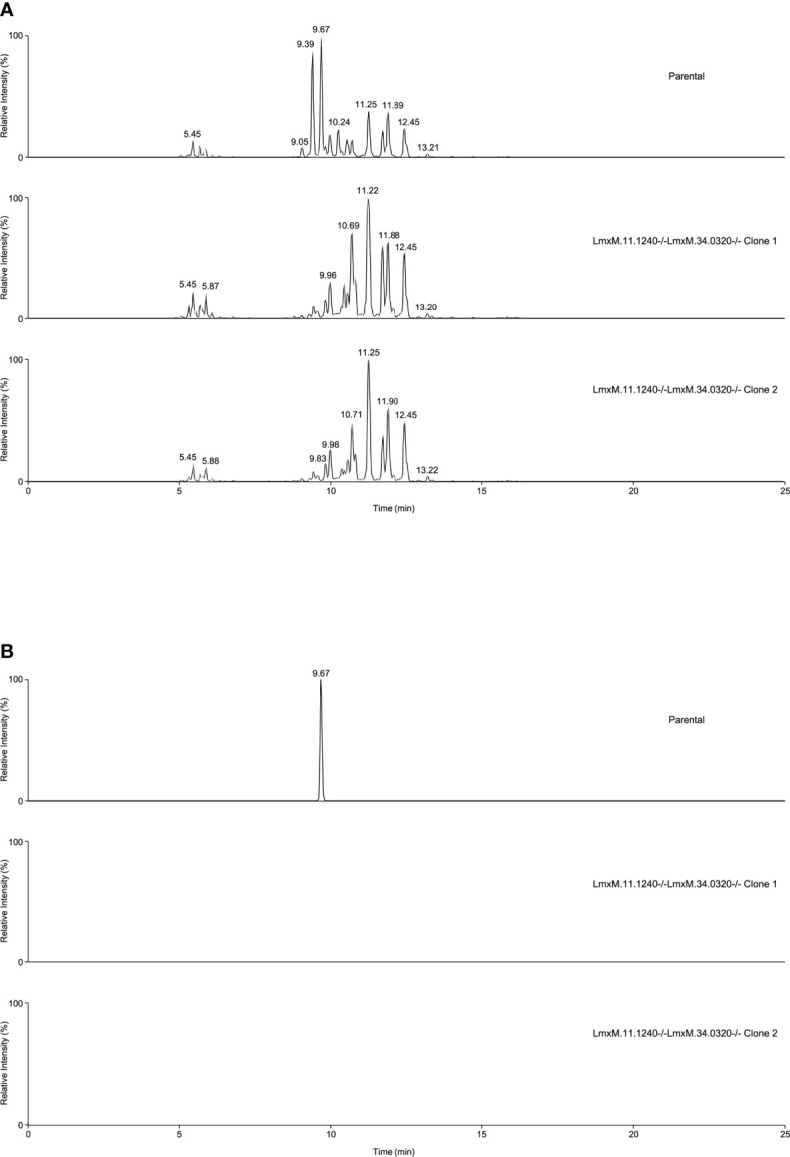
Global lipidomic analysis of LmxM.11.1240 and LmxM.34.0320 double knockout (LmxM.11.1240-/-LmxM.34.0320-/-) clones 1 and 2, and the parental line, *L. mexicana* strain T7 Cas9. Panel **A** shows exemplar negative ion mode LC-MS chromatograms of lipid profiles; Panel **B** shows extracted ion chromatograms attributable to the [M-H]^-^ ion of IPC 34:0:2 (m/z 780.5390).

**Figure 7:**
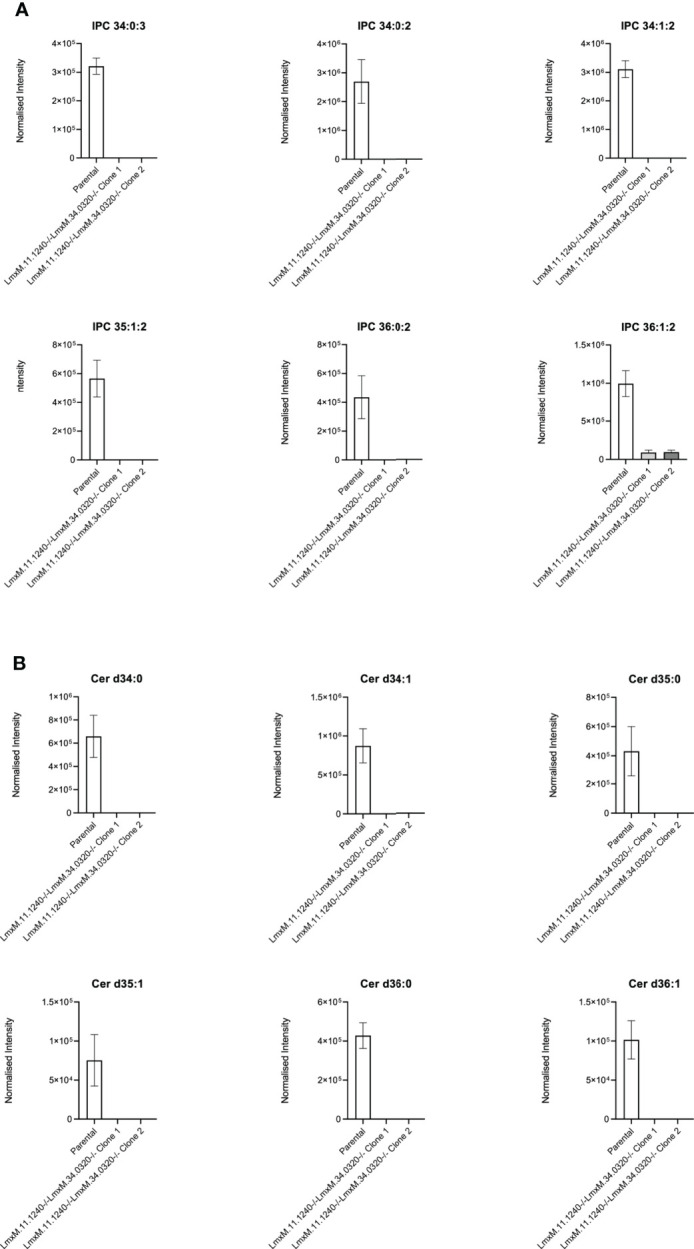
Ablation of IPC and ceramide molecular species in *L. mexicana*. Panel **A** shows IPC 34:0:3, IPC 34:0:2, IPC 34:1:2, 35:1:2, IPC 36:0:2 and IPC 36:1:2; Panel **B** shows Cer d34:0, d34:1, d35:0, d35:1, d36:0 and d36:1. The graphs reveal a substantial decrease in the intensity (mean ± SEM) of the sphingolipids in the LmxM.11.1240-/-LmxM.34.0320-/- mutants (n=5) compared to the parental line (n=3).

Further investigations of the LmxM.11.1240/LmxM.34.0320 combined knockout line was carried out by analyses of the lipidome using high resolution LC-MS (**Figures 6**, **7**). These data clearly demonstrated that IPC (**Figure 6** **[** **Figure 6B**: extracted ion chromatogram of IPC 34:0:2] and **Figure 7A**) and ceramide synthesis (**Figure 7B**) were ablated, as expected through the loss of SPT functionality, reflecting a specific loss of sphingolipid biosynthesis that mirrors previous findings in the *L. major Lmj*LCB2-/- lines (Zhang et al., 2003; Denny et al., 2004; Mina et al., 2021).

## Discussion

With the necessity of drug discovery for the Neglected Tropical Disease leishmaniasis as urgent as ever, the need to identify essential genes and processes which can be targeted by novel drug-like compounds is of the highest priority (Norcliffe et al., 2014; Denny and Steel, 2015; Denny, 2018a; Denny, 2018b). Until the recent advent of CRISPR Cas9 gene editing, this largely relied upon gene knockout using homologous recombination (Cruz and Beverley, 1990), involving two lengthy rounds of drug selection in these diploid organisms. Genomes of the *Leishmania* spp are now recognized to display extensive plasticity, especially during protracted cultivation (Rogers et al., 2011; Sterkers et al., 2011; Dumetz et al., 2017). This is reflected in frequent failures to create knockouts of genes where cells would duplicate specific chromosomes (or parts of those chromosomes) containing the gene of interest (Jones et al., 2018). However, the majority of genes studied [approximately 70% (Jones et al., 2018)] were deleted relatively easily using homologous recombination and selectable marker technology. The gene encoding the catalytic subunit of the first and rate limiting step in sphingolipid biosynthesis, serine palmitoyltransferase (SPT) (LCB2; LmjF.35.0320) was among those knocked out with relative ease in spite of the enzyme being essential in mammals (Hanada et al., 1998), yeast (Nagiec et al., 1994), plants (Chen et al., 2006) and other trypanosomatids (Fridberg et al., 2008). Surprisingly, the *L. major* LCB2 knockout (*Lmj*LCB2-/-) remained not just viable in culture but also infective in a murine model (Zhang et al., 2003; Denny and Smith, 2004; Denny et al., 2004). This result led to downgrading the priority afforded to what had been considered a promising drug target.

To investigate this knockout line further here we employed WGS to facilitate analyses of both the mutant and its equivalently cultured wild type (FV1) parent, technology unavailable when this line of created and analyzed in 2004. The study confirmed that both alleles of *Lmj*LCB2 (LmjF.35.0320) were completely deleted and replaced by hygromycin and puromycin selectable markers (Denny et al., 2004). However, multiple unplanned changes to the genome of the knockout line were also apparent. Notably, 9.8% of the 358 SNPs found in the *Lmj*LCB2-/- genome were protein altering (**Table 3** **).** More striking still, several non-targeted gene deletions specific to *Lmj*LCB2-/- were noted (**Figure 2**, **S5 Table**). One these encoded an amastin-like protein (LmjF.08.0740), a surface marker of pathogenic amastigote forms which is part of a large multigene family. This group of proteins have no obvious role in lipid biosynthesis, transport or any other function that could be hypothesized to compensate for the loss of sphingolipids. Furthermore, recent studies in *L. major* and in *L. donovani* combined high throughput long- and short-read sequencing platforms and found lower coverage in chromosome 8. A detailed analysis at the gene level showed that the lack of coverage in this region led to mis-annotation of an extensive region (~323 kb), including 6 out of the 11 amastin-like genes (LmjF.08.0670 to 0770) (Lypaczewski et al., 2018; Camacho et al., 2021). This status and the poor read quality in this region (**S1 Figure**) lead us to discount this as an important factor.

A region of substantially reduced read depth covering LmjF.13.1530 (encoding the miltefosine transporter; MT) and an adjacent gene of unknown function (LmjF.13.1540) was less ambiguous. However, despite good read quality and a clear loss of these genes, the CNV in this locus (**Figure 3B**, **Table 4**, **S5** **)** indicated incomplete ablation of this locus, apparently indicative of a mixed population in which a larger subpopulation had a deletion while a small subpopulation had not. The conclusion from these data was that although loss of these genes may confer a fitness advantage in the *L. major Lmj*LCB2-/- line, potentially in the murine host and relating to the natural function of MT as a phospholipid transporter (Perez-Victoria et al., 2006), the absence of this region was not essential for the deletion of the gene encoding the SPT catalytic subunit.

A further gene, this one encoding the putative ABC protein subfamily A, member 3 (ABCA3; LmjF.11.1240) was, however, completely and specifically lost in the *Lmj*LCB2-/- (**Figure 3A**). This led us to hypothesize that the deletion of *Lmj*LCB2 (LmjF.35.0320) was only made possible by the concurrent loss of LmjF.11.1240, thereby obscuring the evaluation of this *L. major* knockout line. This unstudied *Leishmania* protein is putatively a member of a family of transporters implicated in the transport of lipids and in sterol homeostasis (Pasello et al., 2020) and it is possible that its complete loss in the *Lmj*LCB2-/- line compensates for the lack of *de novo* sphingolipid biosynthesis by allowing membrane fluidity to be maintained without a need for endogenously synthesized sphingolipids.

Utilizing CRISPR Cas9 in the available *L. mexicana* system, the orthologue LmxM.11.1240 was readily ablated, with no obvious phenotype in terms of growth or morphology (**Figures 4**, **5**). However, in contrast, an equivalent attempt to knockout *Lmx*LCB2 (LmxM.35.0320) was unsuccessful, giving preliminary evidence that SPT function is essential in “unmodified” *L. mexicana.* Subsequently, in an attempt to test the hypothesis outlined above (that loss of LmjF.11.1240 could compensate for the targeted deletion of *Lmj*LCB2 [LmjF.35.0320]) when both genes were simultaneously targeted for ablation in *L. mexicana*. This was achieved with remarkable ease (**Figure 4**) with the morphology of the resultant mutants (**Figure 5**) reflecting that observed for original *L. major Lmj*LCB2-/- (Zhang et al., 2003; Denny et al., 2004). Furthermore, mass spectrometry demonstrated that IPC synthesis was undetectable in these cells (**Figure 6**), again reflecting the *L. major Lmj*LCB2-/- line (Zhang et al., 2003; Denny et al., 2004). This study indicated the importance of sequencing the whole genome of any manipulated *Leishmania* parasite to seek the presence of other untargeted changes that might emerge as necessary to change cellular physiology to compensate for the loss of otherwise essential genes. Furthermore, these findings impact on the view of several *Leishmania* spp enzymes, such as SPT, whose drug target status was degraded as a result of findings based on homologous recombination driven gene deletion (Jones et al., 2018). Re-evaluation of these is recommended.

## Data availability statement

The datasets presented in this study can be found in online repositories. The names of the repository/repositories and accession number(s) can be found below: https://www.ncbi.nlm.nih.gov/, PRJNA771089.

## Author contributions

EA-S was, with the support of WW, responsible for the analyses, interpretation and presentation of the genomic data of *L. major* lines. YK was responsible for the creation and analyses of the genetically modified cell lines. PW led, with the support of YK, the lipid analyses. MB had overall responsibility for the generation of sequence data. PD was the project lead and grant awardee. EA-S, MB and PD were responsible for the writing and editing of the manuscript. All authors contributed to the article and approved the submitted version.

## Funding

This work was supported by MRC Confidence in Concept MC-PC-17157 (PD); the UKRI - Global Challenges Research Fund, ‘A Global Network for Neglected Tropical Diseases’ MR/P027989/1 (PD; https://www.ukri.org); and an MRC Newton grant Bridging epigenetics, metabolism and cell cycle in pathogenic trypanosomatids, MR/S019650/1 (MB). The funders had no role in study design, data collection and analysis, decision to publish, or preparation of the manuscript.

## Acknowledgments

We would like to acknowledge Glasgow Polyomics for support in the acquisition of WGS and mass spectrometry (LC-MS) data, and Jon Wilkes (Wellcome Centre for Integrative Parasitology) and Graham Hamilton (Glasgow Polyomics) for technical support and input. This work also made use of the Durham University Hamilton HPC Service of for all WGS data analyses performed.

## Conflict of interest

The authors declare that the research was conducted in the absence of any commercial or financial relationships that could be construed as a potential conflict of interest.

## Publisher’s note

All claims expressed in this article are solely those of the authors and do not necessarily represent those of their affiliated organizations, or those of the publisher, the editors and the reviewers. Any product that may be evaluated in this article, or claim that may be made by its manufacturer, is not guaranteed or endorsed by the publisher.
