# Supplementary material for: Genome deletions to overcome the directed loss of gene function in Leishmania
Source: Front Cell Infect Microbiol. 2022 Sep 23;12:988688. doi: 10.3389/fcimb.2022.988688 (PMC9539739; doi:10.3389/fcimb.2022.988688)
Supplement: Supplementary file 1 [file DataSheet_1.pdf]

# *Supplementary Material*

S1 Figure. Visualisation of genome sequencing coverage of LmjF.08.0740 (red arrow) and amastin family (red and black circles) related present in Chromosome 8. The top part of each panel represents coverage (green), whereas the bottom part depicts individual reads. The vertical black lines denote separate (contiguous) panels. Reads with mapping quality of zero (white-filled bars) or between 40 and 60 (grey bars) in FV1 and LmjLCB2-/- cell lines. The blue line at the bottom of each panel indicates the gene (s) CDS (gene). WGS data were aligned to the reference genome (https://tritrypdb.org) and the image was produced using IGV_2.8.9 (Robinson et al., 2017).

S2 Figure. Alignment of LmjF.08.0740 and amastin gene family related present in Chromosome 8. Sequences are shown in their genomic order upstream to downstream. Phylogram showing three main groups based on their distance (panel A) and amino acids identity (panel B). Red box and shadowed boxes denote the same group in both panes. Sequences were retrieved from TriTrypDB (https://tritrypdb.org) and alignment was performed with Clustal Ω (https://www.ebi.ac.uk/Tools/msa/clustalo/) (Thompson et al., 1994).

S3 Figure. Alignment of LmjF.11.1220 and ABCA gene family related alongside with other ABCA genes present in chromosomes (02, 15, 27 and 29) other than 11. Sequences are shown in their genomic order upstream to downstream. Phylogram showing three main groups based on their distance (panel A) and amino acids identity (panel B). Red box and shadowed boxes denote the same group in both panes. Sequences were retrieved from TriTrypDB (https://tritrypdb.org) and alignment was performed with Clustal Ω (https://www.ebi.ac.uk/Tools/msa/clustalo/) (Thompson et al., 1994).

S4 Figure. Coverage of the miltefosine transporter of (MT) and adjacent genes in *L. mexicana*. The coordinates of a region spanning a genomic region of ~25 kb covering genes shown at the bottom (blue bars). Coverage is shown at the top of each panel in four amphotericin B resistant lines (red) alongside their parental wild type (green). In three resistant lines (AmBRB/cl.2, AmBRcl.8 and AmBRcl.6) a total lack of coverage ~9 kb comprises LmxM.13.1530 and adjacent gene downstream LmxM.13.1540 is shown while one line (AmBRA4) the total deleted region (19 kb) includes another two genes (LmxM.13.1510 and LmxM.13.1520). Small gaps of low coverage are or interspersed reads are observed in the intergenic regions in all lines including wild type, the latter in which the above mentioned genes are present. WGS data were aligned to the reference genome (https://tritrypdb.org) and the images were produced with IGV_2.8.9 software (Robinson et al., 2017) (http://software.broadinstitute.org/software/igv/).

S1 Table. General Statistics of Whole Genome Sequencing in *Lmj*LCB2-/- (and FV1). See Materials and Methods, section 2.2 for a full description of WGS analyses.

S2 Table. SNPs identified in *Lmj*LCB2-/- using WGS. See Material and Methods, section 2.2 for a full description of WGS analyses.

S3 Table. Protein altering indels found in *Lmj*LCB2-/-. See Materials and Methods, section 2.2 for a full description of WGS analyses.

S4 Table. Copy number variation (ploidy) per Chromosome found in *Lmj*LCB2-/- (and FV1). See Materials and Methods, section 2.2 for a full description of WGS analyses.

S5 Table. CNV (full list) and gene amplifications & deletions found in *Lmj*LCB2-/- (and FV1). See Materials and Methods, section 2.2 for a full description of WGS analyses.

S6 Table. BLASTN analysis of reads (n=30) from LmjF.11.1220 (ABCA3) in *Lmj*LCB2-/-.
