# Supplementary figures and images for: Genome deletions to overcome the directed loss of gene function in Leishmania
Source: Front Cell Infect Microbiol. 2022 Sep 23;12:988688. doi: 10.3389/fcimb.2022.988688 (PMC9539739; doi:10.3389/fcimb.2022.988688)

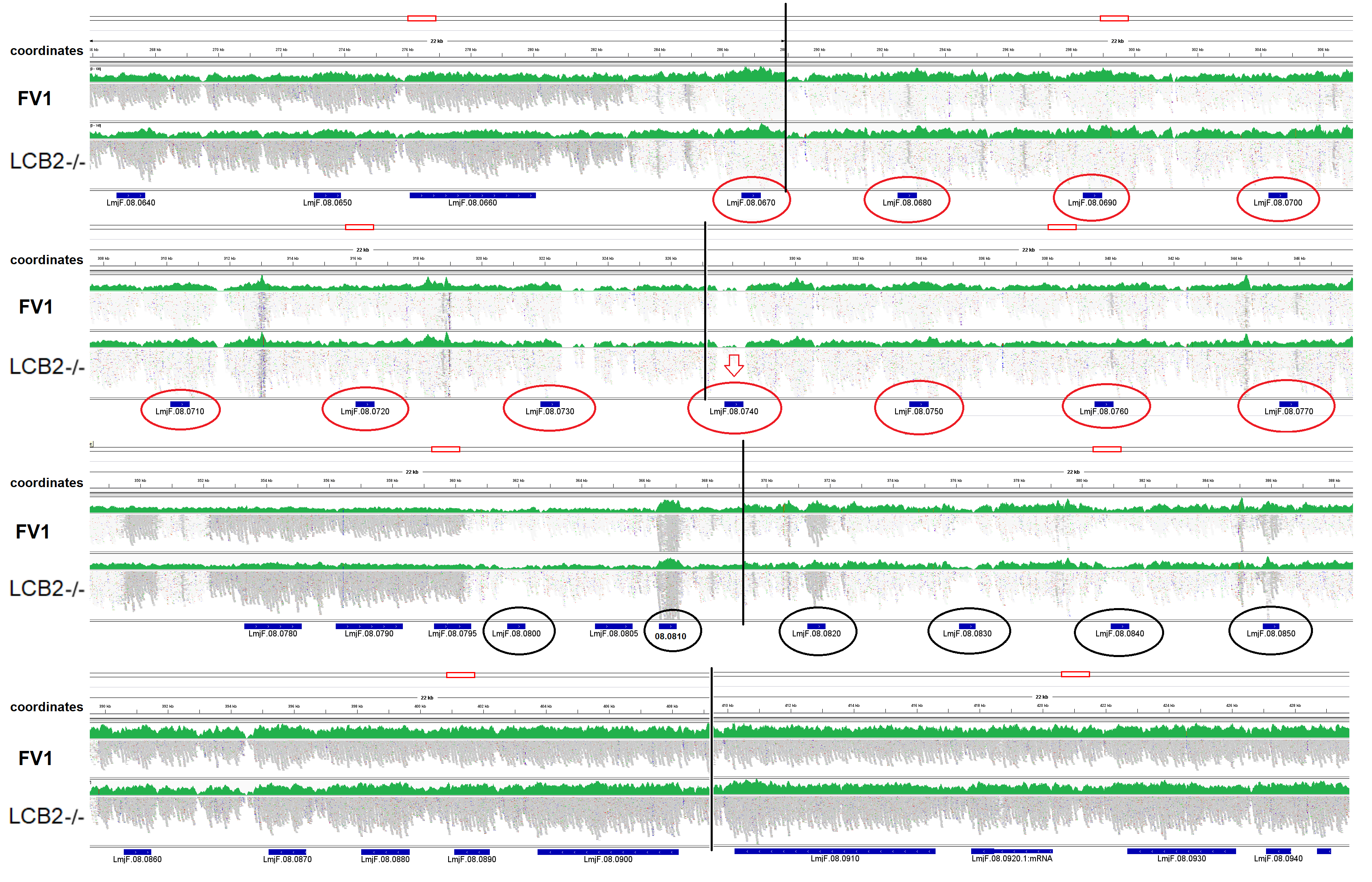

Supplement: Supplementary file 2 [file Image_1.tiff]

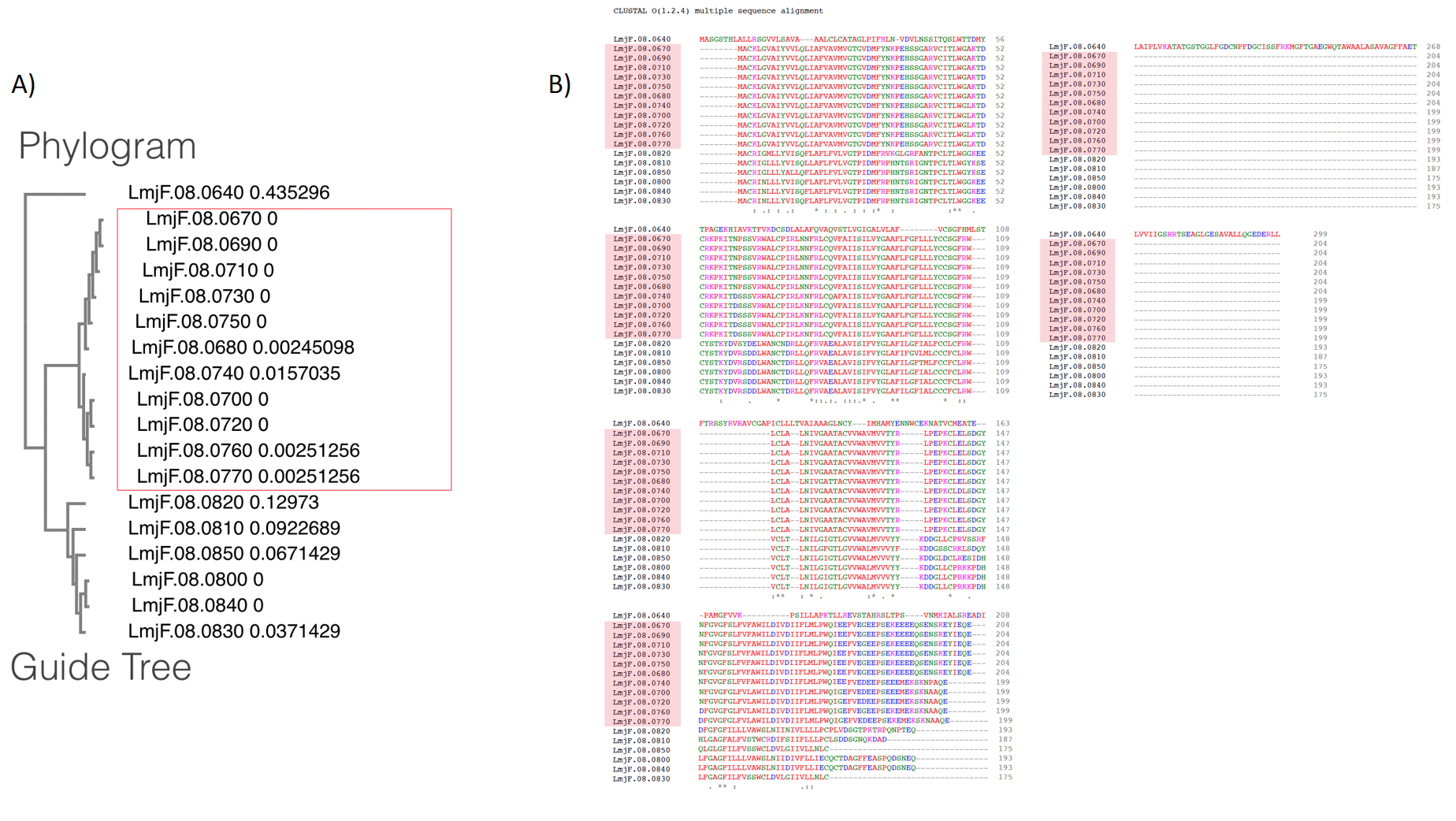

Supplement: Supplementary file 3 [file Image_2.tiff]

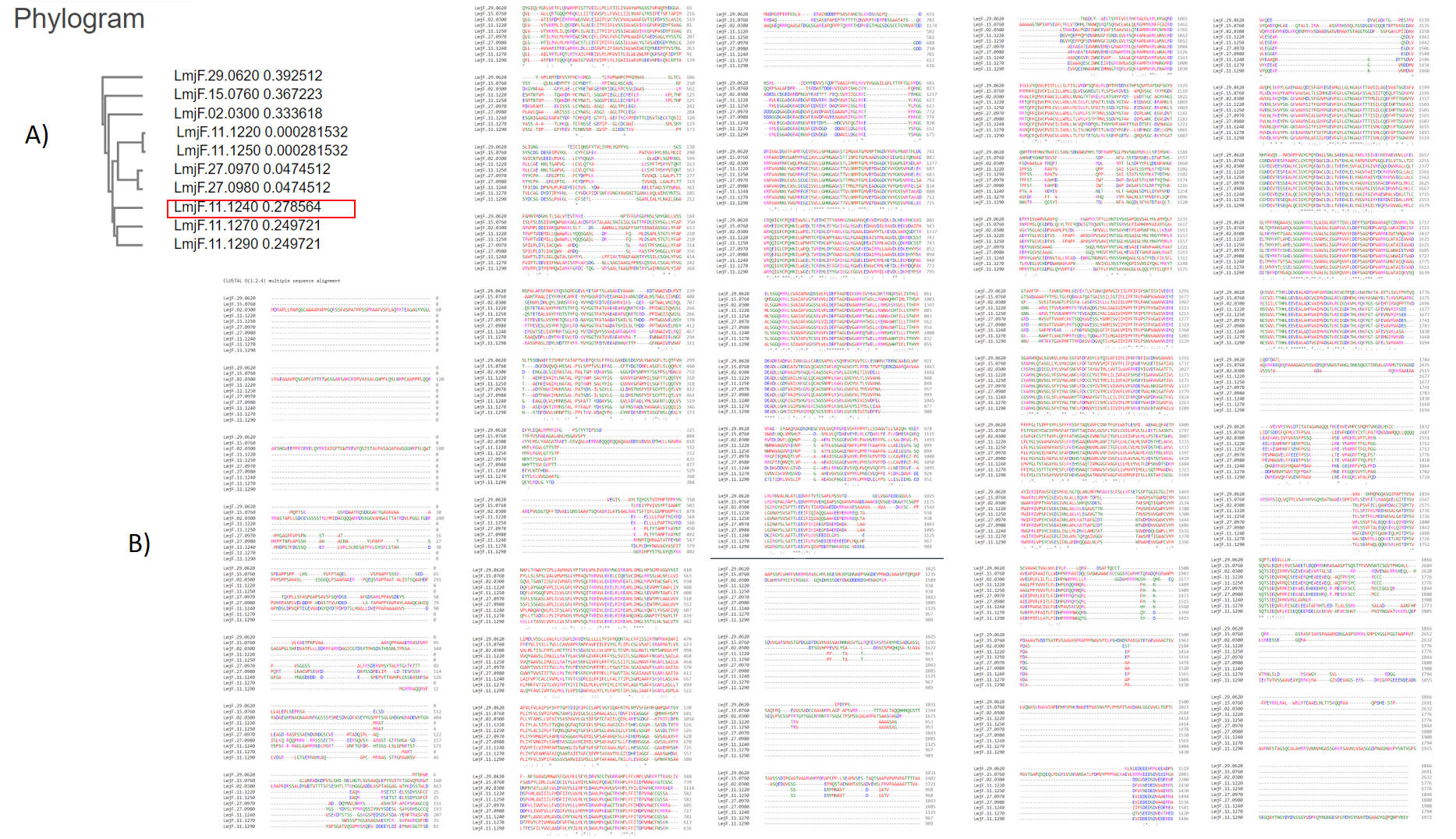

Supplement: Supplementary file 4 [file Image_3.tiff]

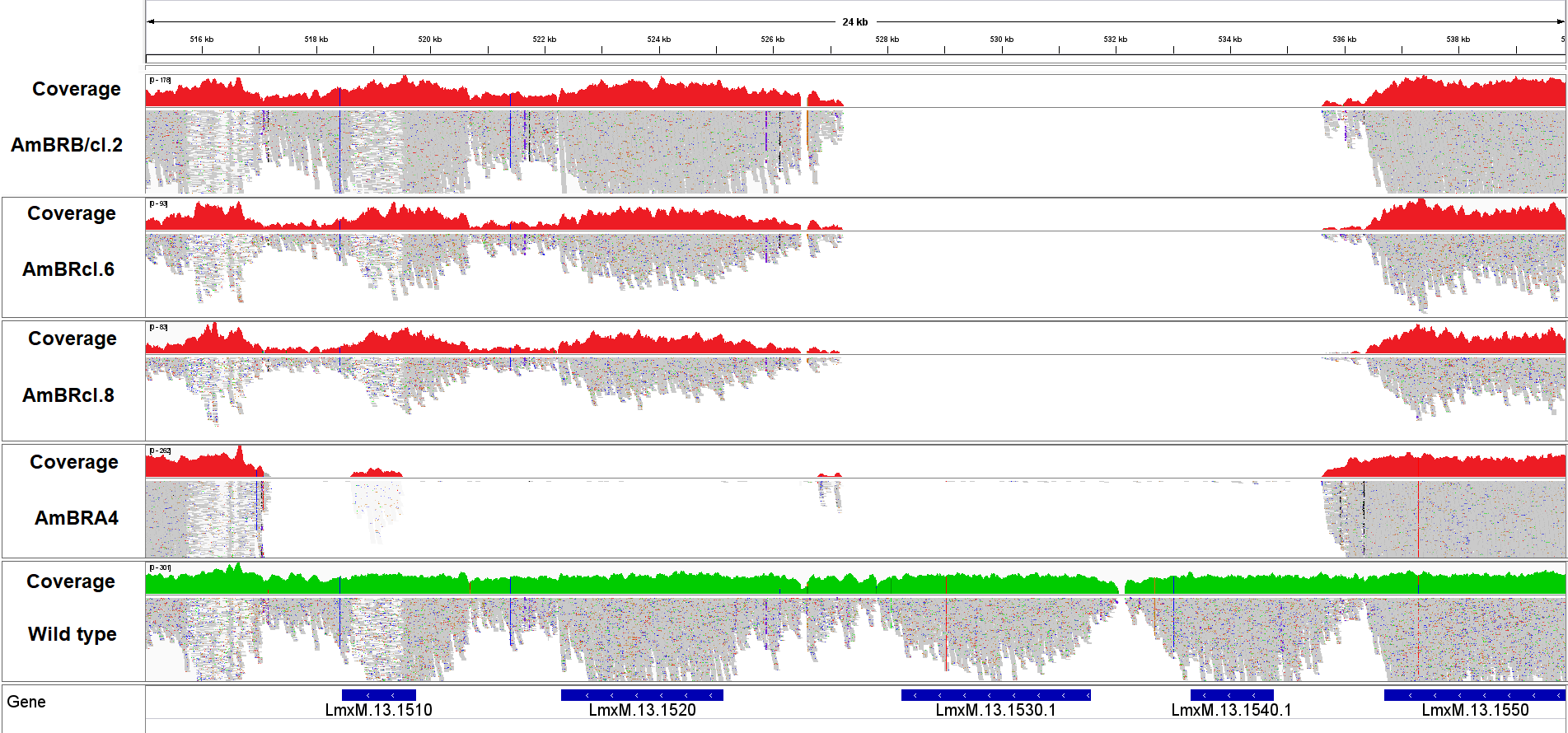

Supplement: Supplementary file 5 [file Image_4.tiff]
