# Supplementary material for: Genome deletions to overcome the directed loss of gene function in Leishmania
Source: Front Cell Infect Microbiol. 2022 Sep 23;12:988688. doi: 10.3389/fcimb.2022.988688 (PMC9539739; doi:10.3389/fcimb.2022.988688)
Supplement: Supplementary file 11 [file Table_6.docx]

Random reads (n=20) selected from LCB2(BAM file) with MQ=0 which are mis-mapped to LmjF.11.1240 but possibly belong to either LmjF.11.1220 to 1290

| List of Reads | Sequence |
| --- | --- |
| >1 | CTCTGTGCTGCTGGGGCACAACGGCGCCGGCAAGACGACGGTGCTGAACATGATGACGGGGATGGTCGAGCCGG |
| >2 | GAACAACCTGTACTGGGGGATGCGCGAGGGCGAGATCTCTGTGCTGCTGGGGCACAACGGCGCCGGCAAGACGAC |
| >3 | GTCGTGCATTGATCGGAACGTGACGGCGCTGCTGAGATTTGCGGATGAGTGGCGCGGTACCAACGTGAACGTGT |
| >4 | GCATTGATCGGAACGTGACGGCGCTGCTGAGATTTGCGGATGAGTGGCGCGGTACCAACGTGAACGTGTTCCTGT |
| >5 | GCGACTGCTACGTCTACGGCAGCTCTGTGCGGACGGCAAAGGCGGACGTGCGGCAGCAGATCGGGTACTGCCCGC |
| >6 | GCACGACATGCCGCGCTACGGCGCCGTGTCGTGCATTGATCGGAACGTGACGGCGCTGCTGAGATTTGCGGATG |
| >7 | GGATGGTCGAGCCGGACGCGGGCGACTGCTACGTCTACGGCAGCTCTGTGCGGACGGCAAAGGCGGACGTGCGGC |
| >8 | GCAGGCGCTGTCGGACTACCTGCTGGATACATGGTACGTGCACGACATGCCGCGCTACGGCGCCGTGTCGTGCAT |
| >9 | CGAGGTGAGCGTGCGGCTGGCGTCGGCGCAGCACGCGGAGCGCTTTGCGTCGTTCATGCAGGCGGCGTTCCCCG |
| >10 | GCGGCTGGCGTCGGCGCAGCACGCGGAGCGCTTTGCGTCGTTCATGCAGGCGGCGTTCCCCGACGCTGTGCGGA |
|  |  |
| >11 | GGAGGAGGGGGAGGCGCTTGCGGACATTGTGGCGATCATGGTGCGCGGGTACGTGCGGTGTGTCGGCGACAAG |
| >12 | AGAACAAGTTCGGCAGCGCCTTCGAGGTGAGCGTGCGGCTGGCGTCGGCGCAGCACGCGGAGCGCTTTGCGTCGT |
| >13 | TGGCCTACGAGGTAAACGAGACTCACTTGACGGGTGCGCAGGCGCTGTCGGACTACCTGCTGGATACATGGTACG |
| >14 | GCAAGAGGTTCGTTGCGGTGAACAACCTGTACTGGGGGATGCGCGAGGGCGAGATCTCTGTGCTGCTGGGGCAC |
| >15 | CTCTGTGCTGCTGGGGCACAACGGCGCCGGCAAGACGACGGTGCTGAACATGATGACGGGGATGGTCGAGCCGG |
| >16 | GGCGCGGTACCAACGTGAACGTGTTCCTGTACAACTCCTCCTCGCACCACCAGGCTGGGCTGAGCCTCGCAACGT |
| >17 | CTCCGTTGTGCTGACGACGCACCACCTGGAGGAGGTGGAGGCGCTTGCGGACATTGTGGCGATCATGGTGCGCGG |
| >18 | ACCTGAAGAACAAGTTCGGCAGCGCCTTCGAGGTGAGCGTGCGGCTGGCGTCGGCGCAGCACGCGGAGCGCTTTG |
| >19 | ACCTGAAGAACAAGTTCGGCAGCGCCTTCGAGGTGAGCGTGCGGCTGGCGTCGGCGCAGCACGCGGAGCGCTTTG |
| >20 | CACCTGGAGGAGGTGGAGGCGCTTGCGGACATTGTGGCGATCATGGTGCGCGGGTACGTGCGGTGTGTCGGCGAC |
|  |  |

BLASTN 2.10.1+

Reference: Stephen F. Altschul, Thomas L. Madden, Alejandro A.

Schaffer, Jinghui Zhang, Zheng Zhang, Webb Miller, and David J.

Lipman (1997), "Gapped BLAST and PSI-BLAST: a new generation of

protein database search programs", Nucleic Acids Res. 25:3389-3402.

Database: /eupath/data/apiSiteFilesStaging/TriTrypDB/52/real/webServices/TriTr

ypDB/release-

CURRENT/LmajorFriedlin/blast/LmajorFriedlinAnnotatedTranscripts

9,495 sequences; 16,705,993 total letters

Query= 1

Length=74

Score E

Sequences producing significant alignments: (Bits) Value

LmjF.11.1290:mRNA | gene=LmjF.11.1290 | organism=Leishmania_major... 134 2e-32

LmjF.11.1270:mRNA | gene=LmjF.11.1270 | organism=Leishmania_major... 134 2e-32

LmjF.11.1250:mRNA | gene=LmjF.11.1250 | organism=Leishmania_major... 134 2e-32

LmjF.11.1240:mRNA | gene=LmjF.11.1240 | organism=Leishmania_major... 134 2e-32

LmjF.11.1220:mRNA | gene=LmjF.11.1220 | organism=Leishmania_major... 134 2e-32

LmjF.02.0300:mRNA | gene=LmjF.02.0300 | organism=Leishmania_major... 94.2 7e-20

LmjF.27.0980:mRNA | gene=LmjF.27.0980 | organism=Leishmania_major... 82.4 1e-16

LmjF.27.0970:mRNA | gene=LmjF.27.0970 | organism=Leishmania_major... 82.4 1e-16

LmjF.29.0620:mRNA | gene=LmjF.29.0620 | organism=Leishmania_major... 58.1 5e-09

LmjF.30.1330:mRNA | gene=LmjF.30.1330 | organism=Leishmania_major... 38.3 0.005

LmjF.34.4280:mRNA | gene=LmjF.34.4280 | organism=Leishmania_major... 35.6 0.017

LmjF.32.2810:mRNA | gene=LmjF.32.2810 | organism=Leishmania_major... 35.6 0.017

LmjF.33.1860:mRNA | gene=LmjF.33.1860 | organism=Leishmania_major... 33.7 0.058

LmjF.12.0610:mRNA | gene=LmjF.12.0610 | organism=Leishmania_major... 33.7 0.058

LmjF.06.0090:mRNA | gene=LmjF.06.0090 | organism=Leishmania_major... 33.7 0.058

LmjF.06.0080:mRNA | gene=LmjF.06.0080 | organism=Leishmania_major... 33.7 0.058

LmjF.05.0910:mRNA | gene=LmjF.05.0910 | organism=Leishmania_major... 32.8 0.20

LmjF.28.0220:mRNA | gene=LmjF.28.0220 | organism=Leishmania_major... 31.9 0.20

LmjF.21.1210:mRNA | gene=LmjF.21.1210 | organism=Leishmania_major... 31.9 0.20

LmjF.14.1060:mRNA | gene=LmjF.14.1060 | organism=Leishmania_major... 31.9 0.20

LmjF.08.0430:mRNA | gene=LmjF.08.0430 | organism=Leishmania_major... 31.9 0.20

LmjF.36.6030:mRNA | gene=LmjF.36.6030 | organism=Leishmania_major... 30.1 0.71

LmjF.34.0310:mRNA | gene=LmjF.34.0310 | organism=Leishmania_major... 30.1 0.71

LmjF.33.2610:mRNA | gene=LmjF.33.2610 | organism=Leishmania_major... 30.1 0.71

LmjF.32.3870:mRNA | gene=LmjF.32.3870 | organism=Leishmania_major... 30.1 0.71

LmjF.32.1110:mRNA | gene=LmjF.32.1110 | organism=Leishmania_major... 30.1 0.71

LmjF.30.2210:mRNA | gene=LmjF.30.2210 | organism=Leishmania_major... 30.1 0.71

LmjF.16.0980:mRNA | gene=LmjF.16.0980 | organism=Leishmania_major... 30.1 0.71

LmjF.36.3170:mRNA | gene=LmjF.36.3170 | organism=Leishmania_major... 29.2 2.5

LmjF.27.1750:mRNA | gene=LmjF.27.1750 | organism=Leishmania_major... 29.2 2.5

LmjF.22.1240:mRNA | gene=LmjF.22.1240 | organism=Leishmania_major... 29.2 2.5

LmjF.21.0680:mRNA | gene=LmjF.21.0680 | organism=Leishmania_major... 29.2 2.5

LmjF.16.0370:mRNA | gene=LmjF.16.0370 | organism=Leishmania_major... 29.2 2.5

LmjF.14.1490:mRNA | gene=LmjF.14.1490 | organism=Leishmania_major... 29.2 2.5

LmjF.11.0320:mRNA | gene=LmjF.11.0320 | organism=Leishmania_major... 29.2 2.5

LmjF.04.0600:mRNA | gene=LmjF.04.0600 | organism=Leishmania_major... 29.2 2.5

LmjF.36.3530:mRNA | gene=LmjF.36.3530 | organism=Leishmania_major... 28.3 2.5

LmjF.34.1000:mRNA | gene=LmjF.34.1000 | organism=Leishmania_major... 28.3 2.5

LmjF.33.2940:mRNA | gene=LmjF.33.2940 | organism=Leishmania_major... 28.3 2.5

LmjF.32.3450:mRNA | gene=LmjF.32.3450 | organism=Leishmania_major... 28.3 2.5

LmjF.32.2150:mRNA | gene=LmjF.32.2150 | organism=Leishmania_major... 28.3 2.5

LmjF.32.0260:mRNA | gene=LmjF.32.0260 | organism=Leishmania_major... 28.3 2.5

LmjF.28.0940:mRNA | gene=LmjF.28.0940 | organism=Leishmania_major... 28.3 2.5

LmjF.25.1110:mRNA | gene=LmjF.25.1110 | organism=Leishmania_major... 28.3 2.5

LmjF.20.1190:mRNA | gene=LmjF.20.1190 | organism=Leishmania_major... 28.3 2.5

LmjF.19.1050:mRNA | gene=LmjF.19.1050 | organism=Leishmania_major... 28.3 2.5

LmjF.16.1190:mRNA | gene=LmjF.16.1190 | organism=Leishmania_major... 28.3 2.5

LmjF.09.0070:mRNA | gene=LmjF.09.0070 | organism=Leishmania_major... 28.3 2.5

LmjF.02.0140:mRNA | gene=LmjF.02.0140 | organism=Leishmania_major... 28.3 2.5

LmjF.01.0670:mRNA | gene=LmjF.01.0670 | organism=Leishmania_major... 28.3 2.5

LmjF.36.4290:mRNA | gene=LmjF.36.4290 | organism=Leishmania_major... 27.4 8.7

LmjF.34.3610:mRNA | gene=LmjF.34.3610 | organism=Leishmania_major... 27.4 8.7

LmjF.32.3740:mRNA | gene=LmjF.32.3740 | organism=Leishmania_major... 27.4 8.7

LmjF.28.1880:mRNA | gene=LmjF.28.1880 | organism=Leishmania_major... 27.4 8.7

LmjF.28.1460:mRNA | gene=LmjF.28.1460 | organism=Leishmania_major... 27.4 8.7

LmjF.26.2410:mRNA | gene=LmjF.26.2410 | organism=Leishmania_major... 27.4 8.7

LmjF.25.0770:mRNA | gene=LmjF.25.0770 | organism=Leishmania_major... 27.4 8.7

LmjF.25.0530:mRNA | gene=LmjF.25.0530 | organism=Leishmania_major... 27.4 8.7

LmjF.24.1900:mRNA | gene=LmjF.24.1900 | organism=Leishmania_major... 27.4 8.7

LmjF.24.0660:mRNA | gene=LmjF.24.0660 | organism=Leishmania_major... 27.4 8.7

LmjF.23.1270:mRNA | gene=LmjF.23.1270 | organism=Leishmania_major... 27.4 8.7

LmjF.23.0830:mRNA | gene=LmjF.23.0830 | organism=Leishmania_major... 27.4 8.7

LmjF.16.1340:mRNA | gene=LmjF.16.1340 | organism=Leishmania_major... 27.4 8.7

LmjF.15.1330:mRNA | gene=LmjF.15.1330 | organism=Leishmania_major... 27.4 8.7

LmjF.36.4150:mRNA | gene=LmjF.36.4150 | organism=Leishmania_major... 26.5 8.7

LmjF.36.2880:mRNA | gene=LmjF.36.2880 | organism=Leishmania_major... 26.5 8.7

LmjF.35.4910:mRNA | gene=LmjF.35.4910 | organism=Leishmania_major... 26.5 8.7

LmjF.35.2950:mRNA | gene=LmjF.35.2950 | organism=Leishmania_major... 26.5 8.7

LmjF.34.2980:mRNA | gene=LmjF.34.2980 | organism=Leishmania_major... 26.5 8.7

LmjF.34.1540:mRNA | gene=LmjF.34.1540 | organism=Leishmania_major... 26.5 8.7

LmjF.33.2590:mRNA | gene=LmjF.33.2590 | organism=Leishmania_major... 26.5 8.7

LmjF.33.2180:mRNA | gene=LmjF.33.2180 | organism=Leishmania_major... 26.5 8.7

LmjF.33.2170:mRNA | gene=LmjF.33.2170 | organism=Leishmania_major... 26.5 8.7

LmjF.33.1090:mRNA | gene=LmjF.33.1090 | organism=Leishmania_major... 26.5 8.7

LmjF.32.3930:mRNA | gene=LmjF.32.3930 | organism=Leishmania_major... 26.5 8.7

LmjF.32.2800:mRNA | gene=LmjF.32.2800 | organism=Leishmania_major... 26.5 8.7

LmjF.32.1720:mRNA | gene=LmjF.32.1720 | organism=Leishmania_major... 26.5 8.7

LmjF.32.0190:mRNA | gene=LmjF.32.0190 | organism=Leishmania_major... 26.5 8.7

LmjF.31.1720:mRNA | gene=LmjF.31.1720 | organism=Leishmania_major... 26.5 8.7

LmjF.31.0500:mRNA | gene=LmjF.31.0500 | organism=Leishmania_major... 26.5 8.7

LmjF.30.1810:mRNA | gene=LmjF.30.1810 | organism=Leishmania_major... 26.5 8.7

LmjF.29.2240:mRNA | gene=LmjF.29.2240 | organism=Leishmania_major... 26.5 8.7

LmjF.28.2580:mRNA | gene=LmjF.28.2580 | organism=Leishmania_major... 26.5 8.7

LmjF.27.2590:mRNA | gene=LmjF.27.2590 | organism=Leishmania_major... 26.5 8.7

LmjF.26.1900:mRNA | gene=LmjF.26.1900 | organism=Leishmania_major... 26.5 8.7

LmjF.26.0720:mRNA | gene=LmjF.26.0720 | organism=Leishmania_major... 26.5 8.7

LmjF.26.0550:mRNA | gene=LmjF.26.0550 | organism=Leishmania_major... 26.5 8.7

LmjF.25.1950:mRNA | gene=LmjF.25.1950 | organism=Leishmania_major... 26.5 8.7

LmjF.25.0980:mRNA | gene=LmjF.25.0980 | organism=Leishmania_major... 26.5 8.7

LmjF.25.0830:mRNA | gene=LmjF.25.0830 | organism=Leishmania_major... 26.5 8.7

LmjF.23.0543:mRNA | gene=LmjF.23.0543 | organism=Leishmania_major... 26.5 8.7

LmjF.23.0440:mRNA | gene=LmjF.23.0440 | organism=Leishmania_major... 26.5 8.7

LmjF.22.1620:mRNA | gene=LmjF.22.1620 | organism=Leishmania_major... 26.5 8.7

LmjF.21.1340:mRNA | gene=LmjF.21.1340 | organism=Leishmania_major... 26.5 8.7

LmjF.20.1530:mRNA | gene=LmjF.20.1530 | organism=Leishmania_major... 26.5 8.7

LmjF.15.1320:mRNA | gene=LmjF.15.1320 | organism=Leishmania_major... 26.5 8.7

LmjF.15.0595:pseudogenic_transcript | gene=LmjF.15.0595 | organis... 26.5 8.7

LmjF.12.0640:mRNA | gene=LmjF.12.0640 | organism=Leishmania_major... 26.5 8.7

LmjF.09.0390:mRNA | gene=LmjF.09.0390 | organism=Leishmania_major... 26.5 8.7

LmjF.08.1080:mRNA | gene=LmjF.08.1080 | organism=Leishmania_major... 26.5 8.7

>LmjF.11.1290:mRNA | gene=LmjF.11.1290 | organism=Leishmania_major_strain_Friedlin

| gene_product=ATP-binding cassette protein

subfamily A, member 6, putative | transcript_product=ATP-binding

cassette protein subfamily A, member 6, putative |

location=LmjF.11:545392-551310(+) | length=5919 | sequence_SO=chromosome

| SO=protein_coding_gene | is_pseudo=false

Length=5919

Score = 134 bits (148), Expect = 2e-32

Identities = 74/74 (100%), Gaps = 0/74 (0%)

Strand=Plus/Plus

Query 1 CTCTGTGCTGCTGGGGCACAACGGCGCCGGCAAGACGACGGTGCTGAACATGATGACGGG 60

||||||||||||||||||||||||||||||||||||||||||||||||||||||||||||

Sbjct 2079 CTCTGTGCTGCTGGGGCACAACGGCGCCGGCAAGACGACGGTGCTGAACATGATGACGGG 2138

Query 61 GATGGTCGAGCCGG 74

||||||||||||||

Sbjct 2139 GATGGTCGAGCCGG 2152

Score = 26.5 bits (28), Expect = 8.7

Identities = 14/14 (100%), Gaps = 0/14 (0%)

Strand=Plus/Plus

Query 8 CTGCTGGGGCACAA 21

||||||||||||||

Sbjct 2578 CTGCTGGGGCACAA 2591

>LmjF.11.1270:mRNA | gene=LmjF.11.1270 | organism=Leishmania_major_strain_Friedlin

| gene_product=ATP-binding cassette protein

subfamily A, member 5, putative | transcript_product=ATP-binding

cassette protein subfamily A, member 5, putative |

location=LmjF.11:531133-536517(+) | length=5385 | sequence_SO=chromosome

| SO=protein_coding_gene | is_pseudo=false

Length=5385

Score = 134 bits (148), Expect = 2e-32

Identities = 74/74 (100%), Gaps = 0/74 (0%)

Strand=Plus/Plus

Query 1 CTCTGTGCTGCTGGGGCACAACGGCGCCGGCAAGACGACGGTGCTGAACATGATGACGGG 60

||||||||||||||||||||||||||||||||||||||||||||||||||||||||||||

Sbjct 2010 CTCTGTGCTGCTGGGGCACAACGGCGCCGGCAAGACGACGGTGCTGAACATGATGACGGG 2069

Query 61 GATGGTCGAGCCGG 74

||||||||||||||

Sbjct 2070 GATGGTCGAGCCGG 2083

Score = 26.5 bits (28), Expect = 8.7

Identities = 14/14 (100%), Gaps = 0/14 (0%)

Strand=Plus/Plus

Query 8 CTGCTGGGGCACAA 21

||||||||||||||

Sbjct 2509 CTGCTGGGGCACAA 2522

>LmjF.11.1250:mRNA | gene=LmjF.11.1250 | organism=Leishmania_major_strain_Friedlin

| gene_product=ATP-binding cassette protein

subfamily A, member 4, putative | transcript_product=ATP-binding

cassette protein subfamily A, member 4, putative |

location=LmjF.11:520967-526297(+) | length=5331 | sequence_SO=chromosome

| SO=protein_coding_gene | is_pseudo=false

Length=5331

Score = 134 bits (148), Expect = 2e-32

Identities = 74/74 (100%), Gaps = 0/74 (0%)

Strand=Plus/Plus

Query 1 CTCTGTGCTGCTGGGGCACAACGGCGCCGGCAAGACGACGGTGCTGAACATGATGACGGG 60

||||||||||||||||||||||||||||||||||||||||||||||||||||||||||||

Sbjct 1923 CTCTGTGCTGCTGGGGCACAACGGCGCCGGCAAGACGACGGTGCTGAACATGATGACGGG 1982

Query 61 GATGGTCGAGCCGG 74

||||||||||||||

Sbjct 1983 GATGGTCGAGCCGG 1996

>LmjF.11.1240:mRNA | gene=LmjF.11.1240 | organism=Leishmania_major_strain_Friedlin

| gene_product=ATP-binding cassette protein

subfamily A, member 3, putative | transcript_product=ATP-binding

cassette protein subfamily A, member 3, putative |

location=LmjF.11:510352-516078(+) | length=5727 | sequence_SO=chromosome

| SO=protein_coding_gene | is_pseudo=false

Length=5727

Score = 134 bits (148), Expect = 2e-32

Identities = 74/74 (100%), Gaps = 0/74 (0%)

Strand=Plus/Plus

Query 1 CTCTGTGCTGCTGGGGCACAACGGCGCCGGCAAGACGACGGTGCTGAACATGATGACGGG 60

||||||||||||||||||||||||||||||||||||||||||||||||||||||||||||

Sbjct 2514 CTCTGTGCTGCTGGGGCACAACGGCGCCGGCAAGACGACGGTGCTGAACATGATGACGGG 2573

Query 61 GATGGTCGAGCCGG 74

||||||||||||||

Sbjct 2574 GATGGTCGAGCCGG 2587

>LmjF.11.1220:mRNA | gene=LmjF.11.1220 | organism=Leishmania_major_strain_Friedlin

| gene_product=ATP-binding cassette protein

subfamily A, member 2, putative | transcript_product=ATP-binding

cassette protein subfamily A, member 2, putative |

location=LmjF.11:500104-505434(+) | length=5331 | sequence_SO=chromosome

| SO=protein_coding_gene | is_pseudo=false

Length=5331

Score = 134 bits (148), Expect = 2e-32

Identities = 74/74 (100%), Gaps = 0/74 (0%)

Strand=Plus/Plus

Query 1 CTCTGTGCTGCTGGGGCACAACGGCGCCGGCAAGACGACGGTGCTGAACATGATGACGGG 60

||||||||||||||||||||||||||||||||||||||||||||||||||||||||||||

Sbjct 1923 CTCTGTGCTGCTGGGGCACAACGGCGCCGGCAAGACGACGGTGCTGAACATGATGACGGG 1982

Query 61 GATGGTCGAGCCGG 74

||||||||||||||

Sbjct 1983 GATGGTCGAGCCGG 1996

>LmjF.02.0300:mRNA | gene=LmjF.02.0300 | organism=Leishmania_major_strain_Friedlin

| gene_product=ATP-binding cassette subfamily

A, member 1, putative | transcript_product=ATP-binding

cassette subfamily A, member 1, putative | location=LmjF.02:138486-146384(-)

| length=7899 | sequence_SO=chromosome |

SO=protein_coding_gene | is_pseudo=false

Length=7899

Score = 94.2 bits (103), Expect = 7e-20

Identities = 65/74 (88%), Gaps = 0/74 (0%)

Strand=Plus/Plus

Query 1 CTCTGTGCTGCTGGGGCACAACGGCGCCGGCAAGACGACGGTGCTGAACATGATGACGGG 60

||| |||||||||||||||||||||||||||||| ||||| || | ||||||||||||||

Sbjct 3705 CTCCGTGCTGCTGGGGCACAACGGCGCCGGCAAGTCGACGCTGATCAACATGATGACGGG 3764

Query 61 GATGGTCGAGCCGG 74

||| || | ||||

Sbjct 3765 CATGCTCTACCCGG 3778

>LmjF.27.0980:mRNA | gene=LmjF.27.0980 | organism=Leishmania_major_strain_Friedlin

| gene_product=ATP-binding cassette protein

subfamily A, member 9, putative | transcript_product=ATP-binding

cassette protein subfamily A, member 9, putative |

location=LmjF.27:421573-427215(-) | length=5643 | sequence_SO=chromosome

| SO=protein_coding_gene | is_pseudo=false

Length=5643

Score = 82.4 bits (90), Expect = 1e-16

Identities = 60/70 (86%), Gaps = 0/70 (0%)

Strand=Plus/Plus

Query 1 CTCTGTGCTGCTGGGGCACAACGGCGCCGGCAAGACGACGGTGCTGAACATGATGACGGG 60

||||||||||||||| ||||||||||| |||||| ||||| | ||||| ||||||| ||

Sbjct 2376 CTCTGTGCTGCTGGGCCACAACGGCGCGGGCAAGTCGACGACGATGAACCTGATGACCGG 2435

Query 61 GATGGTCGAG 70

|||| | |||

Sbjct 2436 GATGCTGGAG 2445

Score = 37.4 bits (40), Expect = 0.005

Identities = 26/30 (87%), Gaps = 0/30 (0%)

Strand=Plus/Plus

Query 11 CTGGGGCACAACGGCGCCGGCAAGACGACG 40

|||||| |||||||| ||||||||||||

Sbjct 4738 CTGGGGACGAACGGCGCGGGCAAGACGACG 4767

>LmjF.27.0970:mRNA | gene=LmjF.27.0970 | organism=Leishmania_major_strain_Friedlin

| gene_product=ATP-binding cassette protein

subfamily A, member 8, putative | transcript_product=ATP-binding

cassette protein subfamily A, member 8, putative |

location=LmjF.27:410527-416061(-) | length=5535 | sequence_SO=chromosome

| SO=protein_coding_gene | is_pseudo=false

Length=5535

Score = 82.4 bits (90), Expect = 1e-16

Identities = 60/70 (86%), Gaps = 0/70 (0%)

Strand=Plus/Plus

Query 1 CTCTGTGCTGCTGGGGCACAACGGCGCCGGCAAGACGACGGTGCTGAACATGATGACGGG 60

||||||||||||||| ||||||||||| |||||| ||||| | ||||| ||||||| ||

Sbjct 2250 CTCTGTGCTGCTGGGCCACAACGGCGCGGGCAAGTCGACGACGATGAACCTGATGACCGG 2309

Query 61 GATGGTCGAG 70

|||| | |||

Sbjct 2310 GATGCTGGAG 2319

Score = 37.4 bits (40), Expect = 0.005

Identities = 26/30 (87%), Gaps = 0/30 (0%)

Strand=Plus/Plus

Query 11 CTGGGGCACAACGGCGCCGGCAAGACGACG 40

|||||| |||||||| ||||||||||||

Sbjct 4612 CTGGGGACGAACGGCGCGGGCAAGACGACG 4641

>LmjF.29.0620:mRNA | gene=LmjF.29.0620 | organism=Leishmania_major_strain_Friedlin

| gene_product=ATP-binding cassette protein

subfamily A, member 10, putative | transcript_product=ATP-binding

cassette protein subfamily A, member 10, putative

| location=LmjF.29:230353-235953(-) | length=5601 | sequence_SO=chromosome

| SO=protein_coding_gene | is_pseudo=false

Length=5601

Score = 58.1 bits (63), Expect = 5e-09

Identities = 39/44 (89%), Gaps = 0/44 (0%)

Strand=Plus/Plus

Query 8 CTGCTGGGGCACAACGGCGCCGGCAAGACGACGGTGCTGAACAT 51

|||||||||| |||||||||||||||||||||| | ||| |||

Sbjct 4711 CTGCTGGGGCTTAACGGCGCCGGCAAGACGACGGCGGTGAGCAT 4754

>LmjF.30.1330:mRNA | gene=LmjF.30.1330 | organism=Leishmania_major_strain_Friedlin

| gene_product=ATP-binding cassette protein

subfamily H, member 3, putative | transcript_product=ATP-binding

cassette protein subfamily H, member 3, putative |

location=LmjF.30:461242-462843(+) | length=1602 | sequence_SO=chromosome

| SO=protein_coding_gene | is_pseudo=false

Length=1602

Score = 38.3 bits (41), Expect = 0.005

Identities = 28/33 (85%), Gaps = 0/33 (0%)

Strand=Plus/Plus

Query 20 AACGGCGCCGGCAAGACGACGGTGCTGAACATG 52

|||||| |||||||| | || |||||||||||

Sbjct 1006 AACGGCACCGGCAAGTCCACCTTGCTGAACATG 1038

Score = 33.7 bits (36), Expect = 0.058

Identities = 27/33 (82%), Gaps = 0/33 (0%)

Strand=Plus/Plus

Query 14 GGGCACAACGGCGCCGGCAAGACGACGGTGCTG 46

||||||||||| ||||||| |||| |||||

Sbjct 148 GGGCACAACGGGAGCGGCAAGTCGACACTGCTG 180

>LmjF.34.4280:mRNA | gene=LmjF.34.4280 | organism=Leishmania_major_strain_Friedlin

| gene_product=Ankyrin repeats (3 copies),

putative | transcript_product=Ankyrin repeats (3 copies),

putative | location=LmjF.34:1800878-1802095(+) | length=1218

| sequence_SO=chromosome | SO=protein_coding_gene | is_pseudo=false

Length=1218

Score = 35.6 bits (38), Expect = 0.017

Identities = 22/24 (92%), Gaps = 0/24 (0%)

Strand=Plus/Plus

Query 3 CTGTGCTGCTGGGGCACAACGGCG 26

|||||||||||| || ||||||||

Sbjct 614 CTGTGCTGCTGGTGCTCAACGGCG 637

>LmjF.32.2810:mRNA | gene=LmjF.32.2810 | organism=Leishmania_major_strain_Friedlin

| gene_product=RNA-binding protein, putative

| transcript_product=RNA-binding protein, putative | location=LmjF.32:1105559-1106902(-)

| length=1344 | sequence_SO=chromosome

| SO=protein_coding_gene | is_pseudo=false

Length=1344

Score = 35.6 bits (38), Expect = 0.017

Identities = 28/34 (82%), Gaps = 0/34 (0%)

Strand=Plus/Plus

Query 4 TGTGCTGCTGGGGCACAACGGCGCCGGCAAGACG 37

||||||||||| || |||| | || ||||||||

Sbjct 423 TGTGCTGCTGGTGCGCAACCGTGCGAGCAAGACG 456

>LmjF.33.1860:mRNA | gene=LmjF.33.1860 | organism=Leishmania_major_strain_Friedlin

| gene_product=glycosomal transporter (GAT2),

putative | transcript_product=glycosomal transporter

(GAT2), putative | location=LmjF.33:838595-840517(+) | length=1923

| sequence_SO=chromosome | SO=protein_coding_gene | is_pseudo=false

Length=1923

Score = 33.7 bits (36), Expect = 0.058

Identities = 23/25 (92%), Gaps = 1/25 (4%)

Strand=Plus/Plus

Query 14 GGGCA-CAACGGCGCCGGCAAGACG 37

||||| ||||||| |||||||||||

Sbjct 1368 GGGCAGCAACGGCTCCGGCAAGACG 1392

>LmjF.12.0610:mRNA | gene=LmjF.12.0610 | organism=Leishmania_major_strain_Friedlin

| gene_product=TPR repeat, putative | transcript_product=TPR

repeat, putative | location=LmjF.12:333132-336401(+)

| length=3270 | sequence_SO=chromosome | SO=protein_coding_gene

| is_pseudo=false

Length=3270

Score = 33.7 bits (36), Expect = 0.058

Identities = 18/18 (100%), Gaps = 0/18 (0%)

Strand=Plus/Minus

Query 56 ACGGGGATGGTCGAGCCG 73

||||||||||||||||||

Sbjct 3134 ACGGGGATGGTCGAGCCG 3117

>LmjF.06.0090:mRNA | gene=LmjF.06.0090 | organism=Leishmania_major_strain_Friedlin

| gene_product=ATP-binding cassette protein

subfamily G, member 2 | transcript_product=ATP-binding

cassette protein subfamily G, member 2 | location=LmjF.06:40263-42254(-)

| length=1992 | sequence_SO=chromosome | SO=protein_coding_gene

| is_pseudo=false

Length=1992

Score = 33.7 bits (36), Expect = 0.058

Identities = 24/28 (86%), Gaps = 0/28 (0%)

Strand=Plus/Plus

Query 22 CGGCGCCGGCAAGACGACGGTGCTGAAC 49

||| ||||||||||| ||| | ||||||

Sbjct 312 CGGTGCCGGCAAGACAACGTTCCTGAAC 339

>LmjF.06.0080:mRNA | gene=LmjF.06.0080 | organism=Leishmania_major_strain_Friedlin

| gene_product=ATP-binding cassette protein

subfamily G, member 1, putative | transcript_product=ATP-binding

cassette protein subfamily G, member 1, putative |

location=LmjF.06:34880-36853(-) | length=1974 | sequence_SO=chromosome

| SO=protein_coding_gene | is_pseudo=false

Length=1974

Score = 33.7 bits (36), Expect = 0.058

Identities = 24/28 (86%), Gaps = 0/28 (0%)

Strand=Plus/Plus

Query 22 CGGCGCCGGCAAGACGACGGTGCTGAAC 49

||| ||||||||||| ||| | ||||||

Sbjct 297 CGGTGCCGGCAAGACAACGTTCCTGAAC 324

>LmjF.05.0910:mRNA | gene=LmjF.05.0910 | organism=Leishmania_major_strain_Friedlin

| gene_product=hypothetical protein, conserved

| transcript_product=hypothetical protein, conserved

| location=LmjF.05:334884-338120(+) | length=3237 | sequence_SO=chromosome

| SO=protein_coding_gene | is_pseudo=false

Length=3237

Score = 32.8 bits (35), Expect = 0.20

Identities = 30/37 (81%), Gaps = 1/37 (3%)

Strand=Plus/Plus

Query 36 CGACGGTGCTGAACATGATGACGGGGATGGTCGAGCC 72

||||| ||| |||||| || || ||||||||||||

Sbjct 1797 CGACG-TGCAGAACATCATCCAGGAGATGGTCGAGCC 1832

Score = 27.4 bits (29), Expect = 8.7

Identities = 16/17 (94%), Gaps = 0/17 (0%)

Strand=Plus/Minus

Query 55 GACGGGGATGGTCGAGC 71

|||||||||||| ||||

Sbjct 1251 GACGGGGATGGTGGAGC 1235

>LmjF.28.0220:mRNA | gene=LmjF.28.0220 | organism=Leishmania_major_strain_Friedlin

| gene_product=palmitoyl acyltransferase

9, putative | transcript_product=palmitoyl acyltransferase

9, putative | location=LmjF.28:69153-70220(+) | length=1068

| sequence_SO=chromosome | SO=protein_coding_gene | is_pseudo=false

Length=1068

Score = 31.9 bits (34), Expect = 0.20

Identities = 20/22 (91%), Gaps = 0/22 (0%)

Strand=Plus/Minus

Query 31 CAAGACGACGGTGCTGAACATG 52

||||||||||||| || |||||

Sbjct 312 CAAGACGACGGTGATGCACATG 291

>LmjF.21.1210:mRNA | gene=LmjF.21.1210 | organism=Leishmania_major_strain_Friedlin

| gene_product=thymidine kinase, putative

| transcript_product=thymidine kinase, putative | location=LmjF.21:497568-498422(-)

| length=855 | sequence_SO=chromosome

| SO=protein_coding_gene | is_pseudo=false

Length=855

Score = 31.9 bits (34), Expect = 0.20

Identities = 20/22 (91%), Gaps = 0/22 (0%)

Strand=Plus/Plus

Query 26 GCCGGCAAGACGACGGTGCTGA 47

||||||||||| |||| |||||

Sbjct 43 GCCGGCAAGACAACGGAGCTGA 64

>LmjF.14.1060:mRNA | gene=LmjF.14.1060 | organism=Leishmania_major_strain_Friedlin

| gene_product=dynein heavy chain, putative

| transcript_product=dynein heavy chain, putative | location=LmjF.14:428203-441009(+)

| length=12807 | sequence_SO=chromosome

| SO=protein_coding_gene | is_pseudo=false

Length=12807

Score = 31.9 bits (34), Expect = 0.20

Identities = 29/37 (78%), Gaps = 0/37 (0%)

Strand=Plus/Plus

Query 6 TGCTGCTGGGGCACAACGGCGCCGGCAAGACGACGGT 42

||||| |||| |||| | | ||||||||||||||

Sbjct 4721 TGCTGGTGGGCCACACCATGACGGGCAAGACGACGGT 4757

>LmjF.08.0430:mRNA | gene=LmjF.08.0430 | organism=Leishmania_major_strain_Friedlin

| gene_product=hypothetical protein, conserved

| transcript_product=hypothetical protein, conserved

| location=LmjF.08:173458-176235(+) | length=2778 | sequence_SO=chromosome

| SO=protein_coding_gene | is_pseudo=false

Length=2778

Score = 31.9 bits (34), Expect = 0.20

Identities = 20/22 (91%), Gaps = 0/22 (0%)

Strand=Plus/Plus

Query 28 CGGCAAGACGACGGTGCTGAAC 49

||||||| || |||||||||||

Sbjct 2382 CGGCAAGCCGGCGGTGCTGAAC 2403

>LmjF.36.6030:mRNA | gene=LmjF.36.6030 | organism=Leishmania_major_strain_Friedlin

| gene_product=hypothetical protein, conserved

| transcript_product=hypothetical protein, conserved

| location=LmjF.36:2316752-2323792(-) | length=7041 | sequence_SO=chromosome

| SO=protein_coding_gene | is_pseudo=false

Length=7041

Score = 30.1 bits (32), Expect = 0.71

Identities = 16/16 (100%), Gaps = 0/16 (0%)

Strand=Plus/Minus

Query 47 AACATGATGACGGGGA 62

||||||||||||||||

Sbjct 6341 AACATGATGACGGGGA 6326

>LmjF.34.0310:mRNA | gene=LmjF.34.0310 | organism=Leishmania_major_strain_Friedlin

| gene_product=tRNA pseudouridine synthase,

putative | transcript_product=tRNA pseudouridine synthase,

putative | location=LmjF.34:111182-113287(+) | length=2106

| sequence_SO=chromosome | SO=protein_coding_gene | is_pseudo=false

Length=2106

Score = 30.1 bits (32), Expect = 0.71

Identities = 16/16 (100%), Gaps = 0/16 (0%)

Strand=Plus/Plus

Query 18 ACAACGGCGCCGGCAA 33

||||||||||||||||

Sbjct 1922 ACAACGGCGCCGGCAA 1937

>LmjF.33.2610:mRNA | gene=LmjF.33.2610 | organism=Leishmania_major_strain_Friedlin

| gene_product=Mitochondrial-processing

peptidase subunit alpha | transcript_product=Mitochondrial-processing

peptidase subunit alpha | location=LmjF.33:1172034-1173485(+)

| length=1452 | sequence_SO=chromosome | SO=protein_coding_gene

| is_pseudo=false

Length=1452

Score = 30.1 bits (32), Expect = 0.71

Identities = 19/21 (90%), Gaps = 0/21 (0%)

Strand=Plus/Minus

Query 19 CAACGGCGCCGGCAAGACGAC 39

||||| ||| |||||||||||

Sbjct 34 CAACGCCGCGGGCAAGACGAC 14

>LmjF.32.3870:mRNA | gene=LmjF.32.3870 | organism=Leishmania_major_strain_Friedlin

| gene_product=myosin XXI, putative | transcript_product=myosin

XXI, putative | location=LmjF.32:1545731-1548883(+)

| length=3153 | sequence_SO=chromosome | SO=protein_coding_gene

| is_pseudo=false

Length=3153

Score = 30.1 bits (32), Expect = 0.71

Identities = 22/26 (85%), Gaps = 0/26 (0%)

Strand=Plus/Plus

Query 18 ACAACGGCGCCGGCAAGACGACGGTG 43

|| ||| || ||||||||||| ||||

Sbjct 2180 ACTACGCCGTCGGCAAGACGAAGGTG 2205

>LmjF.32.1110:mRNA | gene=LmjF.32.1110 | organism=Leishmania_major_strain_Friedlin

| gene_product=mitochondrial carrier protein,

putative | transcript_product=mitochondrial carrier protein,

putative | location=LmjF.32:437197-438294(+) | length=1098

| sequence_SO=chromosome | SO=protein_coding_gene | is_pseudo=false

Length=1098

Score = 30.1 bits (32), Expect = 0.71

Identities = 16/16 (100%), Gaps = 0/16 (0%)

Strand=Plus/Minus

Query 18 ACAACGGCGCCGGCAA 33

||||||||||||||||

Sbjct 443 ACAACGGCGCCGGCAA 428

>LmjF.30.2210:mRNA | gene=LmjF.30.2210 | organism=Leishmania_major_strain_Friedlin

| gene_product=endosomal trafficking protein

RME-8, putative | transcript_product=endosomal trafficking

protein RME-8, putative | location=LmjF.30:844914-852290(-)

| length=7377 | sequence_SO=chromosome | SO=protein_coding_gene

| is_pseudo=false

Length=7377

Score = 30.1 bits (32), Expect = 0.71

Identities = 16/16 (100%), Gaps = 0/16 (0%)

Strand=Plus/Minus

Query 24 GCGCCGGCAAGACGAC 39

||||||||||||||||

Sbjct 1280 GCGCCGGCAAGACGAC 1265

>LmjF.16.0980:mRNA | gene=LmjF.16.0980 | organism=Leishmania_major_strain_Friedlin

| gene_product=hypothetical protein, conserved

| transcript_product=hypothetical protein, conserved

| location=LmjF.16:356749-360210(+) | length=3462 | sequence_SO=chromosome

| SO=protein_coding_gene | is_pseudo=false

Length=3462

Score = 30.1 bits (32), Expect = 0.71

Identities = 19/21 (90%), Gaps = 0/21 (0%)

Strand=Plus/Plus

Query 21 ACGGCGCCGGCAAGACGACGG 41

||| |||||||||||||| ||

Sbjct 1580 ACGCCGCCGGCAAGACGAAGG 1600

>LmjF.36.3170:mRNA | gene=LmjF.36.3170 | organism=Leishmania_major_strain_Friedlin

| gene_product=CobW/HypB/UreG, nucleotide-binding

domain containing protein, putative | transcript_product=CobW/HypB/UreG,

nucleotide-binding domain containing

protein, putative | location=LmjF.36:1260926-1261960(-) | length=1035

| sequence_SO=chromosome | SO=protein_coding_gene

| is_pseudo=false

Length=1035

Score = 29.2 bits (31), Expect = 2.5

Identities = 23/28 (82%), Gaps = 0/28 (0%)

Strand=Plus/Plus

Query 22 CGGCGCCGGCAAGACGACGGTGCTGAAC 49

|||| ||||||||||||| || || ||

Sbjct 72 CGGCAGCGGCAAGACGACGCTGTTGCAC 99

>LmjF.27.1750:mRNA | gene=LmjF.27.1750 | organism=Leishmania_major_strain_Friedlin

| gene_product=Cytoplasmic dynein 2 heavy

chain (DYNC2H1), putative | transcript_product=Cytoplasmic

dynein 2 heavy chain (DYNC2H1), putative | location=LmjF.27:720668-734032(-)

| length=13365 | sequence_SO=chromosome |

SO=protein_coding_gene | is_pseudo=false

Length=13365

Score = 29.2 bits (31), Expect = 2.5

Identities = 17/18 (94%), Gaps = 0/18 (0%)

Strand=Plus/Plus

Query 42 TGCTGAACATGATGACGG 59

||||||||||||| ||||

Sbjct 3995 TGCTGAACATGATCACGG 4012

>LmjF.22.1240:mRNA | gene=LmjF.22.1240 | organism=Leishmania_major_strain_Friedlin

| gene_product=translation initiation factor

IF-2, putative | transcript_product=translation initiation

factor IF-2, putative | location=LmjF.22:513265-515799(-)

| length=2535 | sequence_SO=chromosome | SO=protein_coding_gene

| is_pseudo=false

Length=2535

Score = 29.2 bits (31), Expect = 2.5

Identities = 17/18 (94%), Gaps = 0/18 (0%)

Strand=Plus/Plus

Query 29 GGCAAGACGACGGTGCTG 46

|||||||||||| |||||

Sbjct 784 GGCAAGACGACGCTGCTG 801

>LmjF.21.0680:mRNA | gene=LmjF.21.0680 | organism=Leishmania_major_strain_Friedlin

| gene_product=histone deacetylase, putative

| transcript_product=histone deacetylase, putative | location=LmjF.21:209387-210673(-)

| length=1287 | sequence_SO=chromosome

| SO=protein_coding_gene | is_pseudo=false

Length=1287

Score = 29.2 bits (31), Expect = 2.5

Identities = 17/18 (94%), Gaps = 0/18 (0%)

Strand=Plus/Minus

Query 9 TGCTGGGGCACAACGGCG 26

||||||||||||| ||||

Sbjct 128 TGCTGGGGCACAAAGGCG 111

>LmjF.16.0370:mRNA | gene=LmjF.16.0370 | organism=Leishmania_major_strain_Friedlin

| gene_product=hypothetical protein, conserved

| transcript_product=hypothetical protein, conserved

| location=LmjF.16:129105-130571(-) | length=1467 | sequence_SO=chromosome

| SO=protein_coding_gene | is_pseudo=false

Length=1467

Score = 29.2 bits (31), Expect = 2.5

Identities = 17/18 (94%), Gaps = 0/18 (0%)

Strand=Plus/Plus

Query 5 GTGCTGCTGGGGCACAAC 22

||||||||||||| ||||

Sbjct 1135 GTGCTGCTGGGGCCCAAC 1152

>LmjF.14.1490:mRNA | gene=LmjF.14.1490 | organism=Leishmania_major_strain_Friedlin

| gene_product=synaptojanin (N-terminal

domain), putative | transcript_product=synaptojanin (N-terminal

domain), putative | location=LmjF.14:617185-619722(+) |

length=2538 | sequence_SO=chromosome | SO=protein_coding_gene

| is_pseudo=false

Length=2538

Score = 29.2 bits (31), Expect = 2.5

Identities = 17/18 (94%), Gaps = 0/18 (0%)

Strand=Plus/Plus

Query 5 GTGCTGCTGGGGCACAAC 22

||||||||||||| ||||

Sbjct 1933 GTGCTGCTGGGGCGCAAC 1950

Score = 27.4 bits (29), Expect = 8.7

Identities = 18/19 (95%), Gaps = 1/19 (5%)

Strand=Plus/Plus

Query 15 GGCACAACGGCGCCGGCAA 33

|||| ||||||||||||||

Sbjct 864 GGCA-AACGGCGCCGGCAA 881

>LmjF.11.0320:mRNA | gene=LmjF.11.0320 | organism=Leishmania_major_strain_Friedlin

| gene_product=DNA repair and recombination

helicase protein PIF2, putative | transcript_product=DNA

repair and recombination helicase protein PIF2, putative |

location=LmjF.11:92896-97653(+) | length=4758 | sequence_SO=chromosome

| SO=protein_coding_gene | is_pseudo=false

Length=4758

Score = 29.2 bits (31), Expect = 2.5

Identities = 17/18 (94%), Gaps = 0/18 (0%)

Strand=Plus/Plus

Query 30 GCAAGACGACGGTGCTGA 47

|||||| |||||||||||

Sbjct 4544 GCAAGAAGACGGTGCTGA 4561

>LmjF.04.0600:mRNA | gene=LmjF.04.0600 | organism=Leishmania_major_strain_Friedlin

| gene_product=hypothetical protein | transcript_product=hypothetical

protein | location=LmjF.04:243289-252480(-)

| length=9192 | sequence_SO=chromosome | SO=protein_coding_gene

| is_pseudo=false

Length=9192

Score = 29.2 bits (31), Expect = 2.5

Identities = 17/18 (94%), Gaps = 0/18 (0%)

Strand=Plus/Plus

Query 19 CAACGGCGCCGGCAAGAC 36

||| ||||||||||||||

Sbjct 4527 CAAAGGCGCCGGCAAGAC 4544

>LmjF.36.3530:mRNA | gene=LmjF.36.3530 | organism=Leishmania_major_strain_Friedlin

| gene_product=polyubiquitin, putative

| transcript_product=polyubiquitin, putative | location=LmjF.36:1370931-1373444(-)

| length=2514 | sequence_SO=chromosome

| SO=protein_coding_gene | is_pseudo=false

Length=2514

Score = 28.3 bits (30), Expect = 2.5

Identities = 18/20 (90%), Gaps = 0/20 (0%)

Strand=Plus/Plus

Query 27 CCGGCAAGACGACGGTGCTG 46

|||||||||||| ||||||

Sbjct 1166 CCGGCAAGACGATCGTGCTG 1185

>LmjF.34.1000:mRNA | gene=LmjF.34.1000 | organism=Leishmania_major_strain_Friedlin

| gene_product=myosin IB heavy chain, putative

| transcript_product=myosin IB heavy chain, putative

| location=LmjF.34:445059-449180(+) | length=4122 | sequence_SO=chromosome

| SO=protein_coding_gene | is_pseudo=false

Length=4122

Score = 28.3 bits (30), Expect = 2.5

Identities = 15/15 (100%), Gaps = 0/15 (0%)

Strand=Plus/Plus

Query 23 GGCGCCGGCAAGACG 37

|||||||||||||||

Sbjct 373 GGCGCCGGCAAGACG 387

>LmjF.33.2940:mRNA | gene=LmjF.33.2940 | organism=Leishmania_major_strain_Friedlin

| gene_product=hypothetical protein, conserved

| transcript_product=hypothetical protein, conserved

| location=LmjF.33:1406060-1408435(+) | length=2376 | sequence_SO=chromosome

| SO=protein_coding_gene | is_pseudo=false

Length=2376

Score = 28.3 bits (30), Expect = 2.5

Identities = 15/15 (100%), Gaps = 0/15 (0%)

Strand=Plus/Minus

Query 32 AAGACGACGGTGCTG 46

|||||||||||||||

Sbjct 1436 AAGACGACGGTGCTG 1422

>LmjF.32.3450:mRNA | gene=LmjF.32.3450 | organism=Leishmania_major_strain_Friedlin

| gene_product=zinc-finger of a C2HC-type,

putative | transcript_product=zinc-finger of a C2HC-type,

putative | location=LmjF.32:1389213-1391105(+) | length=1893

| sequence_SO=chromosome | SO=protein_coding_gene | is_pseudo=false

Length=1893

Score = 28.3 bits (30), Expect = 2.5

Identities = 15/15 (100%), Gaps = 0/15 (0%)

Strand=Plus/Plus

Query 19 CAACGGCGCCGGCAA 33

|||||||||||||||

Sbjct 1284 CAACGGCGCCGGCAA 1298

>LmjF.32.2150:mRNA | gene=LmjF.32.2150 | organism=Leishmania_major_strain_Friedlin

| gene_product=hypothetical protein, conserved

| transcript_product=hypothetical protein, conserved

| location=LmjF.32:833765-837013(-) | length=3249 | sequence_SO=chromosome

| SO=protein_coding_gene | is_pseudo=false

Length=3249

Score = 28.3 bits (30), Expect = 2.5

Identities = 15/15 (100%), Gaps = 0/15 (0%)

Strand=Plus/Plus

Query 42 TGCTGAACATGATGA 56

|||||||||||||||

Sbjct 1901 TGCTGAACATGATGA 1915

>LmjF.32.0260:mRNA | gene=LmjF.32.0260 | organism=Leishmania_major_strain_Friedlin

| gene_product=Serine/threonine-protein

kinase NEK16, putative | transcript_product=Serine/threonine-protein

kinase NEK16, putative | location=LmjF.32:88901-92950(-)

| length=4050 | sequence_SO=chromosome | SO=protein_coding_gene

| is_pseudo=false

Length=4050

Score = 28.3 bits (30), Expect = 2.5

Identities = 24/30 (80%), Gaps = 0/30 (0%)

Strand=Plus/Plus

Query 36 CGACGGTGCTGAACATGATGACGGGGATGG 65

||||||||||| || || || ||| ||||

Sbjct 446 CGACGGTGCTGCGCACGACGATGGGCATGG 475

>LmjF.28.0940:mRNA | gene=LmjF.28.0940 | organism=Leishmania_major_strain_Friedlin

| gene_product=oxidoreductase-like protein

| transcript_product=oxidoreductase-like protein | location=LmjF.28:344661-347582(+)

| length=2922 | sequence_SO=chromosome

| SO=protein_coding_gene | is_pseudo=false

Length=2922

Score = 28.3 bits (30), Expect = 2.5

Identities = 15/15 (100%), Gaps = 0/15 (0%)

Strand=Plus/Plus

Query 44 CTGAACATGATGACG 58

|||||||||||||||

Sbjct 2680 CTGAACATGATGACG 2694

>LmjF.25.1110:mRNA | gene=LmjF.25.1110 | organism=Leishmania_major_strain_Friedlin

| gene_product=hypothetical protein, conserved

| transcript_product=hypothetical protein, conserved

| location=LmjF.25:429674-449296(-) | length=19623 | sequence_SO=chromosome

| SO=protein_coding_gene | is_pseudo=false

Length=19623

Score = 28.3 bits (30), Expect = 2.5

Identities = 20/22 (91%), Gaps = 1/22 (5%)

Strand=Plus/Plus

Query 35 ACGACGGTGCTGAACATGATGA 56

|||||||||||||| | |||||

Sbjct 3488 ACGACGGTGCTGAAGA-GATGA 3508

>LmjF.20.1190:mRNA | gene=LmjF.20.1190 | organism=Leishmania_major_strain_Friedlin

| gene_product=cysteine peptidase, Clan

CA, family C2, putative | transcript_product=cysteine peptidase,

Clan CA, family C2, putative | location=LmjF.20:541963-544026(+)

| length=2064 | sequence_SO=chromosome | SO=protein_coding_gene

| is_pseudo=false

Length=2064

Score = 28.3 bits (30), Expect = 2.5

Identities = 15/15 (100%), Gaps = 0/15 (0%)

Strand=Plus/Plus

Query 6 TGCTGCTGGGGCACA 20

|||||||||||||||

Sbjct 1235 TGCTGCTGGGGCACA 1249

>LmjF.19.1050:mRNA | gene=LmjF.19.1050 | organism=Leishmania_major_strain_Friedlin

| gene_product=hypothetical protein, unknown

function | transcript_product=hypothetical protein, unknown

function | location=LmjF.19:429043-433092(+) | length=4050

| sequence_SO=chromosome | SO=protein_coding_gene | is_pseudo=false

Length=4050

Score = 28.3 bits (30), Expect = 2.5

Identities = 23/27 (85%), Gaps = 1/27 (4%)

Strand=Plus/Plus

Query 5 GTGCTGCTGGGGCACAACGGCGCCGGC 31

||||||||| ||||||||||||||

Sbjct 1019 GTGCTGCTGATA-ACAACGGCGCCGGC 1044

>LmjF.16.1190:mRNA | gene=LmjF.16.1190 | organism=Leishmania_major_strain_Friedlin

| gene_product=Domain of unknown function

DUF221, putative | transcript_product=Domain of unknown function

DUF221, putative | location=LmjF.16:469429-473754(-)

| length=4326 | sequence_SO=chromosome | SO=protein_coding_gene

| is_pseudo=false

Length=4326

Score = 28.3 bits (30), Expect = 2.5

Identities = 15/15 (100%), Gaps = 0/15 (0%)

Strand=Plus/Plus

Query 37 GACGGTGCTGAACAT 51

|||||||||||||||

Sbjct 1032 GACGGTGCTGAACAT 1046

>LmjF.09.0070:mRNA | gene=LmjF.09.0070 | organism=Leishmania_major_strain_Friedlin

| gene_product=RNA helicase, putative |

transcript_product=RNA helicase, putative | location=LmjF.09:17000-20953(+)

| length=3954 | sequence_SO=chromosome | SO=protein_coding_gene

| is_pseudo=false

Length=3954

Score = 28.3 bits (30), Expect = 2.5

Identities = 18/20 (90%), Gaps = 0/20 (0%)

Strand=Plus/Minus

Query 28 CGGCAAGACGACGGTGCTGA 47

||||||||||||| |||||

Sbjct 1411 CGGCAAGACGACGAGGCTGA 1392

>LmjF.02.0140:mRNA | gene=LmjF.02.0140 | organism=Leishmania_major_strain_Friedlin

| gene_product=Ankyrin repeats (3 copies)/Zinc

finger, C3HC4 type (RING finger), putative | transcript_product=Ankyrin

repeats (3 copies)/Zinc finger, C3HC4 type

(RING finger), putative | location=LmjF.02:61959-64646(-)

| length=2688 | sequence_SO=chromosome | SO=protein_coding_gene

| is_pseudo=false

Length=2688

Score = 28.3 bits (30), Expect = 2.5

Identities = 15/15 (100%), Gaps = 0/15 (0%)

Strand=Plus/Plus

Query 5 GTGCTGCTGGGGCAC 19

|||||||||||||||

Sbjct 2090 GTGCTGCTGGGGCAC 2104

>LmjF.01.0670:mRNA | gene=LmjF.01.0670 | organism=Leishmania_major_strain_Friedlin

| gene_product=hypothetical protein, conserved

| transcript_product=hypothetical protein, conserved

| location=LmjF.01:194045-194962(+) | length=918 | sequence_SO=chromosome

| SO=protein_coding_gene | is_pseudo=false

Length=918

Score = 28.3 bits (30), Expect = 2.5

Identities = 15/15 (100%), Gaps = 0/15 (0%)

Strand=Plus/Plus

Query 19 CAACGGCGCCGGCAA 33

|||||||||||||||

Sbjct 228 CAACGGCGCCGGCAA 242

>LmjF.36.4290:mRNA | gene=LmjF.36.4290 | organism=Leishmania_major_strain_Friedlin

| gene_product=ADP-ribosylation factor

family, putative | transcript_product=ADP-ribosylation factor

family, putative | location=LmjF.36:1650408-1654100(+) | length=3693

| sequence_SO=chromosome | SO=protein_coding_gene

| is_pseudo=false

Length=3693

Score = 27.4 bits (29), Expect = 8.7

Identities = 16/17 (94%), Gaps = 0/17 (0%)

Strand=Plus/Plus

Query 24 GCGCCGGCAAGACGACG 40

|||| ||||||||||||

Sbjct 1973 GCGCTGGCAAGACGACG 1989

>LmjF.34.3610:mRNA | gene=LmjF.34.3610 | organism=Leishmania_major_strain_Friedlin

| gene_product=Complex 1 protein (LYR family),

putative | transcript_product=Complex 1 protein (LYR

family), putative | location=LmjF.34:1564089-1564610(+) | length=522

| sequence_SO=chromosome | SO=protein_coding_gene

| is_pseudo=false

Length=522

Score = 27.4 bits (29), Expect = 8.7

Identities = 16/17 (94%), Gaps = 0/17 (0%)

Strand=Plus/Plus

Query 55 GACGGGGATGGTCGAGC 71

|||| ||||||||||||

Sbjct 460 GACGTGGATGGTCGAGC 476

>LmjF.32.3740:mRNA | gene=LmjF.32.3740 | organism=Leishmania_major_strain_Friedlin

| gene_product=hypothetical protein, conserved

| transcript_product=hypothetical protein, conserved

| location=LmjF.32:1516227-1516694(+) | length=468 | sequence_SO=chromosome

| SO=protein_coding_gene | is_pseudo=false

Length=468

Score = 27.4 bits (29), Expect = 8.7

Identities = 16/17 (94%), Gaps = 0/17 (0%)

Strand=Plus/Minus

Query 36 CGACGGTGCTGAACATG 52

|| ||||||||||||||

Sbjct 439 CGTCGGTGCTGAACATG 423

>LmjF.28.1880:mRNA | gene=LmjF.28.1880 | organism=Leishmania_major_strain_Friedlin

| gene_product=Nucleolar protein 136, putative

| transcript_product=Nucleolar protein 136, putative

| location=LmjF.28:726026-729811(-) | length=3786 | sequence_SO=chromosome

| SO=protein_coding_gene | is_pseudo=false

Length=3786

Score = 27.4 bits (29), Expect = 8.7

Identities = 19/22 (86%), Gaps = 0/22 (0%)

Strand=Plus/Plus

Query 22 CGGCGCCGGCAAGACGACGGTG 43

||||| |||||||||||| ||

Sbjct 285 CGGCGTTGGCAAGACGACGCTG 306

>LmjF.28.1460:mRNA | gene=LmjF.28.1460 | organism=Leishmania_major_strain_Friedlin

| gene_product=Cell division control protein

48, putative | transcript_product=Cell division control

protein 48, putative | location=LmjF.28:551135-553450(+) |

length=2316 | sequence_SO=chromosome | SO=protein_coding_gene

| is_pseudo=false

Length=2316

Score = 27.4 bits (29), Expect = 8.7

Identities = 16/17 (94%), Gaps = 0/17 (0%)

Strand=Plus/Plus

Query 22 CGGCGCCGGCAAGACGA 38

|||| ||||||||||||

Sbjct 1374 CGGCACCGGCAAGACGA 1390

>LmjF.26.2410:mRNA | gene=LmjF.26.2410 | organism=Leishmania_major_strain_Friedlin

| gene_product=hypothetical protein, conserved

| transcript_product=hypothetical protein, conserved

| location=LmjF.26:981968-983293(+) | length=1326 | sequence_SO=chromosome

| SO=protein_coding_gene | is_pseudo=false

Length=1326

Score = 27.4 bits (29), Expect = 8.7

Identities = 16/17 (94%), Gaps = 0/17 (0%)

Strand=Plus/Plus

Query 31 CAAGACGACGGTGCTGA 47

|| ||||||||||||||

Sbjct 1125 CACGACGACGGTGCTGA 1141

>LmjF.25.0770:mRNA | gene=LmjF.25.0770 | organism=Leishmania_major_strain_Friedlin

| gene_product=hypothetical protein, unknown

function | transcript_product=hypothetical protein, unknown

function | location=LmjF.25:286874-288424(+) | length=1551

| sequence_SO=chromosome | SO=protein_coding_gene | is_pseudo=false

Length=1551

Score = 27.4 bits (29), Expect = 8.7

Identities = 16/17 (94%), Gaps = 0/17 (0%)

Strand=Plus/Plus

Query 17 CACAACGGCGCCGGCAA 33

||||||||||| |||||

Sbjct 1300 CACAACGGCGCTGGCAA 1316

>LmjF.25.0530:mRNA | gene=LmjF.25.0530 | organism=Leishmania_major_strain_Friedlin

| gene_product=ABC transporter, mitochondrial,

putative | transcript_product=ABC transporter, mitochondrial,

putative | location=LmjF.25:188970-190940(-) | length=1971

| sequence_SO=chromosome | SO=protein_coding_gene |

is_pseudo=false

Length=1971

Score = 27.4 bits (29), Expect = 8.7

Identities = 16/17 (94%), Gaps = 0/17 (0%)

Strand=Plus/Minus

Query 17 CACAACGGCGCCGGCAA 33

||| |||||||||||||

Sbjct 240 CACCACGGCGCCGGCAA 224

>LmjF.24.1900:mRNA | gene=LmjF.24.1900 | organism=Leishmania_major_strain_Friedlin

| gene_product=WD domain, G-beta repeat/PFU

(PLAA family ubiquitin binding), putative | transcript_product=WD

domain, G-beta repeat/PFU (PLAA family ubiquitin

binding), putative | location=LmjF.24:712458-714944(-) | length=2487

| sequence_SO=chromosome | SO=protein_coding_gene |

is_pseudo=false

Length=2487

Score = 27.4 bits (29), Expect = 8.7

Identities = 19/22 (86%), Gaps = 0/22 (0%)

Strand=Plus/Minus

Query 16 GCACAACGGCGCCGGCAAGACG 37

||||||||||| |||| || ||

Sbjct 2239 GCACAACGGCGACGGCCAGCCG 2218

>LmjF.24.0660:mRNA | gene=LmjF.24.0660 | organism=Leishmania_major_strain_Friedlin

| gene_product=hypothetical protein, conserved

| transcript_product=hypothetical protein, conserved

| location=LmjF.24:230994-233285(+) | length=2292 | sequence_SO=chromosome

| SO=protein_coding_gene | is_pseudo=false

Length=2292

Score = 27.4 bits (29), Expect = 8.7

Identities = 16/17 (94%), Gaps = 0/17 (0%)

Strand=Plus/Plus

Query 19 CAACGGCGCCGGCAAGA 35

|||| ||||||||||||

Sbjct 30 CAACCGCGCCGGCAAGA 46

>LmjF.23.1270:mRNA | gene=LmjF.23.1270 | organism=Leishmania_major_strain_Friedlin

| gene_product=XPG N-terminal domain/XPG

I-region, putative | transcript_product=XPG N-terminal domain/XPG

I-region, putative | location=LmjF.23:598664-601705(+)

| length=3042 | sequence_SO=chromosome | SO=protein_coding_gene

| is_pseudo=false

Length=3042

Score = 27.4 bits (29), Expect = 8.7

Identities = 16/17 (94%), Gaps = 0/17 (0%)

Strand=Plus/Minus

Query 15 GGCACAACGGCGCCGGC 31

|| ||||||||||||||

Sbjct 265 GGGACAACGGCGCCGGC 249

>LmjF.23.0830:mRNA | gene=LmjF.23.0830 | organism=Leishmania_major_strain_Friedlin

| gene_product=Na/H antiporter-like protein

| transcript_product=Na/H antiporter-like protein | location=LmjF.23:405212-409714(-)

| length=4503 | sequence_SO=chromosome

| SO=protein_coding_gene | is_pseudo=false

Length=4503

Score = 27.4 bits (29), Expect = 8.7

Identities = 16/17 (94%), Gaps = 0/17 (0%)

Strand=Plus/Plus

Query 36 CGACGGTGCTGAACATG 52

|| ||||||||||||||

Sbjct 1085 CGCCGGTGCTGAACATG 1101

>LmjF.16.1340:mRNA | gene=LmjF.16.1340 | organism=Leishmania_major_strain_Friedlin

| gene_product=hypothetical protein, conserved

| transcript_product=hypothetical protein, conserved

| location=LmjF.16:544494-555770(-) | length=11277 | sequence_SO=chromosome

| SO=protein_coding_gene | is_pseudo=false

Length=11277

Score = 27.4 bits (29), Expect = 8.7

Identities = 19/22 (86%), Gaps = 0/22 (0%)

Strand=Plus/Plus

Query 22 CGGCGCCGGCAAGACGACGGTG 43

||||||||||||| | |||||

Sbjct 5220 CGGCGCCGGCAAGCCCCCGGTG 5241

>LmjF.15.1330:mRNA | gene=LmjF.15.1330 | organism=Leishmania_major_strain_Friedlin

| gene_product=actin-like protein, putative

| transcript_product=actin-like protein, putative | location=LmjF.15:556811-558133(-)

| length=1323 | sequence_SO=chromosome

| SO=protein_coding_gene | is_pseudo=false

Length=1323

Score = 27.4 bits (29), Expect = 8.7

Identities = 19/22 (86%), Gaps = 0/22 (0%)

Strand=Plus/Plus

Query 25 CGCCGGCAAGACGACGGTGCTG 46

||||| | |||||||||||||

Sbjct 225 CGCCGCCGCGACGACGGTGCTG 246

>LmjF.36.4150:mRNA | gene=LmjF.36.4150 | organism=Leishmania_major_strain_Friedlin

| gene_product=hypothetical protein, conserved

| transcript_product=hypothetical protein, conserved

| location=LmjF.36:1582974-1584935(+) | length=1962 | sequence_SO=chromosome

| SO=protein_coding_gene | is_pseudo=false

Length=1962

Score = 26.5 bits (28), Expect = 8.7

Identities = 14/14 (100%), Gaps = 0/14 (0%)

Strand=Plus/Plus

Query 17 CACAACGGCGCCGG 30

||||||||||||||

Sbjct 216 CACAACGGCGCCGG 229

>LmjF.36.2880:mRNA | gene=LmjF.36.2880 | organism=Leishmania_major_strain_Friedlin

| gene_product=hypothetical protein, conserved

| transcript_product=hypothetical protein, conserved

| location=LmjF.36:1175100-1180253(-) | length=5154 | sequence_SO=chromosome

| SO=protein_coding_gene | is_pseudo=false

Length=5154

Score = 26.5 bits (28), Expect = 8.7

Identities = 20/24 (83%), Gaps = 0/24 (0%)

Strand=Plus/Minus

Query 20 AACGGCGCCGGCAAGACGACGGTG 43

||||||||||| || || |||||

Sbjct 3584 AACGGCGCCGGGCAGTCGCCGGTG 3561

>LmjF.35.4910:mRNA | gene=LmjF.35.4910 | organism=Leishmania_major_strain_Friedlin

| gene_product=DNA replication licensing

factor MCM9, putative | transcript_product=DNA replication

licensing factor MCM9, putative | location=LmjF.35:1909194-1911920(+)

| length=2727 | sequence_SO=chromosome | SO=protein_coding_gene

| is_pseudo=false

Length=2727

Score = 26.5 bits (28), Expect = 8.7

Identities = 14/14 (100%), Gaps = 0/14 (0%)

Strand=Plus/Plus

Query 24 GCGCCGGCAAGACG 37

||||||||||||||

Sbjct 860 GCGCCGGCAAGACG 873

>LmjF.35.2950:mRNA | gene=LmjF.35.2950 | organism=Leishmania_major_strain_Friedlin

| gene_product=pentatricopeptide repeat

domain containing protein, putative | transcript_product=pentatricopeptide

repeat domain containing protein, putative |

location=LmjF.35:1206804-1210094(-) | length=3291 | sequence_SO=chromosome

| SO=protein_coding_gene | is_pseudo=false

Length=3291

Score = 26.5 bits (28), Expect = 8.7

Identities = 14/14 (100%), Gaps = 0/14 (0%)

Strand=Plus/Minus

Query 31 CAAGACGACGGTGC 44

||||||||||||||

Sbjct 2685 CAAGACGACGGTGC 2672

>LmjF.34.2980:mRNA | gene=LmjF.34.2980 | organism=Leishmania_major_strain_Friedlin

| gene_product=Nucleoside diphosphate kinase

3 | transcript_product=Nucleoside diphosphate kinase 3

| location=LmjF.34:1338350-1339381(+) | length=1032 | sequence_SO=chromosome

| SO=protein_coding_gene | is_pseudo=false

Length=1032

Score = 26.5 bits (28), Expect = 8.7

Identities = 14/14 (100%), Gaps = 0/14 (0%)

Strand=Plus/Plus

Query 34 GACGACGGTGCTGA 47

||||||||||||||

Sbjct 285 GACGACGGTGCTGA 298

>LmjF.34.1540:mRNA | gene=LmjF.34.1540 | organism=Leishmania_major_strain_Friedlin

| gene_product=kinesin, putative | transcript_product=kinesin,

putative | location=LmjF.34:693615-696812(-)

| length=3198 | sequence_SO=chromosome | SO=protein_coding_gene

| is_pseudo=false

Length=3198

Score = 26.5 bits (28), Expect = 8.7

Identities = 14/14 (100%), Gaps = 0/14 (0%)

Strand=Plus/Plus

Query 34 GACGACGGTGCTGA 47

||||||||||||||

Sbjct 1491 GACGACGGTGCTGA 1504

>LmjF.33.2590:mRNA | gene=LmjF.33.2590 | organism=Leishmania_major_strain_Friedlin

| gene_product=hypothetical protein, conserved

| transcript_product=hypothetical protein, conserved

| location=LmjF.33:1163929-1167102(+) | length=3174 | sequence_SO=chromosome

| SO=protein_coding_gene | is_pseudo=false

Length=3174

Score = 26.5 bits (28), Expect = 8.7

Identities = 26/34 (76%), Gaps = 0/34 (0%)

Strand=Plus/Plus

Query 4 TGTGCTGCTGGGGCACAACGGCGCCGGCAAGACG 37

|||| ||| | || || | ||||||||||| ||

Sbjct 2724 TGTGGTGCCGCAGCTCAGCAGCGCCGGCAAGGCG 2757

>LmjF.33.2180:mRNA | gene=LmjF.33.2180 | organism=Leishmania_major_strain_Friedlin

| gene_product=hypothetical protein, conserved

| transcript_product=hypothetical protein, conserved

| location=LmjF.33:984151-988383(+) | length=4233 | sequence_SO=chromosome

| SO=protein_coding_gene | is_pseudo=false

Length=4233

Score = 26.5 bits (28), Expect = 8.7

Identities = 14/14 (100%), Gaps = 0/14 (0%)

Strand=Plus/Minus

Query 4 TGTGCTGCTGGGGC 17

||||||||||||||

Sbjct 388 TGTGCTGCTGGGGC 375

>LmjF.33.2170:mRNA | gene=LmjF.33.2170 | organism=Leishmania_major_strain_Friedlin

| gene_product=hypothetical protein, conserved

| transcript_product=hypothetical protein, conserved

| location=LmjF.33:976079-980509(+) | length=4431 | sequence_SO=chromosome

| SO=protein_coding_gene | is_pseudo=false

Length=4431

Score = 26.5 bits (28), Expect = 8.7

Identities = 14/14 (100%), Gaps = 0/14 (0%)

Strand=Plus/Plus

Query 13 GGGGCACAACGGCG 26

||||||||||||||

Sbjct 3951 GGGGCACAACGGCG 3964

>LmjF.33.1090:mRNA | gene=LmjF.33.1090 | organism=Leishmania_major_strain_Friedlin

| gene_product=guanylate kinase, putative

| transcript_product=guanylate kinase, putative | location=LmjF.33:506559-507170(+)

| length=612 | sequence_SO=chromosome

| SO=protein_coding_gene | is_pseudo=false

Length=612

Score = 26.5 bits (28), Expect = 8.7

Identities = 14/14 (100%), Gaps = 0/14 (0%)

Strand=Plus/Plus

Query 1 CTCTGTGCTGCTGG 14

||||||||||||||

Sbjct 507 CTCTGTGCTGCTGG 520

>LmjF.32.3930:mRNA | gene=LmjF.32.3930 | organism=Leishmania_major_strain_Friedlin

| gene_product=SPRY domain/HECT-domain

(ubiquitin-transferase), putative | transcript_product=SPRY

domain/HECT-domain (ubiquitin-transferase), putative | location=LmjF.32:1569957-1582403(+)

| length=12447 | sequence_SO=chromosome

| SO=protein_coding_gene | is_pseudo=false

Length=12447

Score = 26.5 bits (28), Expect = 8.7

Identities = 14/14 (100%), Gaps = 0/14 (0%)

Strand=Plus/Minus

Query 17 CACAACGGCGCCGG 30

||||||||||||||

Sbjct 10719 CACAACGGCGCCGG 10706

>LmjF.32.2800:mRNA | gene=LmjF.32.2800 | organism=Leishmania_major_strain_Friedlin

| gene_product=hypothetical protein, conserved

| transcript_product=hypothetical protein, conserved

| location=LmjF.32:1099731-1103822(-) | length=4092 | sequence_SO=chromosome

| SO=protein_coding_gene | is_pseudo=false

Length=4092

Score = 26.5 bits (28), Expect = 8.7

Identities = 14/14 (100%), Gaps = 0/14 (0%)

Strand=Plus/Plus

Query 54 TGACGGGGATGGTC 67

||||||||||||||

Sbjct 1347 TGACGGGGATGGTC 1360

>LmjF.32.1720:mRNA | gene=LmjF.32.1720 | organism=Leishmania_major_strain_Friedlin

| gene_product=DNA polymerase alpha/epsilon

subunit B, putative | transcript_product=DNA polymerase

alpha/epsilon subunit B, putative | location=LmjF.32:672322-674502(-)

| length=2181 | sequence_SO=chromosome | SO=protein_coding_gene

| is_pseudo=false

Length=2181

Score = 26.5 bits (28), Expect = 8.7

Identities = 14/14 (100%), Gaps = 0/14 (0%)

Strand=Plus/Minus

Query 19 CAACGGCGCCGGCA 32

||||||||||||||

Sbjct 973 CAACGGCGCCGGCA 960

>LmjF.32.0190:mRNA | gene=LmjF.32.0190 | organism=Leishmania_major_strain_Friedlin

| gene_product=hypothetical protein, conserved

| transcript_product=hypothetical protein, conserved

| location=LmjF.32:59600-61906(-) | length=2307 | sequence_SO=chromosome

| SO=protein_coding_gene | is_pseudo=false

Length=2307

Score = 26.5 bits (28), Expect = 8.7

Identities = 14/14 (100%), Gaps = 0/14 (0%)

Strand=Plus/Minus

Query 16 GCACAACGGCGCCG 29

||||||||||||||

Sbjct 724 GCACAACGGCGCCG 711

>LmjF.31.1720:mRNA | gene=LmjF.31.1720 | organism=Leishmania_major_strain_Friedlin

| gene_product=Cullin protein neddylation

domain containing protein, putative | transcript_product=Cullin

protein neddylation domain containing protein, putative

| location=LmjF.31:800144-803740(-) | length=3597 | sequence_SO=chromosome

| SO=protein_coding_gene | is_pseudo=false

Length=3597

Score = 26.5 bits (28), Expect = 8.7

Identities = 14/14 (100%), Gaps = 0/14 (0%)

Strand=Plus/Plus

Query 36 CGACGGTGCTGAAC 49

||||||||||||||

Sbjct 1349 CGACGGTGCTGAAC 1362

>LmjF.31.0500:mRNA | gene=LmjF.31.0500 | organism=Leishmania_major_strain_Friedlin

| gene_product=hypothetical protein, conserved

| transcript_product=hypothetical protein, conserved

| location=LmjF.31:171934-173856(-) | length=1923 | sequence_SO=chromosome

| SO=protein_coding_gene | is_pseudo=false

Length=1923

Score = 26.5 bits (28), Expect = 8.7

Identities = 14/14 (100%), Gaps = 0/14 (0%)

Strand=Plus/Plus

Query 19 CAACGGCGCCGGCA 32

||||||||||||||

Sbjct 804 CAACGGCGCCGGCA 817

>LmjF.30.1810:mRNA | gene=LmjF.30.1810 | organism=Leishmania_major_strain_Friedlin

| gene_product=Zeta toxin, putative | transcript_product=Zeta

toxin, putative | location=LmjF.30:661454-678139(+)

| length=16686 | sequence_SO=chromosome | SO=protein_coding_gene

| is_pseudo=false

Length=16686

Score = 26.5 bits (28), Expect = 8.7

Identities = 17/19 (89%), Gaps = 0/19 (0%)

Strand=Plus/Minus

Query 22 CGGCGCCGGCAAGACGACG 40

||||||||||| |||| ||

Sbjct 12471 CGGCGCCGGCACGACGGCG 12453

>LmjF.29.2240:mRNA | gene=LmjF.29.2240 | organism=Leishmania_major_strain_Friedlin

| gene_product=Aminopeptidase M1, putative

| transcript_product=Aminopeptidase M1, putative | location=LmjF.29:987764-990370(-)

| length=2607 | sequence_SO=chromosome

| SO=protein_coding_gene | is_pseudo=false

Length=2607

Score = 26.5 bits (28), Expect = 8.7

Identities = 14/14 (100%), Gaps = 0/14 (0%)

Strand=Plus/Minus

Query 34 GACGACGGTGCTGA 47

||||||||||||||

Sbjct 630 GACGACGGTGCTGA 617

>LmjF.28.2580:mRNA | gene=LmjF.28.2580 | organism=Leishmania_major_strain_Friedlin

| gene_product=leucine-rich repeat protein,

putative | transcript_product=leucine-rich repeat protein,

putative | location=LmjF.28:1002695-1005352(+) | length=2658

| sequence_SO=chromosome | SO=protein_coding_gene | is_pseudo=false

Length=2658

Score = 26.5 bits (28), Expect = 8.7

Identities = 14/14 (100%), Gaps = 0/14 (0%)

Strand=Plus/Plus

Query 40 GGTGCTGAACATGA 53

||||||||||||||

Sbjct 606 GGTGCTGAACATGA 619

>LmjF.27.2590:mRNA | gene=LmjF.27.2590 | organism=Leishmania_major_strain_Friedlin

| gene_product=dynein heavy chain, putative

| transcript_product=dynein heavy chain, putative | location=LmjF.27:1093720-1106736(+)

| length=13017 | sequence_SO=chromosome

| SO=protein_coding_gene | is_pseudo=false

Length=13017

Score = 26.5 bits (28), Expect = 8.7

Identities = 14/14 (100%), Gaps = 0/14 (0%)

Strand=Plus/Minus

Query 18 ACAACGGCGCCGGC 31

||||||||||||||

Sbjct 286 ACAACGGCGCCGGC 273

>LmjF.26.1900:mRNA | gene=LmjF.26.1900 | organism=Leishmania_major_strain_Friedlin

| gene_product=hypothetical protein, conserved

| transcript_product=hypothetical protein, conserved

| location=LmjF.26:740256-743924(+) | length=3669 | sequence_SO=chromosome

| SO=protein_coding_gene | is_pseudo=false

Length=3669

Score = 26.5 bits (28), Expect = 8.7

Identities = 14/14 (100%), Gaps = 0/14 (0%)

Strand=Plus/Minus

Query 23 GGCGCCGGCAAGAC 36

||||||||||||||

Sbjct 297 GGCGCCGGCAAGAC 284

>LmjF.26.0720:mRNA | gene=LmjF.26.0720 | organism=Leishmania_major_strain_Friedlin

| gene_product=hypothetical protein, conserved

| transcript_product=hypothetical protein, conserved

| location=LmjF.26:200532-203801(-) | length=3270 | sequence_SO=chromosome

| SO=protein_coding_gene | is_pseudo=false

Length=3270

Score = 26.5 bits (28), Expect = 8.7

Identities = 14/14 (100%), Gaps = 0/14 (0%)

Strand=Plus/Minus

Query 34 GACGACGGTGCTGA 47

||||||||||||||

Sbjct 3101 GACGACGGTGCTGA 3088

>LmjF.26.0550:mRNA | gene=LmjF.26.0550 | organism=Leishmania_major_strain_Friedlin

| gene_product=galactofuranosyltransferase

lpg1-like protein | transcript_product=galactofuranosyltransferase

lpg1-like protein | location=LmjF.26:150195-151973(-)

| length=1779 | sequence_SO=chromosome | SO=protein_coding_gene

| is_pseudo=false

Length=1779

Score = 26.5 bits (28), Expect = 8.7

Identities = 17/19 (89%), Gaps = 0/19 (0%)

Strand=Plus/Plus

Query 23 GGCGCCGGCAAGACGACGG 41

|||||| |||||||||||

Sbjct 931 GGCGCCTACAAGACGACGG 949

>LmjF.25.1950:mRNA | gene=LmjF.25.1950 | organism=Leishmania_major_strain_Friedlin

| gene_product=kinesin, putative | transcript_product=kinesin,

putative | location=LmjF.25:758553-761741(-)

| length=3189 | sequence_SO=chromosome | SO=protein_coding_gene

| is_pseudo=false

Length=3189

Score = 26.5 bits (28), Expect = 8.7

Identities = 20/24 (83%), Gaps = 0/24 (0%)

Strand=Plus/Plus

Query 14 GGGCACAACGGCGCCGGCAAGACG 37

|| || | |||| |||||||||||

Sbjct 229 GGCCAGACCGGCACCGGCAAGACG 252

>LmjF.25.0980:mRNA | gene=LmjF.25.0980 | organism=Leishmania_major_strain_Friedlin

| gene_product=dynein heavy chain, putative

| transcript_product=dynein heavy chain, putative | location=LmjF.25:367262-381319(+)

| length=14058 | sequence_SO=chromosome

| SO=protein_coding_gene | is_pseudo=false

Length=14058

Score = 26.5 bits (28), Expect = 8.7

Identities = 14/14 (100%), Gaps = 0/14 (0%)

Strand=Plus/Plus

Query 28 CGGCAAGACGACGG 41

||||||||||||||

Sbjct 1584 CGGCAAGACGACGG 1597

>LmjF.25.0830:mRNA | gene=LmjF.25.0830 | organism=Leishmania_major_strain_Friedlin

| gene_product=hypothetical protein, conserved

| transcript_product=hypothetical protein, conserved

| location=LmjF.25:314250-317447(+) | length=3198 | sequence_SO=chromosome

| SO=protein_coding_gene | is_pseudo=false

Length=3198

Score = 26.5 bits (28), Expect = 8.7

Identities = 17/19 (89%), Gaps = 0/19 (0%)

Strand=Plus/Plus

Query 14 GGGCACAACGGCGCCGGCA 32

|| |||||||||||| |||

Sbjct 1747 GGACACAACGGCGCCCGCA 1765

>LmjF.23.0543:mRNA | gene=LmjF.23.0543 | organism=Leishmania_major_strain_Friedlin

| gene_product=5'-3' exoribonuclease C,

putative | transcript_product=5'-3' exoribonuclease C, putative

| location=LmjF.23:202387-205593(+) | length=3207 | sequence_SO=chromosome

| SO=protein_coding_gene | is_pseudo=false

Length=3207

Score = 26.5 bits (28), Expect = 8.7

Identities = 17/19 (89%), Gaps = 0/19 (0%)

Strand=Plus/Plus

Query 8 CTGCTGGGGCACAACGGCG 26

|||| || |||||||||||

Sbjct 1453 CTGCAGGTGCACAACGGCG 1471

>LmjF.23.0440:mRNA | gene=LmjF.23.0440 | organism=Leishmania_major_strain_Friedlin

| gene_product=hypothetical protein, conserved

| transcript_product=hypothetical protein, conserved

| location=LmjF.23:162691-163644(+) | length=954 | sequence_SO=chromosome

| SO=protein_coding_gene | is_pseudo=false

Length=954

Score = 26.5 bits (28), Expect = 8.7

Identities = 14/14 (100%), Gaps = 0/14 (0%)

Strand=Plus/Plus

Query 21 ACGGCGCCGGCAAG 34

||||||||||||||

Sbjct 247 ACGGCGCCGGCAAG 260

>LmjF.22.1620:mRNA | gene=LmjF.22.1620 | organism=Leishmania_major_strain_Friedlin

| gene_product=hypothetical protein, conserved

| transcript_product=hypothetical protein, conserved

| location=LmjF.22:663375-671780(+) | length=8406 | sequence_SO=chromosome

| SO=protein_coding_gene | is_pseudo=false

Length=8406

Score = 26.5 bits (28), Expect = 8.7

Identities = 14/14 (100%), Gaps = 0/14 (0%)

Strand=Plus/Plus

Query 33 AGACGACGGTGCTG 46

||||||||||||||

Sbjct 3653 AGACGACGGTGCTG 3666

>LmjF.21.1340:mRNA | gene=LmjF.21.1340 | organism=Leishmania_major_strain_Friedlin

| gene_product=ATP synthase, putative |

transcript_product=ATP synthase, putative | location=LmjF.21:555322-556773(-)

| length=1452 | sequence_SO=chromosome |

SO=protein_coding_gene | is_pseudo=false

Length=1452

Score = 26.5 bits (28), Expect = 8.7

Identities = 17/19 (89%), Gaps = 0/19 (0%)

Strand=Plus/Plus

Query 37 GACGGTGCTGAACATGATG 55

|||| ||||||||||| ||

Sbjct 783 GACGCTGCTGAACATGGTG 801

>LmjF.20.1530:mRNA | gene=LmjF.20.1530 | organism=Leishmania_major_strain_Friedlin

| gene_product=cell division cycle protein-like

protein | transcript_product=cell division cycle protein-like

protein | location=LmjF.20:686922-689366(-) | length=2445

| sequence_SO=chromosome | SO=protein_coding_gene |

is_pseudo=false

Length=2445

Score = 26.5 bits (28), Expect = 8.7

Identities = 20/24 (83%), Gaps = 0/24 (0%)

Strand=Plus/Plus

Query 14 GGGCACAACGGCGCCGGCAAGACG 37

|||| || ||| |||||||||||

Sbjct 1606 GGGCCCAGCGGATCCGGCAAGACG 1629

>LmjF.15.1320:mRNA | gene=LmjF.15.1320 | organism=Leishmania_major_strain_Friedlin

| gene_product=Eukaryotic translation initiation

factor 4 gamma 2 | transcript_product=Eukaryotic translation

initiation factor 4 gamma 2 | location=LmjF.15:549203-553480(-)

| length=4278 | sequence_SO=chromosome | SO=protein_coding_gene

| is_pseudo=false

Length=4278

Score = 26.5 bits (28), Expect = 8.7

Identities = 17/19 (89%), Gaps = 0/19 (0%)

Strand=Plus/Plus

Query 39 CGGTGCTGAACATGATGAC 57

|||||||||||| | ||||

Sbjct 1358 CGGTGCTGAACAAGGTGAC 1376

>LmjF.15.0595:pseudogenic_transcript | gene=LmjF.15.0595 | organism=Leishmania_major_strain_Friedlin

| gene_product=hypothetical

protein | transcript_product=hypothetical protein | location=LmjF.15:243584-244078(+)

| length=4034 | sequence_SO=chromosome

| SO=pseudogene | is_pseudo=true

Length=4034

Score = 26.5 bits (28), Expect = 8.7

Identities = 20/24 (83%), Gaps = 0/24 (0%)

Strand=Plus/Minus

Query 17 CACAACGGCGCCGGCAAGACGACG 40

|||||||||||| | |||||||

Sbjct 3568 CACAACGGCGCCCTAACGACGACG 3545

>LmjF.12.0640:mRNA | gene=LmjF.12.0640 | organism=Leishmania_major_strain_Friedlin

| gene_product=primase 2, putative | transcript_product=primase

2, putative | location=LmjF.12:346619-348862(+)

| length=2244 | sequence_SO=chromosome | SO=protein_coding_gene

| is_pseudo=false

Length=2244

Score = 26.5 bits (28), Expect = 8.7

Identities = 14/14 (100%), Gaps = 0/14 (0%)

Strand=Plus/Plus

Query 51 TGATGACGGGGATG 64

||||||||||||||

Sbjct 447 TGATGACGGGGATG 460

>LmjF.09.0390:mRNA | gene=LmjF.09.0390 | organism=Leishmania_major_strain_Friedlin

| gene_product=hypothetical protein, conserved

| transcript_product=hypothetical protein, conserved

| location=LmjF.09:156941-162871(+) | length=5931 | sequence_SO=chromosome

| SO=protein_coding_gene | is_pseudo=false

Length=5931

Score = 26.5 bits (28), Expect = 8.7

Identities = 14/14 (100%), Gaps = 0/14 (0%)

Strand=Plus/Plus

Query 21 ACGGCGCCGGCAAG 34

||||||||||||||

Sbjct 4774 ACGGCGCCGGCAAG 4787

>LmjF.08.1080:mRNA | gene=LmjF.08.1080 | organism=Leishmania_major_strain_Friedlin

| gene_product=cathepsin L-like protease

| transcript_product=cathepsin L-like protease | location=LmjF.08:477996-479693(-)

| length=1698 | sequence_SO=chromosome

| SO=protein_coding_gene | is_pseudo=false

Length=1698

Score = 26.5 bits (28), Expect = 8.7

Identities = 14/14 (100%), Gaps = 0/14 (0%)

Strand=Plus/Plus

Query 30 GCAAGACGACGGTG 43

||||||||||||||

Sbjct 1493 GCAAGACGACGGTG 1506

Lambda K H

0.634 0.408 0.912

Gapped

Lambda K H

0.625 0.410 0.780

Effective search space used: 840868008

Database: /eupath/data/apiSiteFilesStaging/TriTrypDB/52/real/webServices/TriTr

ypDB/release-

CURRENT/LmajorFriedlin/blast/LmajorFriedlinAnnotatedTranscripts

Posted date: Dec 16, 2021 1:23 PM

Number of letters in database: 16,705,993

Number of sequences in database: 9,495

Matrix: blastn matrix 2 -3

Gap Penalties: Existence: 5, Extension: 2

__________________________________________________________________________________________

PART 2

BLASTN 2.10.1+

Reference: Stephen F. Altschul, Thomas L. Madden, Alejandro A.

Schaffer, Jinghui Zhang, Zheng Zhang, Webb Miller, and David J.

Lipman (1997), "Gapped BLAST and PSI-BLAST: a new generation of

protein database search programs", Nucleic Acids Res. 25:3389-3402.

Database: /eupath/data/apiSiteFilesStaging/TriTrypDB/52/real/webServices/TriTr

ypDB/release-

CURRENT/LmajorFriedlin/blast/LmajorFriedlinAnnotatedTranscripts

9,495 sequences; 16,705,993 total letters

Query= 11

Length=73

Score E

Sequences producing significant alignments: (Bits) Value

LmjF.11.1260:pseudogenic_transcript | gene=LmjF.11.1260 | organis... 128 3e-30

LmjF.11.1240:mRNA | gene=LmjF.11.1240 | organism=Leishmania_major... 128 3e-30

LmjF.11.1230:pseudogenic_transcript | gene=LmjF.11.1230 | organis... 123 4e-29

LmjF.27.0980:mRNA | gene=LmjF.27.0980 | organism=Leishmania_major... 68.9 3e-12

LmjF.27.0970:mRNA | gene=LmjF.27.0970 | organism=Leishmania_major... 68.9 3e-12

LmjF.11.1270:mRNA | gene=LmjF.11.1270 | organism=Leishmania_major... 65.3 3e-11

LmjF.11.1250:mRNA | gene=LmjF.11.1250 | organism=Leishmania_major... 65.3 3e-11

LmjF.11.1220:mRNA | gene=LmjF.11.1220 | organism=Leishmania_major... 65.3 3e-11

LmjF.11.1290:mRNA | gene=LmjF.11.1290 | organism=Leishmania_major... 60.8 4e-10

LmjF.30.0900:mRNA | gene=LmjF.30.0900 | organism=Leishmania_major... 31.9 0.20

LmjF.25.1500:mRNA | gene=LmjF.25.1500 | organism=Leishmania_major... 31.9 0.20

LmjF.32.3450:mRNA | gene=LmjF.32.3450 | organism=Leishmania_major... 31.0 0.70

LmjF.36.6860:mRNA | gene=LmjF.36.6860 | organism=Leishmania_major... 30.1 0.70

LmjF.35.0910:mRNA | gene=LmjF.35.0910 | organism=Leishmania_major... 30.1 0.70

LmjF.21.0840:mRNA | gene=LmjF.21.0840 | organism=Leishmania_major... 30.1 0.70

LmjF.11.0180:mRNA | gene=LmjF.11.0180 | organism=Leishmania_major... 30.1 0.70

LmjF.04.0550:mRNA | gene=LmjF.04.0550 | organism=Leishmania_major... 30.1 0.70

LmjF.03.0520:mRNA | gene=LmjF.03.0520 | organism=Leishmania_major... 30.1 0.70

LmjF.31.0400:mRNA | gene=LmjF.31.0400 | organism=Leishmania_major... 29.2 2.4

LmjF.18.1350:mRNA | gene=LmjF.18.1350 | organism=Leishmania_major... 29.2 2.4

LmjF.09.1210:mRNA | gene=LmjF.09.1210 | organism=Leishmania_major... 29.2 2.4

LmjF.02.0410:mRNA | gene=LmjF.02.0410 | organism=Leishmania_major... 29.2 2.4

LmjF.36.1280:mRNA | gene=LmjF.36.1280 | organism=Leishmania_major... 28.3 2.4

LmjF.35.1700:mRNA | gene=LmjF.35.1700 | organism=Leishmania_major... 28.3 2.4

LmjF.34.1970:mRNA | gene=LmjF.34.1970 | organism=Leishmania_major... 28.3 2.4

LmjF.34.1950:mRNA | gene=LmjF.34.1950 | organism=Leishmania_major... 28.3 2.4

LmjF.34.1930:mRNA | gene=LmjF.34.1930 | organism=Leishmania_major... 28.3 2.4

LmjF.34.1910:mRNA | gene=LmjF.34.1910 | organism=Leishmania_major... 28.3 2.4

LmjF.34.1890:mRNA | gene=LmjF.34.1890 | organism=Leishmania_major... 28.3 2.4

LmjF.34.1870:mRNA | gene=LmjF.34.1870 | organism=Leishmania_major... 28.3 2.4

LmjF.34.1850:mRNA | gene=LmjF.34.1850 | organism=Leishmania_major... 28.3 2.4

LmjF.34.1830:mRNA | gene=LmjF.34.1830 | organism=Leishmania_major... 28.3 2.4

LmjF.34.1810:mRNA | gene=LmjF.34.1810 | organism=Leishmania_major... 28.3 2.4

LmjF.34.1790:mRNA | gene=LmjF.34.1790 | organism=Leishmania_major... 28.3 2.4

LmjF.34.1770:mRNA | gene=LmjF.34.1770 | organism=Leishmania_major... 28.3 2.4

LmjF.34.1750:mRNA | gene=LmjF.34.1750 | organism=Leishmania_major... 28.3 2.4

LmjF.34.1730:mRNA | gene=LmjF.34.1730 | organism=Leishmania_major... 28.3 2.4

LmjF.34.1710:mRNA | gene=LmjF.34.1710 | organism=Leishmania_major... 28.3 2.4

LmjF.34.1690:mRNA | gene=LmjF.34.1690 | organism=Leishmania_major... 28.3 2.4

LmjF.34.1670:mRNA | gene=LmjF.34.1670 | organism=Leishmania_major... 28.3 2.4

LmjF.34.1650:mRNA | gene=LmjF.34.1650 | organism=Leishmania_major... 28.3 2.4

LmjF.34.1630:mRNA | gene=LmjF.34.1630 | organism=Leishmania_major... 28.3 2.4

LmjF.34.1610:mRNA | gene=LmjF.34.1610 | organism=Leishmania_major... 28.3 2.4

LmjF.34.1590:mRNA | gene=LmjF.34.1590 | organism=Leishmania_major... 28.3 2.4

LmjF.34.1570:mRNA | gene=LmjF.34.1570 | organism=Leishmania_major... 28.3 2.4

LmjF.34.0550:mRNA | gene=LmjF.34.0550 | organism=Leishmania_major... 28.3 2.4

LmjF.33.1730:mRNA | gene=LmjF.33.1730 | organism=Leishmania_major... 28.3 2.4

LmjF.31.0860:mRNA | gene=LmjF.31.0860 | organism=Leishmania_major... 28.3 2.4

LmjF.31.0810:mRNA | gene=LmjF.31.0810 | organism=Leishmania_major... 28.3 2.4

LmjF.28.0990:mRNA | gene=LmjF.28.0990 | organism=Leishmania_major... 28.3 2.4

LmjF.27.0500:mRNA | gene=LmjF.27.0500 | organism=Leishmania_major... 28.3 2.4

LmjF.26.1330:mRNA | gene=LmjF.26.1330 | organism=Leishmania_major... 28.3 2.4

LmjF.25.1570:mRNA | gene=LmjF.25.1570 | organism=Leishmania_major... 28.3 2.4

LmjF.25.0680:mRNA | gene=LmjF.25.0680 | organism=Leishmania_major... 28.3 2.4

LmjF.24.2090:mRNA | gene=LmjF.24.2090 | organism=Leishmania_major... 28.3 2.4

LmjF.14.1450:mRNA | gene=LmjF.14.1450 | organism=Leishmania_major... 28.3 2.4

LmjF.14.0340:mRNA | gene=LmjF.14.0340 | organism=Leishmania_major... 28.3 2.4

LmjF.13.0240.2:mRNA | gene=LmjF.13.0240 | organism=Leishmania_maj... 28.3 2.4

LmjF.13.0240.1:mRNA | gene=LmjF.13.0240 | organism=Leishmania_maj... 28.3 2.4

LmjF.12.0650:mRNA | gene=LmjF.12.0650 | organism=Leishmania_major... 28.3 2.4

LmjF.09.0990:mRNA | gene=LmjF.09.0990 | organism=Leishmania_major... 28.3 2.4

LmjF.36.5750:mRNA | gene=LmjF.36.5750 | organism=Leishmania_major... 27.4 8.5

LmjF.36.3960:mRNA | gene=LmjF.36.3960 | organism=Leishmania_major... 27.4 8.5

LmjF.36.1370:mRNA | gene=LmjF.36.1370 | organism=Leishmania_major... 27.4 8.5

LmjF.34.4620:mRNA | gene=LmjF.34.4620 | organism=Leishmania_major... 27.4 8.5

LmjF.19.1150:mRNA | gene=LmjF.19.1150 | organism=Leishmania_major... 27.4 8.5

LmjF.18.1140:mRNA | gene=LmjF.18.1140 | organism=Leishmania_major... 27.4 8.5

LmjF.14.1110:mRNA | gene=LmjF.14.1110 | organism=Leishmania_major... 27.4 8.5

LmjF.12.1120:mRNA | gene=LmjF.12.1120 | organism=Leishmania_major... 27.4 8.5

LmjF.09.0760:mRNA | gene=LmjF.09.0760 | organism=Leishmania_major... 27.4 8.5

LmjF.06.0320:mRNA | gene=LmjF.06.0320 | organism=Leishmania_major... 27.4 8.5

LmjF.03.0360:mRNA | gene=LmjF.03.0360 | organism=Leishmania_major... 27.4 8.5

LmjF.36.6430:mRNA | gene=LmjF.36.6430 | organism=Leishmania_major... 26.5 8.5

LmjF.36.5530:mRNA | gene=LmjF.36.5530 | organism=Leishmania_major... 26.5 8.5

LmjF.36.5380:mRNA | gene=LmjF.36.5380 | organism=Leishmania_major... 26.5 8.5

LmjF.36.4520:mRNA | gene=LmjF.36.4520 | organism=Leishmania_major... 26.5 8.5

LmjF.36.0850:mRNA | gene=LmjF.36.0850 | organism=Leishmania_major... 26.5 8.5

LmjF.34.3200:mRNA | gene=LmjF.34.3200 | organism=Leishmania_major... 26.5 8.5

LmjF.34.0660:mRNA | gene=LmjF.34.0660 | organism=Leishmania_major... 26.5 8.5

LmjF.31.0190:mRNA | gene=LmjF.31.0190 | organism=Leishmania_major... 26.5 8.5

LmjF.30.0940:mRNA | gene=LmjF.30.0940 | organism=Leishmania_major... 26.5 8.5

LmjF.29.0790:mRNA | gene=LmjF.29.0790 | organism=Leishmania_major... 26.5 8.5

LmjF.27.0370:mRNA | gene=LmjF.27.0370 | organism=Leishmania_major... 26.5 8.5

LmjF.25.1110:mRNA | gene=LmjF.25.1110 | organism=Leishmania_major... 26.5 8.5

LmjF.25.0870:mRNA | gene=LmjF.25.0870 | organism=Leishmania_major... 26.5 8.5

LmjF.24.1280:mRNA | gene=LmjF.24.1280 | organism=Leishmania_major... 26.5 8.5

LmjF.24.1250:mRNA | gene=LmjF.24.1250 | organism=Leishmania_major... 26.5 8.5

LmjF.23.1510:mRNA | gene=LmjF.23.1510 | organism=Leishmania_major... 26.5 8.5

LmjF.20.0290:mRNA | gene=LmjF.20.0290 | organism=Leishmania_major... 26.5 8.5

LmjF.16.1190:mRNA | gene=LmjF.16.1190 | organism=Leishmania_major... 26.5 8.5

LmjF.16.1120:mRNA | gene=LmjF.16.1120 | organism=Leishmania_major... 26.5 8.5

LmjF.13.1650:mRNA | gene=LmjF.13.1650 | organism=Leishmania_major... 26.5 8.5

LmjF.13.1600:mRNA | gene=LmjF.13.1600 | organism=Leishmania_major... 26.5 8.5

LmjF.07.0480:mRNA | gene=LmjF.07.0480 | organism=Leishmania_major... 26.5 8.5

LmjF.05.0870:mRNA | gene=LmjF.05.0870 | organism=Leishmania_major... 26.5 8.5

LmjF.04.0980:mRNA | gene=LmjF.04.0980 | organism=Leishmania_major... 26.5 8.5

>LmjF.11.1260:pseudogenic_transcript | gene=LmjF.11.1260 | organism=Leishmania_major_strain_Friedlin

| gene_product=ABC transporter-like

protein | transcript_product=ABC transporter-like

protein | location=LmjF.11:528430-529703(+) | length=1272

| sequence_SO=chromosome | SO=pseudogene | is_pseudo=true

Length=1272

Score = 128 bits (141), Expect = 3e-30

Identities = 72/73 (99%), Gaps = 0/73 (0%)

Strand=Plus/Plus

Query 1 GGAGGAGGGGGAGGCGCTTGCGGACATTGTGGCGATCATGGTGCGCGGGTACGTGCGGTG 60

|||||||| |||||||||||||||||||||||||||||||||||||||||||||||||||

Sbjct 873 GGAGGAGGTGGAGGCGCTTGCGGACATTGTGGCGATCATGGTGCGCGGGTACGTGCGGTG 932

Query 61 TGTCGGCGACAAG 73

|||||||||||||

Sbjct 933 TGTCGGCGACAAG 945

>LmjF.11.1240:mRNA | gene=LmjF.11.1240 | organism=Leishmania_major_strain_Friedlin

| gene_product=ATP-binding cassette protein

subfamily A, member 3, putative | transcript_product=ATP-binding

cassette protein subfamily A, member 3, putative |

location=LmjF.11:510352-516078(+) | length=5727 | sequence_SO=chromosome

| SO=protein_coding_gene | is_pseudo=false

Length=5727

Score = 128 bits (141), Expect = 3e-30

Identities = 72/73 (99%), Gaps = 0/73 (0%)

Strand=Plus/Plus

Query 1 GGAGGAGGGGGAGGCGCTTGCGGACATTGTGGCGATCATGGTGCGCGGGTACGTGCGGTG 60

|||||||| |||||||||||||||||||||||||||||||||||||||||||||||||||

Sbjct 5358 GGAGGAGGTGGAGGCGCTTGCGGACATTGTGGCGATCATGGTGCGCGGGTACGTGCGGTG 5417

Query 61 TGTCGGCGACAAG 73

|||||||||||||

Sbjct 5418 TGTCGGCGACAAG 5430

>LmjF.11.1230:pseudogenic_transcript | gene=LmjF.11.1230 | organism=Leishmania_major_strain_Friedlin

| gene_product=ABC transporter-like

protein | transcript_product=ABC transporter-like

protein | location=LmjF.11:507789-508810(+) | length=1020

| sequence_SO=chromosome | SO=pseudogene | is_pseudo=true

Length=1020

Score = 123 bits (136), Expect = 4e-29

Identities = 71/73 (97%), Gaps = 0/73 (0%)

Strand=Plus/Plus

Query 1 GGAGGAGGGGGAGGCGCTTGCGGACATTGTGGCGATCATGGTGCGCGGGTACGTGCGGTG 60

|||||||| ||||||||||||| |||||||||||||||||||||||||||||||||||||

Sbjct 651 GGAGGAGGTGGAGGCGCTTGCGAACATTGTGGCGATCATGGTGCGCGGGTACGTGCGGTG 710

Query 61 TGTCGGCGACAAG 73

|||||||||||||

Sbjct 711 TGTCGGCGACAAG 723

>LmjF.27.0980:mRNA | gene=LmjF.27.0980 | organism=Leishmania_major_strain_Friedlin

| gene_product=ATP-binding cassette protein

subfamily A, member 9, putative | transcript_product=ATP-binding

cassette protein subfamily A, member 9, putative |

location=LmjF.27:421573-427215(-) | length=5643 | sequence_SO=chromosome

| SO=protein_coding_gene | is_pseudo=false

Length=5643

Score = 68.9 bits (75), Expect = 3e-12

Identities = 61/74 (82%), Gaps = 2/74 (3%)

Strand=Plus/Plus

Query 1 GGAGGAGGGGGAGGCGCTTGCGGACATTGTGGCGATCATGGTGCGCGGGTACG-TGCGGT 59

|||||||| ||||||||||||| || || |||||||||||| |||| ||| ||||||

Sbjct 5214 GGAGGAGGTGGAGGCGCTTGCGCACCGCGTTGCGATCATGGTGGACGGG-ACGCTGCGGT 5272

Query 60 GTGTCGGCGACAAG 73

| | |||||||||

Sbjct 5273 GCATTGGCGACAAG 5286

>LmjF.27.0970:mRNA | gene=LmjF.27.0970 | organism=Leishmania_major_strain_Friedlin

| gene_product=ATP-binding cassette protein

subfamily A, member 8, putative | transcript_product=ATP-binding

cassette protein subfamily A, member 8, putative |

location=LmjF.27:410527-416061(-) | length=5535 | sequence_SO=chromosome

| SO=protein_coding_gene | is_pseudo=false

Length=5535

Score = 68.9 bits (75), Expect = 3e-12

Identities = 61/74 (82%), Gaps = 2/74 (3%)

Strand=Plus/Plus

Query 1 GGAGGAGGGGGAGGCGCTTGCGGACATTGTGGCGATCATGGTGCGCGGGTACG-TGCGGT 59

|||||||| ||||||||||||| || || |||||||||||| |||| ||| ||||||

Sbjct 5088 GGAGGAGGTGGAGGCGCTTGCGCACCGCGTTGCGATCATGGTGGACGGG-ACGCTGCGGT 5146

Query 60 GTGTCGGCGACAAG 73

| | |||||||||

Sbjct 5147 GCATTGGCGACAAG 5160

>LmjF.11.1270:mRNA | gene=LmjF.11.1270 | organism=Leishmania_major_strain_Friedlin

| gene_product=ATP-binding cassette protein

subfamily A, member 5, putative | transcript_product=ATP-binding

cassette protein subfamily A, member 5, putative |

location=LmjF.11:531133-536517(+) | length=5385 | sequence_SO=chromosome

| SO=protein_coding_gene | is_pseudo=false

Length=5385

Score = 65.3 bits (71), Expect = 3e-11

Identities = 58/73 (79%), Gaps = 0/73 (0%)

Strand=Plus/Plus

Query 1 GGAGGAGGGGGAGGCGCTTGCGGACATTGTGGCGATCATGGTGCGCGGGTACGTGCGGTG 60

|||||||| ||||||||||||| | ||||||||||||||| ||| | |||| ||

Sbjct 4872 GGAGGAGGTGGAGGCGCTTGCGCATCGCGTGGCGATCATGGTGGACGGCACCCTGCGCTG 4931

Query 61 TGTCGGCGACAAG 73

|||||||||||

Sbjct 4932 CATCGGCGACAAG 4944

>LmjF.11.1250:mRNA | gene=LmjF.11.1250 | organism=Leishmania_major_strain_Friedlin

| gene_product=ATP-binding cassette protein

subfamily A, member 4, putative | transcript_product=ATP-binding

cassette protein subfamily A, member 4, putative |

location=LmjF.11:520967-526297(+) | length=5331 | sequence_SO=chromosome

| SO=protein_coding_gene | is_pseudo=false

Length=5331

Score = 65.3 bits (71), Expect = 3e-11

Identities = 58/73 (79%), Gaps = 0/73 (0%)

Strand=Plus/Plus

Query 1 GGAGGAGGGGGAGGCGCTTGCGGACATTGTGGCGATCATGGTGCGCGGGTACGTGCGGTG 60

|||||||| ||||||||||||| | ||||||||||||||| ||| | |||| ||

Sbjct 4896 GGAGGAGGTGGAGGCGCTTGCGCATCGCGTGGCGATCATGGTGGACGGCACCCTGCGCTG 4955

Query 61 TGTCGGCGACAAG 73

|||||||||||

Sbjct 4956 CATCGGCGACAAG 4968

>LmjF.11.1220:mRNA | gene=LmjF.11.1220 | organism=Leishmania_major_strain_Friedlin

| gene_product=ATP-binding cassette protein

subfamily A, member 2, putative | transcript_product=ATP-binding

cassette protein subfamily A, member 2, putative |

location=LmjF.11:500104-505434(+) | length=5331 | sequence_SO=chromosome

| SO=protein_coding_gene | is_pseudo=false

Length=5331

Score = 65.3 bits (71), Expect = 3e-11

Identities = 58/73 (79%), Gaps = 0/73 (0%)

Strand=Plus/Plus

Query 1 GGAGGAGGGGGAGGCGCTTGCGGACATTGTGGCGATCATGGTGCGCGGGTACGTGCGGTG 60

|||||||| ||||||||||||| | ||||||||||||||| ||| | |||| ||

Sbjct 4896 GGAGGAGGTGGAGGCGCTTGCGCATCGCGTGGCGATCATGGTGGACGGCACCCTGCGCTG 4955

Query 61 TGTCGGCGACAAG 73

|||||||||||

Sbjct 4956 CATCGGCGACAAG 4968

>LmjF.11.1290:mRNA | gene=LmjF.11.1290 | organism=Leishmania_major_strain_Friedlin

| gene_product=ATP-binding cassette protein

subfamily A, member 6, putative | transcript_product=ATP-binding

cassette protein subfamily A, member 6, putative |

location=LmjF.11:545392-551310(+) | length=5919 | sequence_SO=chromosome

| SO=protein_coding_gene | is_pseudo=false

Length=5919

Score = 60.8 bits (66), Expect = 4e-10

Identities = 57/73 (78%), Gaps = 0/73 (0%)

Strand=Plus/Plus

Query 1 GGAGGAGGGGGAGGCGCTTGCGGACATTGTGGCGATCATGGTGCGCGGGTACGTGCGGTG 60

|||||||| ||||||||||||||||| |||| ||||||| ||| | |||| ||

Sbjct 4950 GGAGGAGGTGGAGGCGCTTGCGGACACGGTGGGCATCATGGCTGACGGAGCCCTGCGCTG 5009

Query 61 TGTCGGCGACAAG 73

|||||||||||

Sbjct 5010 CATCGGCGACAAG 5022

>LmjF.30.0900:mRNA | gene=LmjF.30.0900 | organism=Leishmania_major_strain_Friedlin

| gene_product=hypothetical protein, conserved

| transcript_product=hypothetical protein, conserved

| location=LmjF.30:292312-294384(+) | length=2073 | sequence_SO=chromosome

| SO=protein_coding_gene | is_pseudo=false

Length=2073

Score = 31.9 bits (34), Expect = 0.20

Identities = 20/22 (91%), Gaps = 0/22 (0%)

Strand=Plus/Plus

Query 32 GCGATCATGGTGCGCGGGTACG 53

|||||| ||||||||||| |||

Sbjct 349 GCGATCCTGGTGCGCGGGCACG 370

>LmjF.25.1500:mRNA | gene=LmjF.25.1500 | organism=Leishmania_major_strain_Friedlin

| gene_product=PUA domain/NOL1/NOP2/sun

family, putative | transcript_product=PUA domain/NOL1/NOP2/sun

family, putative | location=LmjF.25:600167-602293(-) | length=2127

| sequence_SO=chromosome | SO=protein_coding_gene

| is_pseudo=false

Length=2127

Score = 31.9 bits (34), Expect = 0.20

Identities = 17/17 (100%), Gaps = 0/17 (0%)

Strand=Plus/Plus

Query 51 ACGTGCGGTGTGTCGGC 67

|||||||||||||||||

Sbjct 209 ACGTGCGGTGTGTCGGC 225

>LmjF.32.3450:mRNA | gene=LmjF.32.3450 | organism=Leishmania_major_strain_Friedlin

| gene_product=zinc-finger of a C2HC-type,

putative | transcript_product=zinc-finger of a C2HC-type,

putative | location=LmjF.32:1389213-1391105(+) | length=1893

| sequence_SO=chromosome | SO=protein_coding_gene | is_pseudo=false

Length=1893

Score = 31.0 bits (33), Expect = 0.70

Identities = 18/19 (95%), Gaps = 0/19 (0%)

Strand=Plus/Minus

Query 2 GAGGAGGGGGAGGCGCTTG 20

|||| ||||||||||||||

Sbjct 779 GAGGCGGGGGAGGCGCTTG 761

>LmjF.36.6860:mRNA | gene=LmjF.36.6860 | organism=Leishmania_major_strain_Friedlin

| gene_product=calphotin-like protein |

transcript_product=calphotin-like protein | location=LmjF.36:2639956-2641860(-)

| length=1905 | sequence_SO=chromosome

| SO=protein_coding_gene | is_pseudo=false

Length=1905

Score = 30.1 bits (32), Expect = 0.70

Identities = 19/21 (90%), Gaps = 0/21 (0%)

Strand=Plus/Minus

Query 4 GGAGGGGGAGGCGCTTGCGGA 24

|||||||||||||| |||||

Sbjct 381 GGAGGGGGAGGCGCCAGCGGA 361

>LmjF.35.0910:mRNA | gene=LmjF.35.0910 | organism=Leishmania_major_strain_Friedlin

| gene_product=hypothetical protein, conserved

| transcript_product=hypothetical protein, conserved

| location=LmjF.35:406878-412871(+) | length=5994 | sequence_SO=chromosome

| SO=protein_coding_gene | is_pseudo=false

Length=5994

Score = 30.1 bits (32), Expect = 0.70

Identities = 16/16 (100%), Gaps = 0/16 (0%)

Strand=Plus/Minus

Query 1 GGAGGAGGGGGAGGCG 16

||||||||||||||||

Sbjct 567 GGAGGAGGGGGAGGCG 552

>LmjF.21.0840:mRNA | gene=LmjF.21.0840 | organism=Leishmania_major_strain_Friedlin

| gene_product=metallo-peptidase, Clan

MG, Family M24 | transcript_product=metallo-peptidase, Clan

MG, Family M24 | location=LmjF.21:307359-308756(+) | length=1398

| sequence_SO=chromosome | SO=protein_coding_gene | is_pseudo=false

Length=1398

Score = 30.1 bits (32), Expect = 0.70

Identities = 19/21 (90%), Gaps = 0/21 (0%)

Strand=Plus/Plus

Query 1 GGAGGAGGGGGAGGCGCTTGC 21

|||||||||||||| | ||||

Sbjct 1026 GGAGGAGGGGGAGGTGTTTGC 1046

>LmjF.11.0180:mRNA | gene=LmjF.11.0180 | organism=Leishmania_major_strain_Friedlin

| gene_product=cytoplasmic translation

machinery associated protein, putative | transcript_product=cytoplasmic

translation machinery associated protein, putative

| location=LmjF.11:48521-49111(+) | length=591 | sequence_SO=chromosome

| SO=protein_coding_gene | is_pseudo=false

Length=591

Score = 30.1 bits (32), Expect = 0.70

Identities = 19/21 (90%), Gaps = 0/21 (0%)

Strand=Plus/Plus

Query 1 GGAGGAGGGGGAGGCGCTTGC 21

|||||||||||||| | ||||

Sbjct 405 GGAGGAGGGGGAGGTGGTTGC 425

>LmjF.04.0550:mRNA | gene=LmjF.04.0550 | organism=Leishmania_major_strain_Friedlin

| gene_product=TPR repeat/Tetratricopeptide

repeat, putative | transcript_product=TPR repeat/Tetratricopeptide

repeat, putative | location=LmjF.04:220364-224947(-)

| length=4584 | sequence_SO=chromosome | SO=protein_coding_gene

| is_pseudo=false

Length=4584

Score = 30.1 bits (32), Expect = 0.70

Identities = 24/28 (86%), Gaps = 1/28 (4%)

Strand=Plus/Minus

Query 42 TGCGCGGGTACGTGCGGTGTGTCGGCGA 69

|||||||||||||| ||| || |||||

Sbjct 4537 TGCGCGGGTACGTGGCGTG-GTGGGCGA 4511

Score = 27.4 bits (29), Expect = 8.5

Identities = 16/17 (94%), Gaps = 0/17 (0%)

Strand=Plus/Plus

Query 55 GCGGTGTGTCGGCGACA 71

|||||||||||| ||||

Sbjct 3852 GCGGTGTGTCGGTGACA 3868

>LmjF.03.0520:mRNA | gene=LmjF.03.0520 | organism=Leishmania_major_strain_Friedlin

| gene_product=hypothetical protein, conserved

| transcript_product=hypothetical protein, conserved

| location=LmjF.03:189457-192012(+) | length=2556 | sequence_SO=chromosome

| SO=protein_coding_gene | is_pseudo=false

Length=2556

Score = 30.1 bits (32), Expect = 0.70

Identities = 16/16 (100%), Gaps = 0/16 (0%)

Strand=Plus/Plus

Query 1 GGAGGAGGGGGAGGCG 16

||||||||||||||||

Sbjct 1035 GGAGGAGGGGGAGGCG 1050

>LmjF.31.0400:mRNA | gene=LmjF.31.0400 | organism=Leishmania_major_strain_Friedlin

| gene_product=calpain-like protein, putative

| transcript_product=calpain-like protein, putative |

location=LmjF.31:128306-132781(-) | length=4476 | sequence_SO=chromosome

| SO=protein_coding_gene | is_pseudo=false

Length=4476

Score = 29.2 bits (31), Expect = 2.4

Identities = 20/23 (87%), Gaps = 0/23 (0%)

Strand=Plus/Plus

Query 1 GGAGGAGGGGGAGGCGCTTGCGG 23

|||| |||||||||||| ||||

Sbjct 3753 GGAGTGGGGGGAGGCGCTGGCGG 3775

>LmjF.18.1350:mRNA | gene=LmjF.18.1350 | organism=Leishmania_major_strain_Friedlin

| gene_product=hypothetical protein, conserved

| transcript_product=hypothetical protein, conserved

| location=LmjF.18:586453-588351(+) | length=1899 | sequence_SO=chromosome

| SO=protein_coding_gene | is_pseudo=false

Length=1899

Score = 29.2 bits (31), Expect = 2.4

Identities = 20/23 (87%), Gaps = 0/23 (0%)

Strand=Plus/Minus

Query 42 TGCGCGGGTACGTGCGGTGTGTC 64

||||||||||||||| | | |||

Sbjct 1123 TGCGCGGGTACGTGCAGAGAGTC 1101

>LmjF.09.1210:mRNA | gene=LmjF.09.1210 | organism=Leishmania_major_strain_Friedlin

| gene_product=Zinc finger, C3HC4 type

(RING finger), putative | transcript_product=Zinc finger, C3HC4

type (RING finger), putative | location=LmjF.09:473005-474279(-)

| length=1275 | sequence_SO=chromosome | SO=protein_coding_gene

| is_pseudo=false

Length=1275

Score = 29.2 bits (31), Expect = 2.4

Identities = 20/23 (87%), Gaps = 0/23 (0%)

Strand=Plus/Minus

Query 1 GGAGGAGGGGGAGGCGCTTGCGG 23

|||| || ||||||||||||||

Sbjct 457 GGAGTAGCAGGAGGCGCTTGCGG 435

>LmjF.02.0410:mRNA | gene=LmjF.02.0410 | organism=Leishmania_major_strain_Friedlin

| gene_product=Kinetoplastid RNA editing

protein 1, putative | transcript_product=Kinetoplastid RNA

editing protein 1, putative | location=LmjF.02:180246-183311(-)

| length=3066 | sequence_SO=chromosome | SO=protein_coding_gene

| is_pseudo=false

Length=3066

Score = 29.2 bits (31), Expect = 2.4

Identities = 17/18 (94%), Gaps = 0/18 (0%)

Strand=Plus/Plus

Query 53 GTGCGGTGTGTCGGCGAC 70

||||| ||||||||||||

Sbjct 2878 GTGCGCTGTGTCGGCGAC 2895

>LmjF.36.1280:mRNA | gene=LmjF.36.1280 | organism=Leishmania_major_strain_Friedlin

| gene_product=tuzin-like protein | transcript_product=tuzin-like

protein | location=LmjF.36:471758-473536(+)

| length=1779 | sequence_SO=chromosome | SO=protein_coding_gene

| is_pseudo=false

Length=1779

Score = 28.3 bits (30), Expect = 2.4

Identities = 24/30 (80%), Gaps = 0/30 (0%)

Strand=Plus/Plus

Query 2 GAGGAGGGGGAGGCGCTTGCGGACATTGTG 31

||||| | ||||||||||| | || ||||

Sbjct 913 GAGGATGAGGAGGCGCTTGTGCGCAGTGTG 942

>LmjF.35.1700:mRNA | gene=LmjF.35.1700 | organism=Leishmania_major_strain_Friedlin

| gene_product=RNA re-capping enzyme, cytoplasmic,

putative | transcript_product=RNA re-capping enzyme,

cytoplasmic, putative | location=LmjF.35:720580-721668(-)

| length=1089 | sequence_SO=chromosome | SO=protein_coding_gene

| is_pseudo=false

Length=1089

Score = 28.3 bits (30), Expect = 2.4

Identities = 18/20 (90%), Gaps = 0/20 (0%)

Strand=Plus/Plus

Query 49 GTACGTGCGGTGTGTCGGCG 68

|| || ||||||||||||||

Sbjct 929 GTGCGAGCGGTGTGTCGGCG 948

>LmjF.34.1970:mRNA | gene=LmjF.34.1970 | organism=Leishmania_major_strain_Friedlin

| gene_product=tuzin-like protein | transcript_product=tuzin-like

protein | location=LmjF.34:862375-864369(-)

| length=1995 | sequence_SO=chromosome | SO=protein_coding_gene

| is_pseudo=false

Length=1995

Score = 28.3 bits (30), Expect = 2.4

Identities = 24/30 (80%), Gaps = 0/30 (0%)

Strand=Plus/Plus

Query 2 GAGGAGGGGGAGGCGCTTGCGGACATTGTG 31

||||| | ||||||||||| | || ||||

Sbjct 1129 GAGGATGAGGAGGCGCTTGTGCGCAGTGTG 1158

>LmjF.34.1950:mRNA | gene=LmjF.34.1950 | organism=Leishmania_major_strain_Friedlin

| gene_product=tuzin-like protein | transcript_product=tuzin-like

protein | location=LmjF.34:854843-856837(-)

| length=1995 | sequence_SO=chromosome | SO=protein_coding_gene

| is_pseudo=false

Length=1995

Score = 28.3 bits (30), Expect = 2.4

Identities = 24/30 (80%), Gaps = 0/30 (0%)

Strand=Plus/Plus

Query 2 GAGGAGGGGGAGGCGCTTGCGGACATTGTG 31

||||| | ||||||||||| | || ||||

Sbjct 1129 GAGGATGAGGAGGCGCTTGTGCGCAGTGTG 1158

>LmjF.34.1930:mRNA | gene=LmjF.34.1930 | organism=Leishmania_major_strain_Friedlin

| gene_product=tuzin-like protein | transcript_product=tuzin-like

protein | location=LmjF.34:847303-849297(-)

| length=1995 | sequence_SO=chromosome | SO=protein_coding_gene

| is_pseudo=false

Length=1995

Score = 28.3 bits (30), Expect = 2.4

Identities = 24/30 (80%), Gaps = 0/30 (0%)

Strand=Plus/Plus

Query 2 GAGGAGGGGGAGGCGCTTGCGGACATTGTG 31

||||| | ||||||||||| | || ||||

Sbjct 1129 GAGGATGAGGAGGCGCTTGTGCGCAGTGTG 1158

>LmjF.34.1910:mRNA | gene=LmjF.34.1910 | organism=Leishmania_major_strain_Friedlin

| gene_product=tuzin-like protein | transcript_product=tuzin-like

protein | location=LmjF.34:839756-841750(-)

| length=1995 | sequence_SO=chromosome | SO=protein_coding_gene

| is_pseudo=false

Length=1995

Score = 28.3 bits (30), Expect = 2.4

Identities = 24/30 (80%), Gaps = 0/30 (0%)

Strand=Plus/Plus

Query 2 GAGGAGGGGGAGGCGCTTGCGGACATTGTG 31

||||| | ||||||||||| | || ||||

Sbjct 1129 GAGGATGAGGAGGCGCTTGTGCGCAGTGTG 1158

>LmjF.34.1890:mRNA | gene=LmjF.34.1890 | organism=Leishmania_major_strain_Friedlin

| gene_product=tuzin-like protein | transcript_product=tuzin-like

protein | location=LmjF.34:832221-834215(-)

| length=1995 | sequence_SO=chromosome | SO=protein_coding_gene

| is_pseudo=false

Length=1995

Score = 28.3 bits (30), Expect = 2.4

Identities = 24/30 (80%), Gaps = 0/30 (0%)

Strand=Plus/Plus

Query 2 GAGGAGGGGGAGGCGCTTGCGGACATTGTG 31

||||| | ||||||||||| | || ||||

Sbjct 1129 GAGGATGAGGAGGCGCTTGTGCGCAGTGTG 1158

>LmjF.34.1870:mRNA | gene=LmjF.34.1870 | organism=Leishmania_major_strain_Friedlin

| gene_product=tuzin-like protein | transcript_product=tuzin-like

protein | location=LmjF.34:824673-826667(-)

| length=1995 | sequence_SO=chromosome | SO=protein_coding_gene

| is_pseudo=false

Length=1995

Score = 28.3 bits (30), Expect = 2.4

Identities = 24/30 (80%), Gaps = 0/30 (0%)

Strand=Plus/Plus

Query 2 GAGGAGGGGGAGGCGCTTGCGGACATTGTG 31

||||| | ||||||||||| | || ||||

Sbjct 1129 GAGGATGAGGAGGCGCTTGTGCGCAGTGTG 1158

>LmjF.34.1850:mRNA | gene=LmjF.34.1850 | organism=Leishmania_major_strain_Friedlin

| gene_product=tuzin-like protein | transcript_product=tuzin-like

protein | location=LmjF.34:817133-819127(-)

| length=1995 | sequence_SO=chromosome | SO=protein_coding_gene

| is_pseudo=false

Length=1995

Score = 28.3 bits (30), Expect = 2.4

Identities = 24/30 (80%), Gaps = 0/30 (0%)

Strand=Plus/Plus

Query 2 GAGGAGGGGGAGGCGCTTGCGGACATTGTG 31

||||| | ||||||||||| | || ||||

Sbjct 1129 GAGGATGAGGAGGCGCTTGTGCGCAGTGTG 1158

>LmjF.34.1830:mRNA | gene=LmjF.34.1830 | organism=Leishmania_major_strain_Friedlin

| gene_product=tuzin-like protein | transcript_product=tuzin-like

protein | location=LmjF.34:809850-811844(-)

| length=1995 | sequence_SO=chromosome | SO=protein_coding_gene

| is_pseudo=false

Length=1995

Score = 28.3 bits (30), Expect = 2.4

Identities = 24/30 (80%), Gaps = 0/30 (0%)

Strand=Plus/Plus

Query 2 GAGGAGGGGGAGGCGCTTGCGGACATTGTG 31

||||| | ||||||||||| | || ||||

Sbjct 1129 GAGGATGAGGAGGCGCTTGTGCGCAGTGTG 1158

>LmjF.34.1810:mRNA | gene=LmjF.34.1810 | organism=Leishmania_major_strain_Friedlin

| gene_product=tuzin-like protein | transcript_product=tuzin-like

protein | location=LmjF.34:802311-804305(-)

| length=1995 | sequence_SO=chromosome | SO=protein_coding_gene

| is_pseudo=false

Length=1995

Score = 28.3 bits (30), Expect = 2.4

Identities = 24/30 (80%), Gaps = 0/30 (0%)

Strand=Plus/Plus

Query 2 GAGGAGGGGGAGGCGCTTGCGGACATTGTG 31

||||| | ||||||||||| | || ||||

Sbjct 1129 GAGGATGAGGAGGCGCTTGTGCGCAGTGTG 1158

>LmjF.34.1790:mRNA | gene=LmjF.34.1790 | organism=Leishmania_major_strain_Friedlin

| gene_product=tuzin-like protein | transcript_product=tuzin-like

protein | location=LmjF.34:794769-796763(-)

| length=1995 | sequence_SO=chromosome | SO=protein_coding_gene

| is_pseudo=false

Length=1995

Score = 28.3 bits (30), Expect = 2.4

Identities = 24/30 (80%), Gaps = 0/30 (0%)

Strand=Plus/Plus

Query 2 GAGGAGGGGGAGGCGCTTGCGGACATTGTG 31

||||| | ||||||||||| | || ||||

Sbjct 1129 GAGGATGAGGAGGCGCTTGTGCGCAGTGTG 1158

>LmjF.34.1770:mRNA | gene=LmjF.34.1770 | organism=Leishmania_major_strain_Friedlin

| gene_product=tuzin-like protein | transcript_product=tuzin-like

protein | location=LmjF.34:787216-789210(-)

| length=1995 | sequence_SO=chromosome | SO=protein_coding_gene

| is_pseudo=false

Length=1995

Score = 28.3 bits (30), Expect = 2.4

Identities = 24/30 (80%), Gaps = 0/30 (0%)

Strand=Plus/Plus

Query 2 GAGGAGGGGGAGGCGCTTGCGGACATTGTG 31

||||| | ||||||||||| | || ||||

Sbjct 1129 GAGGATGAGGAGGCGCTTGTGCGCAGTGTG 1158

>LmjF.34.1750:mRNA | gene=LmjF.34.1750 | organism=Leishmania_major_strain_Friedlin

| gene_product=tuzin-like protein | transcript_product=tuzin-like

protein | location=LmjF.34:779677-781671(-)

| length=1995 | sequence_SO=chromosome | SO=protein_coding_gene

| is_pseudo=false

Length=1995

Score = 28.3 bits (30), Expect = 2.4

Identities = 24/30 (80%), Gaps = 0/30 (0%)

Strand=Plus/Plus

Query 2 GAGGAGGGGGAGGCGCTTGCGGACATTGTG 31

||||| | ||||||||||| | || ||||

Sbjct 1129 GAGGATGAGGAGGCGCTTGTGCGCAGTGTG 1158

>LmjF.34.1730:mRNA | gene=LmjF.34.1730 | organism=Leishmania_major_strain_Friedlin

| gene_product=tuzin-like protein | transcript_product=tuzin-like

protein | location=LmjF.34:772144-774138(-)

| length=1995 | sequence_SO=chromosome | SO=protein_coding_gene

| is_pseudo=false

Length=1995

Score = 28.3 bits (30), Expect = 2.4

Identities = 24/30 (80%), Gaps = 0/30 (0%)

Strand=Plus/Plus

Query 2 GAGGAGGGGGAGGCGCTTGCGGACATTGTG 31

||||| | ||||||||||| | || ||||

Sbjct 1129 GAGGATGAGGAGGCGCTTGTGCGCAGTGTG 1158

>LmjF.34.1710:mRNA | gene=LmjF.34.1710 | organism=Leishmania_major_strain_Friedlin

| gene_product=tuzin-like protein | transcript_product=tuzin-like

protein | location=LmjF.34:764602-766596(-)

| length=1995 | sequence_SO=chromosome | SO=protein_coding_gene

| is_pseudo=false

Length=1995

Score = 28.3 bits (30), Expect = 2.4

Identities = 24/30 (80%), Gaps = 0/30 (0%)

Strand=Plus/Plus

Query 2 GAGGAGGGGGAGGCGCTTGCGGACATTGTG 31

||||| | ||||||||||| | || ||||

Sbjct 1129 GAGGATGAGGAGGCGCTTGTGCGCAGTGTG 1158

>LmjF.34.1690:mRNA | gene=LmjF.34.1690 | organism=Leishmania_major_strain_Friedlin

| gene_product=tuzin-like protein | transcript_product=tuzin-like

protein | location=LmjF.34:757053-759047(-)

| length=1995 | sequence_SO=chromosome | SO=protein_coding_gene

| is_pseudo=false

Length=1995

Score = 28.3 bits (30), Expect = 2.4

Identities = 24/30 (80%), Gaps = 0/30 (0%)

Strand=Plus/Plus

Query 2 GAGGAGGGGGAGGCGCTTGCGGACATTGTG 31

||||| | ||||||||||| | || ||||

Sbjct 1129 GAGGATGAGGAGGCGCTTGTGCGCAGTGTG 1158

>LmjF.34.1670:mRNA | gene=LmjF.34.1670 | organism=Leishmania_major_strain_Friedlin

| gene_product=tuzin-like protein | transcript_product=tuzin-like

protein | location=LmjF.34:749524-751518(-)

| length=1995 | sequence_SO=chromosome | SO=protein_coding_gene

| is_pseudo=false

Length=1995

Score = 28.3 bits (30), Expect = 2.4

Identities = 24/30 (80%), Gaps = 0/30 (0%)

Strand=Plus/Plus

Query 2 GAGGAGGGGGAGGCGCTTGCGGACATTGTG 31

||||| | ||||||||||| | || ||||

Sbjct 1129 GAGGATGAGGAGGCGCTTGTGCGCAGTGTG 1158

>LmjF.34.1650:mRNA | gene=LmjF.34.1650 | organism=Leishmania_major_strain_Friedlin

| gene_product=tuzin-like protein | transcript_product=tuzin-like

protein | location=LmjF.34:741995-743989(-)

| length=1995 | sequence_SO=chromosome | SO=protein_coding_gene

| is_pseudo=false

Length=1995

Score = 28.3 bits (30), Expect = 2.4

Identities = 24/30 (80%), Gaps = 0/30 (0%)

Strand=Plus/Plus

Query 2 GAGGAGGGGGAGGCGCTTGCGGACATTGTG 31

||||| | ||||||||||| | || ||||

Sbjct 1129 GAGGATGAGGAGGCGCTTGTGCGCAGTGTG 1158

>LmjF.34.1630:mRNA | gene=LmjF.34.1630 | organism=Leishmania_major_strain_Friedlin

| gene_product=tuzin-like protein | transcript_product=tuzin-like

protein | location=LmjF.34:734394-736388(-)

| length=1995 | sequence_SO=chromosome | SO=protein_coding_gene

| is_pseudo=false

Length=1995

Score = 28.3 bits (30), Expect = 2.4

Identities = 24/30 (80%), Gaps = 0/30 (0%)

Strand=Plus/Plus

Query 2 GAGGAGGGGGAGGCGCTTGCGGACATTGTG 31

||||| | ||||||||||| | || ||||

Sbjct 1129 GAGGATGAGGAGGCGCTTGTGCGCAGTGTG 1158

>LmjF.34.1610:mRNA | gene=LmjF.34.1610 | organism=Leishmania_major_strain_Friedlin

| gene_product=tuzin-like protein | transcript_product=tuzin-like

protein | location=LmjF.34:726853-728847(-)

| length=1995 | sequence_SO=chromosome | SO=protein_coding_gene

| is_pseudo=false

Length=1995

Score = 28.3 bits (30), Expect = 2.4

Identities = 24/30 (80%), Gaps = 0/30 (0%)

Strand=Plus/Plus

Query 2 GAGGAGGGGGAGGCGCTTGCGGACATTGTG 31

||||| | ||||||||||| | || ||||

Sbjct 1129 GAGGATGAGGAGGCGCTTGTGCGCAGTGTG 1158

>LmjF.34.1590:mRNA | gene=LmjF.34.1590 | organism=Leishmania_major_strain_Friedlin

| gene_product=tuzin-like protein | transcript_product=tuzin-like

protein | location=LmjF.34:719588-721582(-)

| length=1995 | sequence_SO=chromosome | SO=protein_coding_gene

| is_pseudo=false

Length=1995

Score = 28.3 bits (30), Expect = 2.4

Identities = 24/30 (80%), Gaps = 0/30 (0%)

Strand=Plus/Plus

Query 2 GAGGAGGGGGAGGCGCTTGCGGACATTGTG 31

||||| | ||||||||||| | || ||||

Sbjct 1129 GAGGATGAGGAGGCGCTTGTGCGCAGTGTG 1158

>LmjF.34.1570:mRNA | gene=LmjF.34.1570 | organism=Leishmania_major_strain_Friedlin

| gene_product=tuzin-like protein | transcript_product=tuzin-like

protein | location=LmjF.34:712034-714028(-)

| length=1995 | sequence_SO=chromosome | SO=protein_coding_gene

| is_pseudo=false

Length=1995

Score = 28.3 bits (30), Expect = 2.4

Identities = 24/30 (80%), Gaps = 0/30 (0%)

Strand=Plus/Plus

Query 2 GAGGAGGGGGAGGCGCTTGCGGACATTGTG 31

||||| | ||||||||||| | || ||||

Sbjct 1129 GAGGATGAGGAGGCGCTTGTGCGCAGTGTG 1158

>LmjF.34.0550:mRNA | gene=LmjF.34.0550 | organism=Leishmania_major_strain_Friedlin

| gene_product=Cysteine leucine rich protein

| transcript_product=Cysteine leucine rich protein | location=LmjF.34:225827-227698(+)

| length=1872 | sequence_SO=chromosome

| SO=protein_coding_gene | is_pseudo=false

Length=1872

Score = 28.3 bits (30), Expect = 2.4

Identities = 15/15 (100%), Gaps = 0/15 (0%)

Strand=Plus/Minus

Query 1 GGAGGAGGGGGAGGC 15

|||||||||||||||

Sbjct 645 GGAGGAGGGGGAGGC 631

>LmjF.33.1730:mRNA | gene=LmjF.33.1730 | organism=Leishmania_major_strain_Friedlin

| gene_product=mitochondrial RNA binding

complex 1 subunit, putative | transcript_product=mitochondrial

RNA binding complex 1 subunit, putative | location=LmjF.33:801619-804300(+)

| length=2682 | sequence_SO=chromosome

| SO=protein_coding_gene | is_pseudo=false

Length=2682

Score = 28.3 bits (30), Expect = 2.4

Identities = 21/25 (84%), Gaps = 0/25 (0%)

Strand=Plus/Plus

Query 22 GGACATTGTGGCGATCATGGTGCGC 46

||||||||||| | |||||||||

Sbjct 1887 GGACATTGTGGAGGAGATGGTGCGC 1911

>LmjF.31.0860:mRNA | gene=LmjF.31.0860 | organism=Leishmania_major_strain_Friedlin

| gene_product=ras-like small GTPases,

putative | transcript_product=ras-like small GTPases, putative

| location=LmjF.31:315900-317732(-) | length=1833 | sequence_SO=chromosome

| SO=protein_coding_gene | is_pseudo=false

Length=1833

Score = 28.3 bits (30), Expect = 2.4

Identities = 15/15 (100%), Gaps = 0/15 (0%)

Strand=Plus/Minus

Query 59 TGTGTCGGCGACAAG 73

|||||||||||||||

Sbjct 1145 TGTGTCGGCGACAAG 1131

>LmjF.31.0810:mRNA | gene=LmjF.31.0810 | organism=Leishmania_major_strain_Friedlin

| gene_product=hypothetical protein, conserved

| transcript_product=hypothetical protein, conserved

| location=LmjF.31:292899-294788(-) | length=1890 | sequence_SO=chromosome

| SO=protein_coding_gene | is_pseudo=false

Length=1890

Score = 28.3 bits (30), Expect = 2.4

Identities = 18/20 (90%), Gaps = 0/20 (0%)

Strand=Plus/Plus

Query 28 TGTGGCGATCATGGTGCGCG 47

||||||||||||| ||| ||

Sbjct 1437 TGTGGCGATCATGTTGCACG 1456

>LmjF.28.0990:mRNA | gene=LmjF.28.0990 | organism=Leishmania_major_strain_Friedlin

| gene_product=Serine incorporator (Serinc),

putative | transcript_product=Serine incorporator (Serinc),

putative | location=LmjF.28:359922-361172(+) | length=1251

| sequence_SO=chromosome | SO=protein_coding_gene | is_pseudo=false

Length=1251

Score = 28.3 bits (30), Expect = 2.4

Identities = 18/20 (90%), Gaps = 0/20 (0%)

Strand=Plus/Plus

Query 30 TGGCGATCATGGTGCGCGGG 49

|| |||| ||||||||||||

Sbjct 128 TGTCGATGATGGTGCGCGGG 147

>LmjF.27.0500:mRNA | gene=LmjF.27.0500 | organism=Leishmania_major_strain_Friedlin

| gene_product=calpain-like cysteine peptidase,

putative | transcript_product=calpain-like cysteine

peptidase, putative | location=LmjF.27:164885-183379(+) | length=18495

| sequence_SO=chromosome | SO=protein_coding_gene

| is_pseudo=false

Length=18495

Score = 28.3 bits (30), Expect = 2.4

Identities = 18/20 (90%), Gaps = 0/20 (0%)

Strand=Plus/Plus

Query 4 GGAGGGGGAGGCGCTTGCGG 23

|| || ||||||||||||||

Sbjct 12297 GGCGGAGGAGGCGCTTGCGG 12316

>LmjF.26.1330:mRNA | gene=LmjF.26.1330 | organism=Leishmania_major_strain_Friedlin

| gene_product=hypothetical protein, conserved

| transcript_product=hypothetical protein, conserved

| location=LmjF.26:453251-454486(+) | length=1236 | sequence_SO=chromosome

| SO=protein_coding_gene | is_pseudo=false

Length=1236

Score = 28.3 bits (30), Expect = 2.4

Identities = 21/25 (84%), Gaps = 0/25 (0%)

Strand=Plus/Plus

Query 9 GGGAGGCGCTTGCGGACATTGTGGC 33

|||| || ||||||||||| ||||

Sbjct 908 GGGATGCCCTTGCGGACATGCTGGC 932

>LmjF.25.1570:mRNA | gene=LmjF.25.1570 | organism=Leishmania_major_strain_Friedlin

| gene_product=ER-golgi trafficking TRAPP

I complex 85 kDa subunit, putative | transcript_product=ER-golgi

trafficking TRAPP I complex 85 kDa subunit, putative

| location=LmjF.25:635613-642407(-) | length=6795 | sequence_SO=chromosome

| SO=protein_coding_gene | is_pseudo=false

Length=6795

Score = 28.3 bits (30), Expect = 2.4

Identities = 15/15 (100%), Gaps = 0/15 (0%)

Strand=Plus/Minus

Query 1 GGAGGAGGGGGAGGC 15

|||||||||||||||

Sbjct 1017 GGAGGAGGGGGAGGC 1003

>LmjF.25.0680:mRNA | gene=LmjF.25.0680 | organism=Leishmania_major_strain_Friedlin

| gene_product=myosin heavy chain kinase

c-like protein | transcript_product=myosin heavy chain kinase

c-like protein | location=LmjF.25:241220-246400(-) | length=5181

| sequence_SO=chromosome | SO=protein_coding_gene |

is_pseudo=false

Length=5181

Score = 28.3 bits (30), Expect = 2.4

Identities = 15/15 (100%), Gaps = 0/15 (0%)

Strand=Plus/Plus

Query 29 GTGGCGATCATGGTG 43

|||||||||||||||

Sbjct 1901 GTGGCGATCATGGTG 1915

>LmjF.24.2090:mRNA | gene=LmjF.24.2090 | organism=Leishmania_major_strain_Friedlin

| gene_product=Kelch motif/Galactose oxidase,

central domain containing protein, putative | transcript_product=Kelch

motif/Galactose oxidase, central domain containing

protein, putative | location=LmjF.24:766741-767877(-)

| length=1137 | sequence_SO=chromosome | SO=protein_coding_gene

| is_pseudo=false

Length=1137

Score = 28.3 bits (30), Expect = 2.4

Identities = 18/20 (90%), Gaps = 0/20 (0%)

Strand=Plus/Minus

Query 35 ATCATGGTGCGCGGGTACGT 54

|||||||||||| ||||||

Sbjct 593 ATCATGGTGCGCTCGTACGT 574

>LmjF.14.1450:mRNA | gene=LmjF.14.1450 | organism=Leishmania_major_strain_Friedlin

| gene_product=Vacuolar 14 Fab1-binding

region/Vacuolar protein 14 C-terminal Fig4p binding, putative

| transcript_product=Vacuolar 14 Fab1-binding region/Vacuolar

protein 14 C-terminal Fig4p binding, putative | location=LmjF.14:608047-610104(+)

| length=2058 | sequence_SO=chromosome

| SO=protein_coding_gene | is_pseudo=false

Length=2058

Score = 28.3 bits (30), Expect = 2.4

Identities = 15/15 (100%), Gaps = 0/15 (0%)

Strand=Plus/Plus

Query 49 GTACGTGCGGTGTGT 63

|||||||||||||||

Sbjct 226 GTACGTGCGGTGTGT 240

>LmjF.14.0340:mRNA | gene=LmjF.14.0340 | organism=Leishmania_major_strain_Friedlin

| gene_product=inositol polyphosphate kinase-like

protein, putative | transcript_product=inositol polyphosphate

kinase-like protein, putative | location=LmjF.14:99176-102436(+)

| length=3261 | sequence_SO=chromosome | SO=protein_coding_gene

| is_pseudo=false

Length=3261

Score = 28.3 bits (30), Expect = 2.4

Identities = 15/15 (100%), Gaps = 0/15 (0%)

Strand=Plus/Minus

Query 2 GAGGAGGGGGAGGCG 16

|||||||||||||||

Sbjct 1772 GAGGAGGGGGAGGCG 1758

>LmjF.13.0240.2:mRNA | gene=LmjF.13.0240 | organism=Leishmania_major_strain_Friedlin

| gene_product=Domain of unknown function

(DUF4200), putative | transcript_product=Domain of unknown

function (DUF4200), putative | location=LmjF.13:76031-76954(-)

| length=1013 | sequence_SO=chromosome | SO=protein_coding_gene

| is_pseudo=false

Length=1013

Score = 28.3 bits (30), Expect = 2.4

Identities = 15/15 (100%), Gaps = 0/15 (0%)

Strand=Plus/Plus

Query 3 AGGAGGGGGAGGCGC 17

|||||||||||||||

Sbjct 208 AGGAGGGGGAGGCGC 222

>LmjF.13.0240.1:mRNA | gene=LmjF.13.0240 | organism=Leishmania_major_strain_Friedlin

| gene_product=Domain of unknown function

(DUF4200), putative | transcript_product=Domain of unknown

function (DUF4200), putative | location=LmjF.13:76031-77233(-)

| length=2748 | sequence_SO=chromosome | SO=protein_coding_gene

| is_pseudo=false

Length=2748

Score = 28.3 bits (30), Expect = 2.4

Identities = 15/15 (100%), Gaps = 0/15 (0%)

Strand=Plus/Plus

Query 3 AGGAGGGGGAGGCGC 17

|||||||||||||||

Sbjct 1943 AGGAGGGGGAGGCGC 1957

>LmjF.12.0650:mRNA | gene=LmjF.12.0650 | organism=Leishmania_major_strain_Friedlin

| gene_product=hypothetical protein, conserved

| transcript_product=hypothetical protein, conserved

| location=LmjF.12:351234-353690(+) | length=2457 | sequence_SO=chromosome

| SO=protein_coding_gene | is_pseudo=false

Length=2457

Score = 28.3 bits (30), Expect = 2.4

Identities = 15/15 (100%), Gaps = 0/15 (0%)

Strand=Plus/Minus

Query 56 CGGTGTGTCGGCGAC 70

|||||||||||||||

Sbjct 117 CGGTGTGTCGGCGAC 103

>LmjF.09.0990:mRNA | gene=LmjF.09.0990 | organism=Leishmania_major_strain_Friedlin

| gene_product=N-terminal region of Chorein,

a TM vesicle-mediated sorter, putative | transcript_product=N-terminal

region of Chorein, a TM vesicle-mediated sorter,

putative | location=LmjF.09:387471-403616(-) | length=16146

| sequence_SO=chromosome | SO=protein_coding_gene | is_pseudo=false

Length=16146

Score = 28.3 bits (30), Expect = 2.4

Identities = 18/20 (90%), Gaps = 0/20 (0%)

Strand=Plus/Plus

Query 29 GTGGCGATCATGGTGCGCGG 48

|||||||||||| || ||||

Sbjct 11726 GTGGCGATCATGATGAGCGG 11745

>LmjF.36.5750:mRNA | gene=LmjF.36.5750 | organism=Leishmania_major_strain_Friedlin

| gene_product=pseudouridine synthase TruD,

putative | transcript_product=pseudouridine synthase TruD,

putative | location=LmjF.36:2202760-2204802(-) | length=2043

| sequence_SO=chromosome | SO=protein_coding_gene | is_pseudo=false

Length=2043

Score = 27.4 bits (29), Expect = 8.5

Identities = 16/17 (94%), Gaps = 0/17 (0%)

Strand=Plus/Minus

Query 52 CGTGCGGTGTGTCGGCG 68

||||||||||| |||||

Sbjct 59 CGTGCGGTGTGCCGGCG 43

>LmjF.36.3960:mRNA | gene=LmjF.36.3960 | organism=Leishmania_major_strain_Friedlin

| gene_product=hypothetical protein, unknown

function | transcript_product=hypothetical protein, unknown

function | location=LmjF.36:1510736-1513789(+) | length=3054

| sequence_SO=chromosome | SO=protein_coding_gene | is_pseudo=false

Length=3054

Score = 27.4 bits (29), Expect = 8.5

Identities = 16/17 (94%), Gaps = 0/17 (0%)

Strand=Plus/Plus

Query 54 TGCGGTGTGTCGGCGAC 70

||| |||||||||||||

Sbjct 1960 TGCCGTGTGTCGGCGAC 1976

>LmjF.36.1370:mRNA | gene=LmjF.36.1370 | organism=Leishmania_major_strain_Friedlin

| gene_product=Valosin-containing protein,

putative | transcript_product=Valosin-containing protein,

putative | location=LmjF.36:498032-500386(-) | length=2355

| sequence_SO=chromosome | SO=protein_coding_gene | is_pseudo=false

Length=2355

Score = 27.4 bits (29), Expect = 8.5

Identities = 21/24 (88%), Gaps = 1/24 (4%)

Strand=Plus/Plus

Query 2 GAGGAGGGGGAGGCGCTTG-CGGA 24

||||| | ||||||||||| ||||

Sbjct 547 GAGGACGAGGAGGCGCTTGACGGA 570

>LmjF.34.4620:mRNA | gene=LmjF.34.4620 | organism=Leishmania_major_strain_Friedlin

| gene_product=cyclic nucleotide-binding

domain containing protein, putative | transcript_product=cyclic

nucleotide-binding domain containing protein, putative

| location=LmjF.34:1862057-1864462(+) | length=2406 | sequence_SO=chromosome

| SO=protein_coding_gene | is_pseudo=false

Length=2406

Score = 27.4 bits (29), Expect = 8.5

Identities = 16/17 (94%), Gaps = 0/17 (0%)

Strand=Plus/Plus

Query 36 TCATGGTGCGCGGGTAC 52

|||||||||||| ||||

Sbjct 293 TCATGGTGCGCGAGTAC 309

>LmjF.19.1150:mRNA | gene=LmjF.19.1150 | organism=Leishmania_major_strain_Friedlin

| gene_product=hypothetical protein, conserved

| transcript_product=hypothetical protein, conserved

| location=LmjF.19:497252-505222(+) | length=7971 | sequence_SO=chromosome

| SO=protein_coding_gene | is_pseudo=false

Length=7971

Score = 27.4 bits (29), Expect = 8.5

Identities = 16/17 (94%), Gaps = 0/17 (0%)

Strand=Plus/Minus

Query 1 GGAGGAGGGGGAGGCGC 17

||||||||||| |||||

Sbjct 114 GGAGGAGGGGGTGGCGC 98

>LmjF.18.1140:mRNA | gene=LmjF.18.1140 | organism=Leishmania_major_strain_Friedlin

| gene_product=hypothetical protein, conserved

| transcript_product=hypothetical protein, conserved

| location=LmjF.18:479419-482898(+) | length=3480 | sequence_SO=chromosome

| SO=protein_coding_gene | is_pseudo=false

Length=3480

Score = 27.4 bits (29), Expect = 8.5

Identities = 16/17 (94%), Gaps = 0/17 (0%)

Strand=Plus/Minus

Query 2 GAGGAGGGGGAGGCGCT 18

|||| ||||||||||||

Sbjct 689 GAGGTGGGGGAGGCGCT 673

>LmjF.14.1110:mRNA | gene=LmjF.14.1110 | organism=Leishmania_major_strain_Friedlin

| gene_product=kinesin K39, putative |

transcript_product=kinesin K39, putative | location=LmjF.14:470573-474472(+)

| length=3900 | sequence_SO=chromosome | SO=protein_coding_gene

| is_pseudo=false

Length=3900

Score = 27.4 bits (29), Expect = 8.5

Identities = 16/17 (94%), Gaps = 0/17 (0%)

Strand=Plus/Plus

Query 10 GGAGGCGCTTGCGGACA 26

||||||||||| |||||

Sbjct 1830 GGAGGCGCTTGAGGACA 1846

>LmjF.12.1120:mRNA | gene=LmjF.12.1120 | organism=Leishmania_major_strain_Friedlin

| gene_product=Flagellum attachment zone

protein 2 | transcript_product=Flagellum attachment zone protein

2 | location=LmjF.12:565041-568925(+) | length=3885 |

sequence_SO=chromosome | SO=protein_coding_gene | is_pseudo=false

Length=3885

Score = 27.4 bits (29), Expect = 8.5

Identities = 16/17 (94%), Gaps = 0/17 (0%)

Strand=Plus/Plus

Query 5 GAGGGGGAGGCGCTTGC 21

||||||||||| |||||

Sbjct 2842 GAGGGGGAGGCTCTTGC 2858

>LmjF.09.0760:mRNA | gene=LmjF.09.0760 | organism=Leishmania_major_strain_Friedlin

| gene_product=hypothetical protein, conserved

| transcript_product=hypothetical protein, conserved

| location=LmjF.09:300595-317040(-) | length=16446 | sequence_SO=chromosome

| SO=protein_coding_gene | is_pseudo=false

Length=16446

Score = 27.4 bits (29), Expect = 8.5

Identities = 16/17 (94%), Gaps = 0/17 (0%)

Strand=Plus/Plus

Query 15 CGCTTGCGGACATTGTG 31

||||||||||| |||||

Sbjct 7229 CGCTTGCGGACTTTGTG 7245

>LmjF.06.0320:mRNA | gene=LmjF.06.0320 | organism=Leishmania_major_strain_Friedlin

| gene_product=hypothetical protein, conserved

| transcript_product=hypothetical protein, conserved

| location=LmjF.06:106808-109882(-) | length=3075 | sequence_SO=chromosome

| SO=protein_coding_gene | is_pseudo=false

Length=3075

Score = 27.4 bits (29), Expect = 8.5

Identities = 16/17 (94%), Gaps = 0/17 (0%)

Strand=Plus/Plus

Query 33 CGATCATGGTGCGCGGG 49

|| ||||||||||||||

Sbjct 740 CGGTCATGGTGCGCGGG 756

>LmjF.03.0360:mRNA | gene=LmjF.03.0360 | organism=Leishmania_major_strain_Friedlin

| gene_product=hypothetical protein | transcript_product=hypothetical

protein | location=LmjF.03:108057-113039(+)

| length=4983 | sequence_SO=chromosome | SO=protein_coding_gene

| is_pseudo=false

Length=4983

Score = 27.4 bits (29), Expect = 8.5

Identities = 22/27 (81%), Gaps = 0/27 (0%)

Strand=Plus/Minus

Query 1 GGAGGAGGGGGAGGCGCTTGCGGACAT 27

|| || || || || ||||||||||||

Sbjct 1325 GGTGGGGGTGGCGGTGCTTGCGGACAT 1299

>LmjF.36.6430:mRNA | gene=LmjF.36.6430 | organism=Leishmania_major_strain_Friedlin

| gene_product=protein transport protein

Sec23-like protein | transcript_product=protein transport

protein Sec23-like protein | location=LmjF.36:2490349-2492901(-)

| length=2553 | sequence_SO=chromosome | SO=protein_coding_gene

| is_pseudo=false

Length=2553

Score = 26.5 bits (28), Expect = 8.5

Identities = 17/19 (89%), Gaps = 0/19 (0%)

Strand=Plus/Minus

Query 37 CATGGTGCGCGGGTACGTG 55

||||| ||||||||||||

Sbjct 873 CATGGCACGCGGGTACGTG 855

>LmjF.36.5530:mRNA | gene=LmjF.36.5530 | organism=Leishmania_major_strain_Friedlin

| gene_product=Phosphorylated CTD interacting

factor 1 WW domain containing protein, putative | transcript_product=Phosphorylated

CTD interacting factor 1 WW domain

containing protein, putative | location=LmjF.36:2141029-2142999(-)

| length=1971 | sequence_SO=chromosome | SO=protein_coding_gene

| is_pseudo=false

Length=1971

Score = 26.5 bits (28), Expect = 8.5

Identities = 14/14 (100%), Gaps = 0/14 (0%)

Strand=Plus/Plus

Query 55 GCGGTGTGTCGGCG 68

||||||||||||||

Sbjct 791 GCGGTGTGTCGGCG 804

>LmjF.36.5380:mRNA | gene=LmjF.36.5380 | organism=Leishmania_major_strain_Friedlin

| gene_product=NADH dehydrogenase, putative

| transcript_product=NADH dehydrogenase, putative | location=LmjF.36:2086903-2088486(-)

| length=1584 | sequence_SO=chromosome

| SO=protein_coding_gene | is_pseudo=false

Length=1584

Score = 26.5 bits (28), Expect = 8.5

Identities = 14/14 (100%), Gaps = 0/14 (0%)

Strand=Plus/Minus

Query 11 GAGGCGCTTGCGGA 24

||||||||||||||

Sbjct 480 GAGGCGCTTGCGGA 467

>LmjF.36.4520:mRNA | gene=LmjF.36.4520 | organism=Leishmania_major_strain_Friedlin

| gene_product=hypothetical protein, conserved

| transcript_product=hypothetical protein, conserved

| location=LmjF.36:1742192-1745356(+) | length=3165 | sequence_SO=chromosome

| SO=protein_coding_gene | is_pseudo=false

Length=3165

Score = 26.5 bits (28), Expect = 8.5

Identities = 14/14 (100%), Gaps = 0/14 (0%)

Strand=Plus/Plus

Query 52 CGTGCGGTGTGTCG 65

||||||||||||||

Sbjct 101 CGTGCGGTGTGTCG 114

>LmjF.36.0850:mRNA | gene=LmjF.36.0850 | organism=Leishmania_major_strain_Friedlin

| gene_product=hypothetical protein, conserved

| transcript_product=hypothetical protein, conserved

| location=LmjF.36:310531-313197(+) | length=2667 | sequence_SO=chromosome

| SO=protein_coding_gene | is_pseudo=false

Length=2667

Score = 26.5 bits (28), Expect = 8.5

Identities = 17/19 (89%), Gaps = 0/19 (0%)

Strand=Plus/Minus

Query 28 TGTGGCGATCATGGTGCGC 46

||||||||||| |||| ||

Sbjct 1410 TGTGGCGATCAAGGTGTGC 1392

>LmjF.34.3200:mRNA | gene=LmjF.34.3200 | organism=Leishmania_major_strain_Friedlin

| gene_product=hypothetical protein, conserved

| transcript_product=hypothetical protein, conserved

| location=LmjF.34:1421100-1424309(+) | length=3210 | sequence_SO=chromosome

| SO=protein_coding_gene | is_pseudo=false

Length=3210

Score = 26.5 bits (28), Expect = 8.5

Identities = 14/14 (100%), Gaps = 0/14 (0%)

Strand=Plus/Plus

Query 1 GGAGGAGGGGGAGG 14

||||||||||||||

Sbjct 2625 GGAGGAGGGGGAGG 2638

>LmjF.34.0660:mRNA | gene=LmjF.34.0660 | organism=Leishmania_major_strain_Friedlin

| gene_product=hypothetical protein, unknown

function | transcript_product=hypothetical protein, unknown

function | location=LmjF.34:286195-289878(+) | length=3684

| sequence_SO=chromosome | SO=protein_coding_gene | is_pseudo=false

Length=3684

Score = 26.5 bits (28), Expect = 8.5

Identities = 14/14 (100%), Gaps = 0/14 (0%)

Strand=Plus/Minus

Query 1 GGAGGAGGGGGAGG 14

||||||||||||||

Sbjct 128 GGAGGAGGGGGAGG 115

>LmjF.31.0190:mRNA | gene=LmjF.31.0190 | organism=Leishmania_major_strain_Friedlin

| gene_product=nucleolar protein, putative

| transcript_product=nucleolar protein, putative | location=LmjF.31:48774-50663(-)

| length=1890 | sequence_SO=chromosome

| SO=protein_coding_gene | is_pseudo=false

Length=1890

Score = 26.5 bits (28), Expect = 8.5

Identities = 14/14 (100%), Gaps = 0/14 (0%)

Strand=Plus/Plus

Query 1 GGAGGAGGGGGAGG 14

||||||||||||||

Sbjct 180 GGAGGAGGGGGAGG 193

>LmjF.30.0940:mRNA | gene=LmjF.30.0940 | organism=Leishmania_major_strain_Friedlin

| gene_product=Gamma-tubulin complex component

4 | transcript_product=Gamma-tubulin complex component

4 | location=LmjF.30:311035-313386(+) | length=2352 | sequence_SO=chromosome

| SO=protein_coding_gene | is_pseudo=false

Length=2352

Score = 26.5 bits (28), Expect = 8.5

Identities = 14/14 (100%), Gaps = 0/14 (0%)

Strand=Plus/Plus

Query 10 GGAGGCGCTTGCGG 23

||||||||||||||

Sbjct 1635 GGAGGCGCTTGCGG 1648

>LmjF.29.0790:mRNA | gene=LmjF.29.0790 | organism=Leishmania_major_strain_Friedlin

| gene_product=hypothetical protein, conserved

| transcript_product=hypothetical protein, conserved

| location=LmjF.29:288015-292784(-) | length=4770 | sequence_SO=chromosome

| SO=protein_coding_gene | is_pseudo=false

Length=4770

Score = 26.5 bits (28), Expect = 8.5

Identities = 14/14 (100%), Gaps = 0/14 (0%)

Strand=Plus/Minus

Query 1 GGAGGAGGGGGAGG 14

||||||||||||||

Sbjct 2274 GGAGGAGGGGGAGG 2261

>LmjF.27.0370:mRNA | gene=LmjF.27.0370 | organism=Leishmania_major_strain_Friedlin

| gene_product=hypothetical protein, conserved

| transcript_product=hypothetical protein, conserved

| location=LmjF.27:101715-102299(+) | length=585 | sequence_SO=chromosome

| SO=protein_coding_gene | is_pseudo=false

Length=585

Score = 26.5 bits (28), Expect = 8.5

Identities = 14/14 (100%), Gaps = 0/14 (0%)

Strand=Plus/Plus

Query 57 GGTGTGTCGGCGAC 70

||||||||||||||

Sbjct 262 GGTGTGTCGGCGAC 275

>LmjF.25.1110:mRNA | gene=LmjF.25.1110 | organism=Leishmania_major_strain_Friedlin

| gene_product=hypothetical protein, conserved

| transcript_product=hypothetical protein, conserved

| location=LmjF.25:429674-449296(-) | length=19623 | sequence_SO=chromosome

| SO=protein_coding_gene | is_pseudo=false

Length=19623

Score = 26.5 bits (28), Expect = 8.5

Identities = 14/14 (100%), Gaps = 0/14 (0%)

Strand=Plus/Minus

Query 1 GGAGGAGGGGGAGG 14

||||||||||||||

Sbjct 16398 GGAGGAGGGGGAGG 16385

>LmjF.25.0870:mRNA | gene=LmjF.25.0870 | organism=Leishmania_major_strain_Friedlin

| gene_product=Atypical serine/threonine

protein kinase BUD32, putative | transcript_product=Atypical

serine/threonine protein kinase BUD32, putative | location=LmjF.25:336027-336905(+)

| length=879 | sequence_SO=chromosome

| SO=protein_coding_gene | is_pseudo=false

Length=879

Score = 26.5 bits (28), Expect = 8.5

Identities = 14/14 (100%), Gaps = 0/14 (0%)

Strand=Plus/Minus

Query 33 CGATCATGGTGCGC 46

||||||||||||||

Sbjct 874 CGATCATGGTGCGC 861

>LmjF.24.1280:mRNA | gene=LmjF.24.1280 | organism=Leishmania_major_strain_Friedlin

| gene_product=amastin-like surface protein-like

protein | transcript_product=amastin-like surface

protein-like protein | location=LmjF.24:454236-454796(+) | length=561

| sequence_SO=chromosome | SO=protein_coding_gene

| is_pseudo=false

Length=561

Score = 26.5 bits (28), Expect = 8.5

Identities = 17/19 (89%), Gaps = 0/19 (0%)

Strand=Plus/Minus

Query 31 GGCGATCATGGTGCGCGGG 49

|| ||||||||||| ||||

Sbjct 366 GGAGATCATGGTGCACGGG 348

>LmjF.24.1250:mRNA | gene=LmjF.24.1250 | organism=Leishmania_major_strain_Friedlin

| gene_product=amastin-like surface protein-like

protein | transcript_product=amastin-like surface

protein-like protein | location=LmjF.24:444065-444625(+) | length=561

| sequence_SO=chromosome | SO=protein_coding_gene

| is_pseudo=false

Length=561

Score = 26.5 bits (28), Expect = 8.5

Identities = 17/19 (89%), Gaps = 0/19 (0%)

Strand=Plus/Minus

Query 31 GGCGATCATGGTGCGCGGG 49

|| ||||||||||| ||||

Sbjct 366 GGAGATCATGGTGCACGGG 348

>LmjF.23.1510:mRNA | gene=LmjF.23.1510 | organism=Leishmania_major_strain_Friedlin

| gene_product=V-type proton ATPase subunit

a, putative | transcript_product=V-type proton ATPase subunit

a, putative | location=LmjF.23:741453-743780(+) | length=2328

| sequence_SO=chromosome | SO=protein_coding_gene |

is_pseudo=false

Length=2328

Score = 26.5 bits (28), Expect = 8.5

Identities = 14/14 (100%), Gaps = 0/14 (0%)

Strand=Plus/Plus

Query 1 GGAGGAGGGGGAGG 14

||||||||||||||

Sbjct 1935 GGAGGAGGGGGAGG 1948

>LmjF.20.0290:mRNA | gene=LmjF.20.0290 | organism=Leishmania_major_strain_Friedlin

| gene_product=E3 ubiquitin-protein ligase

KCMF1, putative | transcript_product=E3 ubiquitin-protein

ligase KCMF1, putative | location=LmjF.20:105367-107766(+)

| length=2400 | sequence_SO=chromosome | SO=protein_coding_gene

| is_pseudo=false

Length=2400

Score = 26.5 bits (28), Expect = 8.5

Identities = 14/14 (100%), Gaps = 0/14 (0%)

Strand=Plus/Minus

Query 56 CGGTGTGTCGGCGA 69

||||||||||||||

Sbjct 1365 CGGTGTGTCGGCGA 1352

>LmjF.16.1190:mRNA | gene=LmjF.16.1190 | organism=Leishmania_major_strain_Friedlin

| gene_product=Domain of unknown function

DUF221, putative | transcript_product=Domain of unknown function

DUF221, putative | location=LmjF.16:469429-473754(-)

| length=4326 | sequence_SO=chromosome | SO=protein_coding_gene

| is_pseudo=false

Length=4326

Score = 26.5 bits (28), Expect = 8.5

Identities = 17/19 (89%), Gaps = 0/19 (0%)

Strand=Plus/Minus

Query 5 GAGGGGGAGGCGCTTGCGG 23

||||||||||||| ||||

Sbjct 3317 GAGGGGGAGGCGCCGGCGG 3299

>LmjF.16.1120:mRNA | gene=LmjF.16.1120 | organism=Leishmania_major_strain_Friedlin

| gene_product=hypothetical protein, conserved

| transcript_product=hypothetical protein, conserved

| location=LmjF.16:452839-453651(+) | length=813 | sequence_SO=chromosome

| SO=protein_coding_gene | is_pseudo=false

Length=813

Score = 26.5 bits (28), Expect = 8.5

Identities = 14/14 (100%), Gaps = 0/14 (0%)

Strand=Plus/Minus

Query 1 GGAGGAGGGGGAGG 14

||||||||||||||

Sbjct 196 GGAGGAGGGGGAGG 183

>LmjF.13.1650:mRNA | gene=LmjF.13.1650 | organism=Leishmania_major_strain_Friedlin

| gene_product=dynein heavy chain, putative

| transcript_product=dynein heavy chain, putative | location=LmjF.13:624630-638624(-)

| length=13995 | sequence_SO=chromosome

| SO=protein_coding_gene | is_pseudo=false

Length=13995

Score = 26.5 bits (28), Expect = 8.5

Identities = 20/24 (83%), Gaps = 0/24 (0%)

Strand=Plus/Plus

Query 1 GGAGGAGGGGGAGGCGCTTGCGGA 24

||| | || | |||||||||||||

Sbjct 11646 GGACGCGGTGCAGGCGCTTGCGGA 11669

>LmjF.13.1600:mRNA | gene=LmjF.13.1600 | organism=Leishmania_major_strain_Friedlin

| gene_product=hypothetical protein, conserved

| transcript_product=hypothetical protein, conserved

| location=LmjF.13:599546-603487(-) | length=3942 | sequence_SO=chromosome

| SO=protein_coding_gene | is_pseudo=false

Length=3942

Score = 26.5 bits (28), Expect = 8.5

Identities = 17/19 (89%), Gaps = 0/19 (0%)

Strand=Plus/Plus

Query 55 GCGGTGTGTCGGCGACAAG 73

||| ||| |||||||||||

Sbjct 1463 GCGATGTATCGGCGACAAG 1481

>LmjF.07.0480:mRNA | gene=LmjF.07.0480 | organism=Leishmania_major_strain_Friedlin

| gene_product=Dynein heavy chain, N-terminal

region 2, putative | transcript_product=Dynein heavy

chain, N-terminal region 2, putative | location=LmjF.07:216483-231029(+)

| length=14547 | sequence_SO=chromosome | SO=protein_coding_gene

| is_pseudo=false

Length=14547

Score = 26.5 bits (28), Expect = 8.5

Identities = 14/14 (100%), Gaps = 0/14 (0%)

Strand=Plus/Plus

Query 1 GGAGGAGGGGGAGG 14

||||||||||||||

Sbjct 8124 GGAGGAGGGGGAGG 8137

>LmjF.05.0870:mRNA | gene=LmjF.05.0870 | organism=Leishmania_major_strain_Friedlin

| gene_product=hypothetical protein, conserved

| transcript_product=hypothetical protein, conserved

| location=LmjF.05:316682-322450(+) | length=5769 | sequence_SO=chromosome

| SO=protein_coding_gene | is_pseudo=false

Length=5769

Score = 26.5 bits (28), Expect = 8.5

Identities = 14/14 (100%), Gaps = 0/14 (0%)

Strand=Plus/Minus

Query 1 GGAGGAGGGGGAGG 14

||||||||||||||

Sbjct 5136 GGAGGAGGGGGAGG 5123

>LmjF.04.0980:mRNA | gene=LmjF.04.0980 | organism=Leishmania_major_strain_Friedlin

| gene_product=hypothetical protein, conserved

| transcript_product=hypothetical protein, conserved

| location=LmjF.04:379216-382821(-) | length=3606 | sequence_SO=chromosome

| SO=protein_coding_gene | is_pseudo=false

Length=3606

Score = 26.5 bits (28), Expect = 8.5

Identities = 14/14 (100%), Gaps = 0/14 (0%)

Strand=Plus/Minus

Query 10 GGAGGCGCTTGCGG 23

||||||||||||||

Sbjct 924 GGAGGCGCTTGCGG 911

Lambda K H

0.634 0.408 0.912

Gapped

Lambda K H

0.625 0.410 0.780

Effective search space used: 824380400

Database: /eupath/data/apiSiteFilesStaging/TriTrypDB/52/real/webServices/TriTr

ypDB/release-

CURRENT/LmajorFriedlin/blast/LmajorFriedlinAnnotatedTranscripts

Posted date: Dec 16, 2021 1:23 PM

Number of letters in database: 16,705,993

Number of sequences in database: 9,495

Matrix: blastn matrix 2 -3

Gap Penalties: Existence: 5, Extension: 2
